# The Synthesis of a Naloxone-Related Oxidative Drug Product Degradant

**DOI:** 10.1021/acs.joc.5c00313

**Published:** 2025-04-14

**Authors:** Marie-Angélique F. S. Deschamps, John S. Carey, Joseph P. A. Harrity

**Affiliations:** † Division of Chemistry, School of Mathematical and Physical Sciences, University of Sheffield, Brook Hill, Sheffield S3 7HF, U.K.; ‡ Indivior UK Ltd, Henry Boot Way, Hull HU4 7DY, U.K.

## Abstract

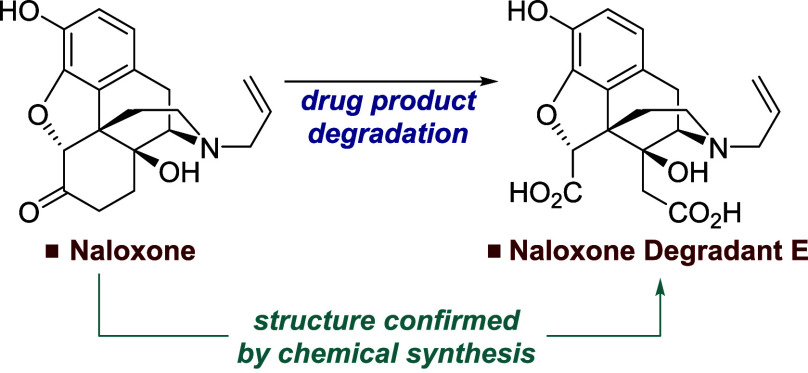

Naloxone is a nonselective opioid receptor antagonist used to reverse the effects of opiate-related overdose. Studies aimed toward identifying naloxone degradants present in a buprenorphine/naloxone combination drug product revealed several compounds whose structures could not be confirmed by comparison to authentic samples. We report herein the confirmation of the structural assignment of one of these compounds (so-called, “Degradant E”) by chemical synthesis starting from naloxone. Key features of the developed route include the conversion of the *N*-allyl group to the corresponding Boc carbamate as a means of facilitating the chemoselective oxidative cleavage of the C6–C7 bond. In addition, the use of a pivalate ester derivative of naloxone’s phenol group offered a convenient means of isolating Degradant E as the corresponding HCl salt using an acid-promoted global ester hydrolysis in the final step.

## Introduction

Naloxone is a nonselective opioid receptor antagonist that is used to reverse the effects of opiate-related overdose and is included in the World Health Organization’s list of essential medicines.^1^ It is also used as a treatment against opioid disorder (addiction) and was approved by the Food and Drug Administration in 1971 in the US, under the name Narcan. During the development of a novel drug product to decrease the risk of opioid misuse, a combination product containing both naloxone and buprenorphine (as a μ-opioid receptor partial agonist) was developed. In line with International Conference on Harmonisation (ICH) guidelines on the technical requirements for pharmaceuticals for human use,^2^ extensive stability studies to confirm the quality, safety, and efficacy of this drug product and to define an accurate and appropriate shelf life were undertaken. These studies highlighted the formation of 15 naloxone related degradants at or above the reporting threshold.^3^ Structures were assigned using a combination of mass spectrometry and NMR spectroscopy. However, only 5 of these could have their structures fully confirmed by comparison to an authentic sample. For the remaining 10 degradants, where authentic samples were not available, we considered chemical synthesis as a means to confirm the structural assignments.^4^

**Scheme 1:**
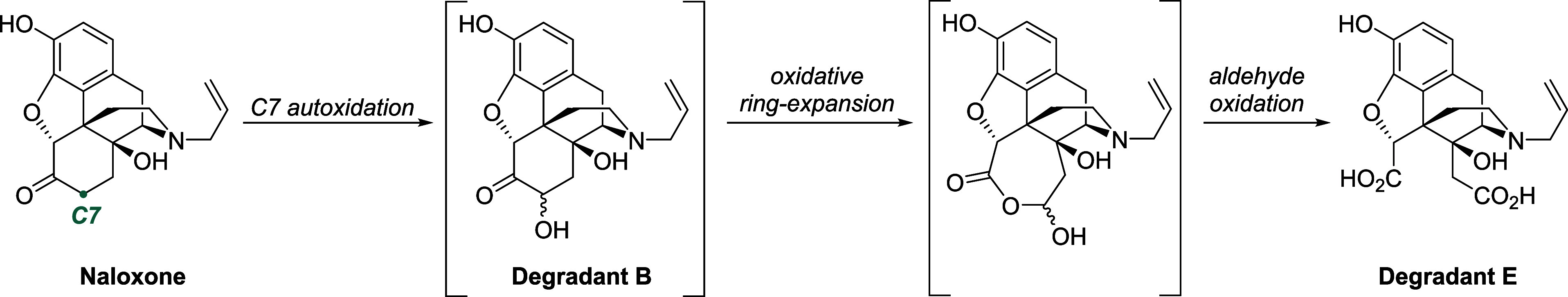
Degradation Pathway to Degradant E

In deciding which degradants to target, we were particularly intrigued by the formation of a compound that appeared to have been generated by the oxidative cleavage of the cyclohexanone ring of naloxone (so-called, “Degradant E”). As shown in Scheme 1, this compound was believed to derive from the oxidation of naloxone at C7 (naloxone numbering) to form “Degradant B”, followed by an oxidative ring expansion via a Baeyer–Villiger type reaction. Unlike the majority of other degradation products, this primary oxidation degradant (“Degradant B”) could not be isolated and fully characterized. Rather its structural assignment was based solely upon mass spectrometry data. The uncertainty around the structure of Degradant B together with the intriguing apparent mechanism of formation of Degradant E prompted us to prioritize the confirmation of the structural assignment of the latter compound. We report herein the successful synthesis of this compound by the chemoselective modification of naloxone and hence confirm the structure of Degradant E.

## Results and Discussion

**Scheme 2:**
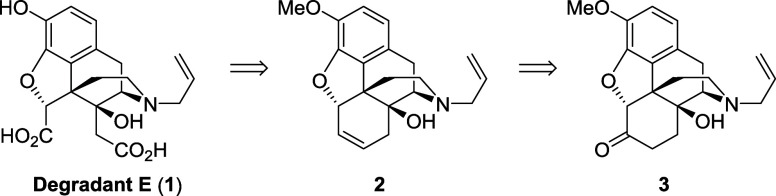
Retrosynthetic Analysis for Degradant E Starting from Naloxone Methyl Ether

Our retrosynthetic analysis of Degradant E (**1**) toward naloxone as a starting point is depicted in Scheme 2. It was envisaged that the diacid would result from an oxidative cleavage of the 6,7-alkene **2**, which would be obtained in a few steps from naloxone methyl ether **3**.^5^

**Scheme 3:**
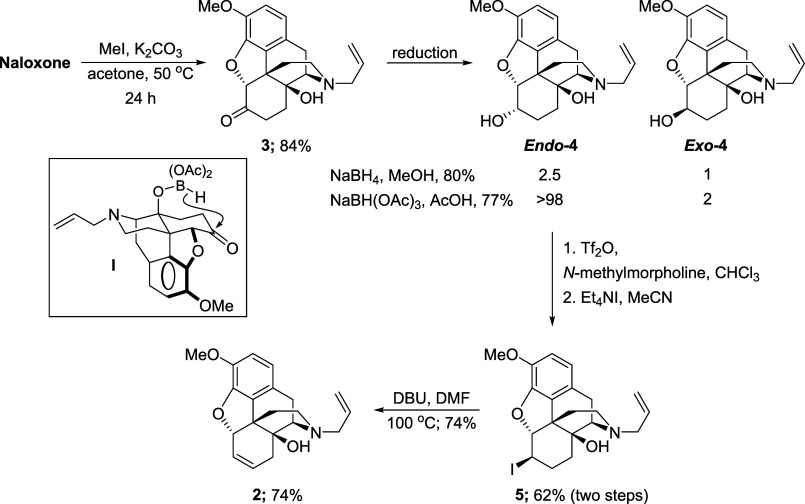
Synthesis of **6**,**7**-Alkene **2**

The synthesis of the 6,7-alkene was inspired by the work of Nagase and coworkers in their synthesis of (−)-homogalanthamine from naltrexone.^6^ Accordingly, the ketone reduction of methyl ether **3** was studied using sodium borohydride and sodium (triacetoxy)borohydride (Scheme 3). Using sodium borohydride, a mixture of the two alcohol diastereoisomers **4** was obtained in 80% yield and a 2.5:1 endo/exo ratio. Interestingly, however, using sodium (triacetoxy)borohydride resulted in endo isomer **4** being obtained in 82% yield as a single diastereomer, presumably because of a substrate directed reduction as shown in **I**. Elimination of the *endo*-alcohol was known to be problematic due to competing pathways (formation of desired allylic ether **2** versus enol ether, and cyclic ether formation via the free alcohol^6^) and so a modification of Nagase’s route was used to access **2** in good yield over three steps via the *exo*-iodide **5**.

**Scheme 4:**
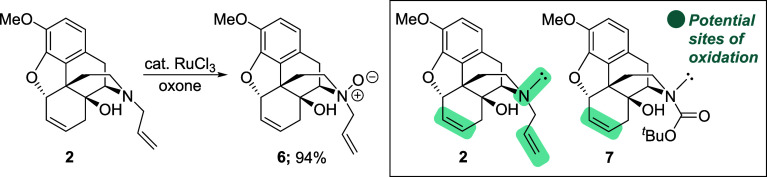
Attempted Oxidative Cleavage of **6**,**7**-Alkene **2**

A study of the key oxidative cleavage step that would transform the cyclohexene moiety of **2** to the diacid fragment of Degradant E was undertaken. In the event, treatment of **2** with catalytic RuCl_3_ and Oxone resulted in the formation of a new product that was tentatively characterized as the corresponding *N*-oxide **6** (Scheme 4),^7^ whereas attempts to oxidize the cyclohexene unit by dihydroxylation (catalytic OsO_4_, NMO) or epoxidation (*m*-CPBA) gave mixtures from which the desired products could not be cleanly isolated. As a chemoselective oxidation of the cyclic alkene in the presence of the allylic amine was proving to be problematic, it was decided to target the synthesis of carbamate **7** that would pave the way for a more straightforward functionalization of the cyclohexene moiety.

**Scheme 5:**
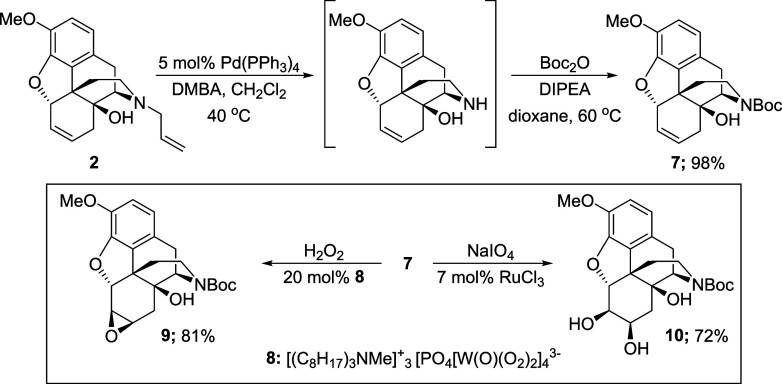
Oxidation of **6**,**7**-Alkene **7**^a^ DMBA: *N*,*N*-dimethylbarbituric acid. DIPEA, diisopropylethylamine.

Accordingly, a Pd-catalyzed deallylation^8^ followed by a amine protection was performed that afforded the *N*-Boc alkene **7** in 98% yield over two steps (Scheme 5). The alkene oxidation step was reinvestigated and pleasingly the use of a (diperoxotungsto)phosphate catalyst **8** in conjunction with hydrogen peroxide, first reported by D’Aloisio,^9^ allowed the epoxide **9** to be isolated in 81% yield. Moreover, the alkene in **7** was also smoothly dihydroxylated by sodium periodate in the presence of ruthenium(III) chloride, allowing the corresponding diol **10** to be generated in 72% yield.^10^ As vicinal diol **10** offered several possibilities for oxidative cleavage it was decided to continue the synthesis from this intermediate.

With regard to the oxidative cleavage step, we were attracted by the report of Iwabuchi and coworkers that described the oxidative cleavage of a range of cyclic and acyclic vicinal diols into the corresponding dicarboxylic acids using (diacetoxy)iodobenzene and 1-methyl-2-azaadamantane *N*-oxyl (1-Me-AZADO) as the catalyst.^11^ Two general strategies were apparent; the oxidative cleavage of **10** followed by conversion of the *N*-Boc group to the allylic amine, or Boc deprotection and allylation of **10** followed by oxidative cleavage of the diol.

**Scheme 6:**
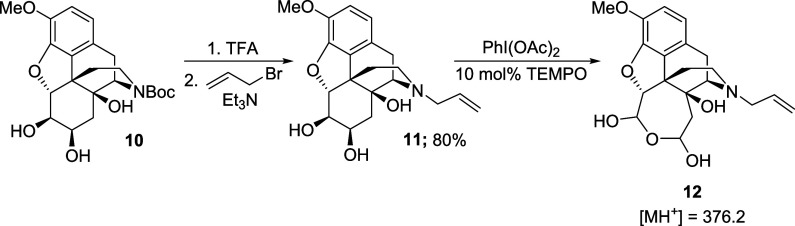
Attempted oxidative cleavage of diol **11**

Beginning with the latter approach, the *N*-allyl diol **11** was readily obtained in 80% yield after Boc deprotection and allylation of the secondary amine with allyl bromide (Scheme 6). Subjection of this compound to PhI(OAc)_2_/catalytic 2,2,6,6-tetramethylpiperidine 1-oxyl (TEMPO; in place of 1-Me-AZADO) resulted in slow conversion to a new compound with a mass of *m*/*z* = 375 (as judged by liquid chromatography–mass spectrometry (LC-MS) analysis), which we assigned as cyclic hydrated bis-aldehyde **12**. However, this compound was formed in low yield and could not be isolated and fully characterized. Furthermore, extending the reaction time failed to significantly improve conversion, and so, this route was abandoned.

**Scheme 7:**
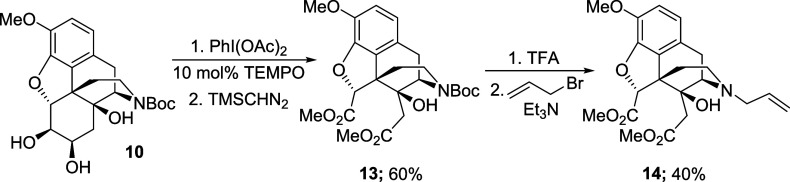
Oxidative Cleavage of Diol **10**

Moving to the alternative sequence, the oxidative cleavage of the *N*-Boc diol **10** was carried out using the TEMPO-catalyzed method (Scheme 7). In this case, the product was detected by LC-MS analysis, but it could not be isolated, probably due to its high water solubility. In order to avoid this problem, the crude mixture was treated with an excess of (trimethylsilyl)diazomethane and the corresponding *N*-Boc diester **13** was isolated in 60% yield. Finally, we reinstalled the allylic amine unit by treating compound **13** with trifluoroacetic acid and alkylation of the secondary amine with allyl bromide to give the *N*-allyl diester **14** in 40% yield.

**Scheme 8:**
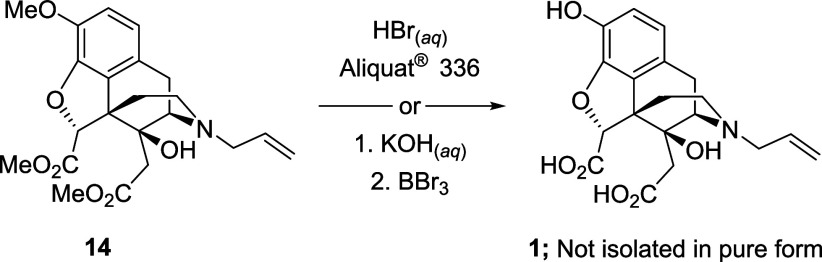
Attempted Global Deprotection of Diester **14**

The final step of the synthesis required hydrolysis of the methyl esters and methyl ether (Scheme 8). We first used a combination of aqueous HBr with a phase transfer catalyst (trioctylmethylammonium chloride, Aliquat 336).^12^ HPLC analysis of the reaction mixture revealed that the desired diacid **1** was present, but only as a minor component in a mixture containing various partially demethylated compounds, and it proved impossible to obtain an analytically pure sample of **1** from this mixture. As an alternative approach, diester **14** was hydrolyzed using a solution of potassium hydroxide in methanol. This reaction was successful, and the conversion of the diester **14** to the corresponding diacid proceeded with complete conversion. However, the subsequent deprotection of the methyl ether using boron tribromide (BBr_3_) was unsuccessful. In this case, only the starting material was recovered. We attributed this observation to the low solubility of the diacid in organic solvents.

At this final stage of the synthesis, we were frustrated to find that the methyl ether was not a viable protecting group for the clean isolation of degradant E (**1**). Given that the last step of the synthesis is the deprotection of the two esters, we wondered if the sequence could be repeated with a more acid- or base-sensitive phenol-protecting group.

It was decided to repeat the optimized sequence using a pivalate ester as the phenol-protecting group, in place of the methyl ether, as we assumed that this protecting group would be robust enough to withstand the various transformations required for the synthesis, yet still be readily hydrolyzed at the final step.

**Scheme 9:**
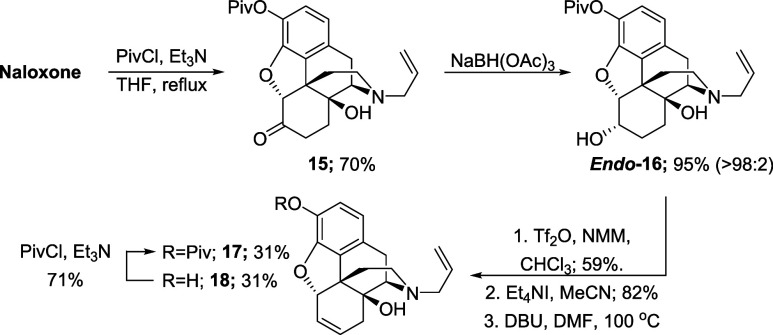
Synthesis of **6**,**7**-Alkene **17**

Accordingly, using pivaloyl chloride, naloxone was transformed into the corresponding ester **15** in 70% yield (Scheme 9). Reduction of the ketone afforded the corresponding alcohol **16** in 95%. Formation of the triflate followed by substitution with tetraethylammonium iodide proceeded in 48% over two steps. The elimination step proved to be more problematic with the pivalate derivative, as compared to the corresponding methoxy compound; a mixture of alkene regioisomers was detected by NMR spectroscopy, which led to difficult separation by column chromatography. In addition, the desired allylic ether **17** was isolated in 31% yield along with the corresponding free phenol **18** (also isolated in 31%), which was not fully characterized, but instead transformed back the pivalate ester **17** in good yield.

**Scheme 10:**
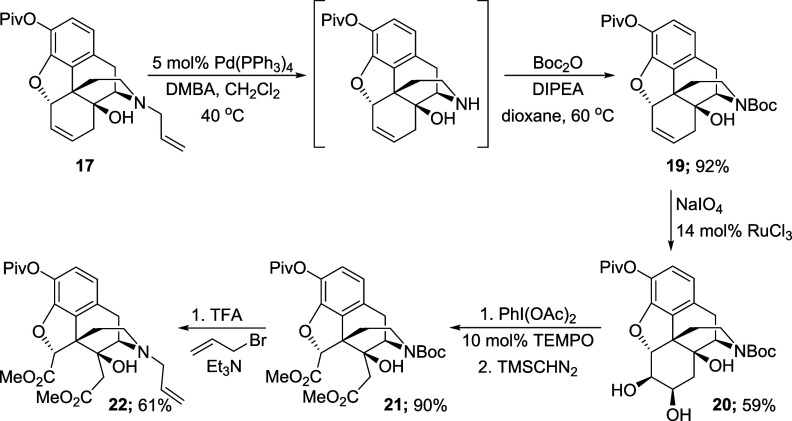
Synthesis of Triester **22**

The remaining steps to elaborate **17** to the Degradant E precursor were relatively uneventful. The deallylation of **17** and amine protection steps proceeded smoothly to give the *N*-Boc alkene **19** in 92% yield over two steps (Scheme 10). The subsequent dihydroxylation step required an increased catalyst loading to 14 mol % to ensure a complete conversion of the starting alkene, but it produced the corresponding diol **20** in 59% yield. Then, treatment of **20** with PhI(OAc)_2_ and a TEMPO catalyst provided the corresponding dicarboxylic acid, which was subsequently esterified using TMS-diazomethane to afford the *N*-Boc diester **21** in 90% yield over two steps. Finally, the Boc group was removed with TFA and the resulting material directly converted to the *N*-allyl diester **22**, obtained in 61% yield, over two steps.

**Scheme 11:**
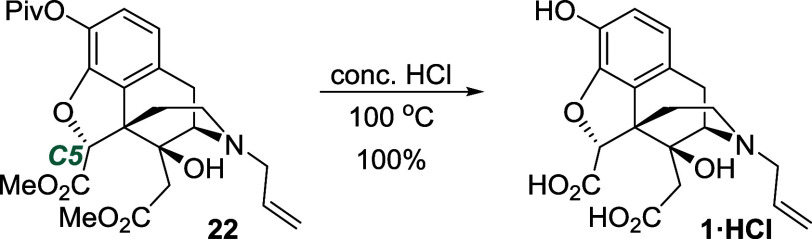
Hydrolysis of Triester **22** to give Compound **1**

This sequence led us to the last step, which involved global hydrolysis of the esters. First, a saponification reaction using potassium hydroxide in methanol was attempted. LC-MS analysis indicated the presence of the expected mass ion, however, a mixture of two compounds was detected by NMR spectroscopy in a 2.5:1 ratio. It was surmized that, under basic conditions, C5 might be prone to epimerization. Accordingly, acid-promoted hydrolysis was investigated instead. Pleasingly, the use of concentrated HCl at elevated temperatures led to the formation of the hydrochloride salt of diacid **1** in quantitative yield, as a single compound (Scheme 11).

**Figure 1:**
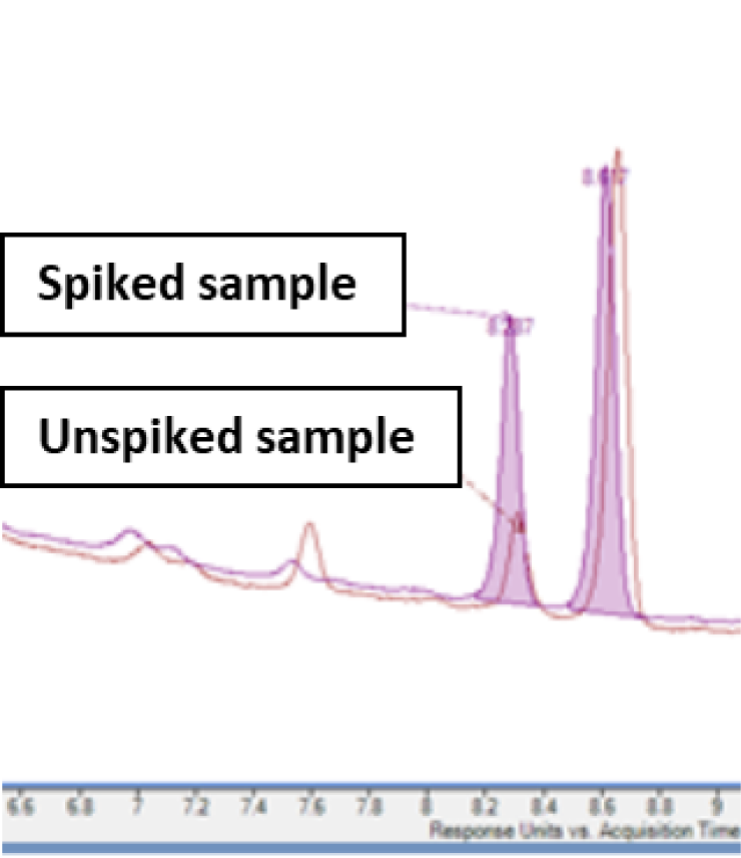
Overlay of a portion of the HPLC chromatogram of the degradant drug product along with a sample of the degradant product spiked with compound 1.

Finally, the diacid **1** was analyzed by LC-MS to confirm that the authentic sample had the same HPLC retention time and same accurate mass and fragmentation pattern as Degradant E and thus confirm that the assigned structure was correct. The NMR data for the authentic sample of diacid **1** was consistent with the sample prepared by forced degradation.^3^Figure 1 shows the HPLC trace of the degraded drug product (unspiked sample) and the HPLC trace of compound **1** added to the degraded sample (spiked sample). Only the peak at 8.3 min showed an increase in intensity due to the increased concentration of Degradant E in the spiked sample, which confirmed the structure of this impurity in the degraded drug product.

## Conclusions

In conclusion, the synthesis of naloxone Degradant E has been successfully carried out in an overall yield of 5% over 12 steps. The structure of the impurity was confirmed by NMR spectroscopy and mass spectrometry. LCMS analysis confirmed that the authentic sample of diacid **1** had the same HPLC retention time and mass spectrometry fragmentation pattern as Degradant E thus confirming the structural assignment.

## Experimental Section

### General Information

All reactions were carried out in flame-dried glassware equipped with a magnetic stir bar under nitrogen atmosphere, unless stated otherwise. Solvents were purified using a PureSolv MD purification system and transferred under nitrogen. A DrySyn block combined with a temperature probe was used as the heating source, where required. Infrared (IR) spectra were recorded on a PerkinElmer Paragon FTIR spectrometer. ^1^H NMR spectra were recorded on a Bruker AVIII HD 400 (400 MHz), Bruker AVI 400 (400 MHz) or Bruker AMX400 (400 MHz). Chemical shifts are reported in parts per million (ppm) from tetramethylsilane, using the residual protic solvent resonance as the internal reference: (CHCl_3_: δ 7.26) unless otherwise stated. Data are reported as follows: chemical shift (multiplicity (s = singlet, d = doublet, t = triplet, q = quartet, br = broad, m = multiplet), coupling constant (Hz), integration). ^13^C{^1^H} NMR spectra were recorded on a Bruker AVIII HD 400 (101 MHz), Bruker AVI 400 (101 MHz) or Bruker AMX-400 (101 MHz) with broadband proton decoupling. Chemical shifts are reported in ppm from trimethylsilane with the solvent as the internal reference (CDCl_3_: δ 77.16). High resolution mass spectra (HRMS) recorded for accurate mass analysis, were performed on either a Micromass LCT operating in electrospray mode (TOF, ESI^+^) or a Micromass Prospec operating in FAB (FAB^+^), EI (ESI^+^) or CI (CI^+^) mode. Thin layer chromatography (TLC) was performed on aluminum-backed plates pre coated with silica (0.2 mm, Merck 60 F_254_) which were developed using standard visualizing agents: UV light or potassium permanganate. Flash chromatography was performed on silica gel (Merck 40–63 μm). HPLC data was acquired using an Agilent 1200 HPLC system fitted with a Gemini C18, 150 × 3 mm column. Catalyst **8** was prepared according to the procedure described by Venturello.^9^^a^
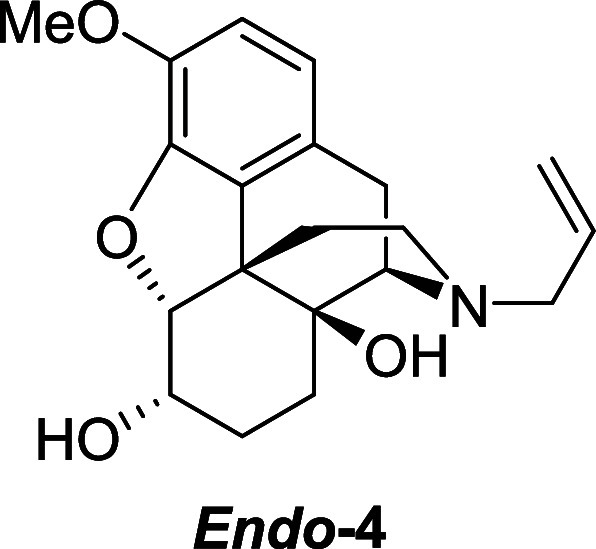


Naloxone methyl ether **3**^5^ (2.0 g, 5.95 mmol) was dissolved in acetic acid (20 mL), under a N_2_ atmosphere. Sodium (triacetoxy)borohydride (3.8 g, 17.85 mmol) was added portion-wise at room temperature and the mixture was stirred for 1 h. Acetone (4 mL) was added and the mixture was stirred for a further hour. The solvent was evaporated, and the pH was adjusted to 9–10 using a 1 M solution of sodium hydroxide. The aqueous phase was extracted with DCM, the organic layer was washed with brine, dried over magnesium sulfate and the solvent was removed under reduced pressure, to afford ***endo-4*** (1.55 g, 77%) as a white foam. ^1^H NMR (400 MHz, CDCl_3_) δ: 6.70 (d, *J* = 8.0 Hz, 1H), 6.58 (d, *J* = 8.0 Hz, 1H), 5.77 (ddt, *J* = 16.5, 10.0, 6.5 Hz, 1H), 5.21–5.10 (m, 2H), 4.61 (d, *J* = 4.5 Hz, 1H), 4.17 (dt, *J* = 10.0, 4.5 Hz, 1H), 3.84 (s, 3H), 3.11–3.07 (m, 2H), 3.07 (d, *J* = 19.0 Hz, 1H) 2.88 (d, *J* = 6.5 Hz, 1H), 2.58 (dd, *J* = 19.0, 6.5 Hz, 1H), 2.54–2.48 (m, 1H), 2.25–2.13 (m, 2H), 1.75 (td, *J* = 12.5, 4.2 Hz, 1H), 1.59 (dt, *J* = 14.5, 8.0 Hz, 1H), 1.53–1.49 (m, 1H), 1.43 (ddd, *J* = 14.5, 8.0, 4.5 Hz, 1H), 1.20–1.09 (m, 1H); ^13^C{^1^H} NMR (101 MHz, CDCl_3_) δ: 146.6, 141.7, 135.4, 131.5, 126.2, 118.9, 117.9, 113.7, 90.8, 70.0, 66.7, 62.4, 58.0, 56.5, 54.2, 43.0, 33.3, 28.3, 23.7, 22.9; HRMS (ESI) *m*/*z*: [M + H]^+^ Calcd for C_20_H_26_NO_4_ 344.1862; Found 344.1861; FTIR (neat, cm^–1^) 3340, 2925, 1724, 1428, 1275, 979.
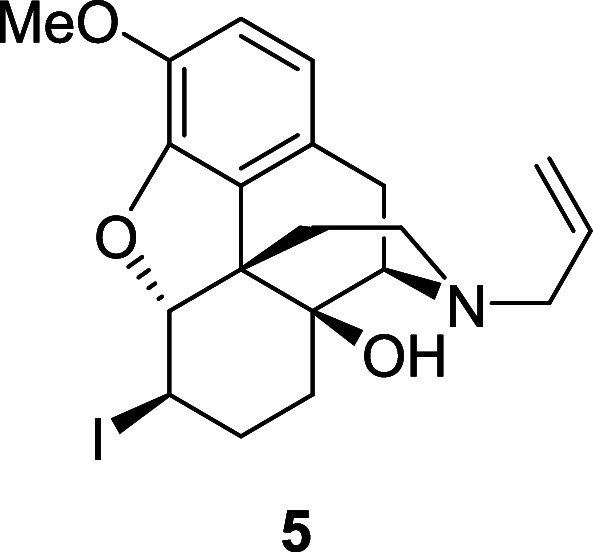


*Endo-***4** (0.5 g, 1.46 mmol) was dissolved in anhydrous chloroform (15 mL) under an argon atmosphere. *N*-Methylmorpholine (0.64 mL, 5.83 mmol) was added and the solution was cooled to −30 °C. Trifluoromethanesulfonic anhydride (0.49 mL, 2.92 mmol) was added dropwise and the solution was stirred from −30 to 0 °C for 1.5 h. The reaction mixture was diluted with chloroform, washed with saturated aqueous sodium hydrogen carbonate, water and brine. The organic phase was dried over magnesium sulfate and the solvent was removed under reduced pressure to afford the triflate **85** (537 mg, 77%) as a brown oil.

The resulting triflate (0.15 g, 0.32 mmol) was dissolved in dry acetonitrile (5.5 mL) under an argon atmosphere. Tetraethylammonium iodide (0.16 g, 0.63 mmol) was added in one portion at −10 °C and the reaction mixture was stirred for 1 h. The reaction mixture was allowed to warm to room temperature and stirred overnight. The solvent was evaporated and the residue was dissolved in chloroform and washed with water. The organic layer was dried over magnesium sulfate and the solvent was evaporated to give compound **5** (114 mg, 80%) as a colorless solid. ^1^H NMR (400 MHz, CDCl_3_) δ: 6.72 (d, *J* = 8.0 Hz, 1H), 6.63 (d, *J* = 8.0 Hz, 1H), 5.77 (ddt, *J* = 16.5, 10.0, 6.5 Hz, 1H), 5.21–5.12 (m, 2H), 4.91 (d, *J* = 8.0 Hz, 1H), 3.89 (s, 3H), 3.86 (ddd, *J* = 8.0, 6.5, 3.0 Hz, 1H), 3.14–3.09 (m, 2H), 3.05 (d, *J* = 18.5 Hz, 1H), 2.86 (d, *J* = 5.5 Hz, 1H), 2.60 (td, *J* = 13.0, 3.0 Hz, 1H), 2.56 (dd, *J* = 18.5, 5.5 Hz, 1H), 2.52 (dd, *J* = 12.5, 5.0 Hz, 1H), 2.20 (td, *J* = 12.5, 5.0 Hz, 1H), 2.11–2.02 (m, 2H), 1.46–1.39 (m, 1H), 1.43 (dd, *J* = 13.0, 3.0 Hz, 1H), 1.33 (td, *J* = 13.0, 3.0 Hz, 1H); ^13^C{^1^H} NMR (101 MHz, CDCl_3_) δ: 144.1, 143.6, 135.3, 131.0, 125.6, 119.2, 118.1, 115.3, 96.6, 69.9, 62.6, 57.7, 57.3, 48.8, 43.9, 33.7, 31.8, 30.6, 29.3, 22.8; HRMS (ESI) *m*/*z*: [M + H]^+^ Calcd for C_20_H_25_INO_3_ 454.0879; Found 454.0877; FTIR (neat, cm^–1^) 3391, 2920, 2832, 1498, 1277, 979.
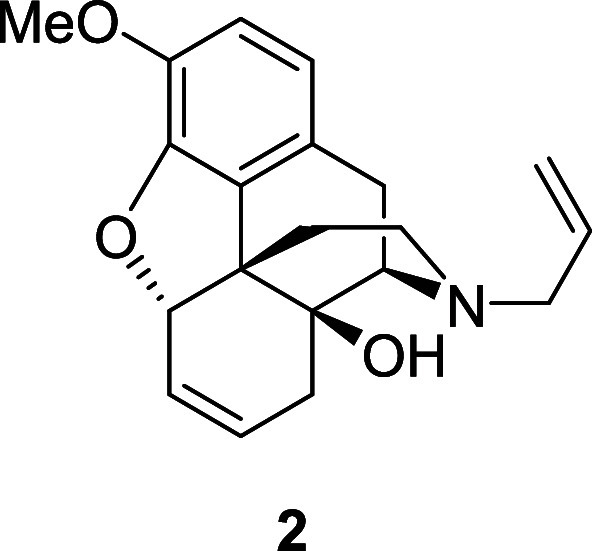


Iodide **5** (3.1 g, 6.86 mmol) was dissolved in dry DMF (34 mL) under a nitrogen atmosphere. DBU (19 mL, 124 mmol) was added and the solution was stirred at 100 °C for 24 h. The crude mixture was poured into a saturated aqueous solution of sodium hydrogen carbonate, diluted with water, and extracted three times with DCM. The combined organic layers were washed with brine, dried over magnesium sulfate, and the solvent was removed under reduced pressure. The crude material was purified by flash chromatography on silica gel, eluted with 10% to 20% ethyl acetate in petroleum ether, to afford the alkene **2** as a colorless oil (1.6 g, 74%). ^1^H NMR (400 MHz, CDCl_3_) δ: 6.64 (d, *J* = 8.0 Hz, 1H), 6.56 (d, *J* = 8.0 Hz, 1H), 5.82–5.68 (m, 3H), 5.19–5.09 (m, 2H), 4.95–4.92 (m, 1H), 4.54 (br, 1H), 3.78 (s, 3H), 3.11–3.06 (m, 2H), 3.07 (d, *J* = 17.5 Hz, 1H), 2.90 (d, *J* = 6.5 Hz, 1H), 2.61 (dd, *J* = 17.5, 6.5 Hz, 1H), 2.55–2.50 (m, 1H), 2.23–2.13 (m, 2H), 2.02–1.89 (m, 2H), 1.62–1.53 (m, 1H); ^13^C{^1^H} NMR (101 MHz, CDCl_3_) δ: 144.7, 143.4, 135.4, 131.6, 129.8, 125.8, 124.2, 118.3, 117.9, 113.4, 87.2, 70.7, 61.9, 58.0, 56.4, 45.2, 43.6, 31.9, 31.1, 22.9; HRMS (ESI) *m*/*z*: [M + H]^+^ Calcd for C_20_H_24_NO_3_ 326.1756; Found 326.1753; FTIR (neat, cm^–1^) 3401, 2922, 1504, 1280, 902.
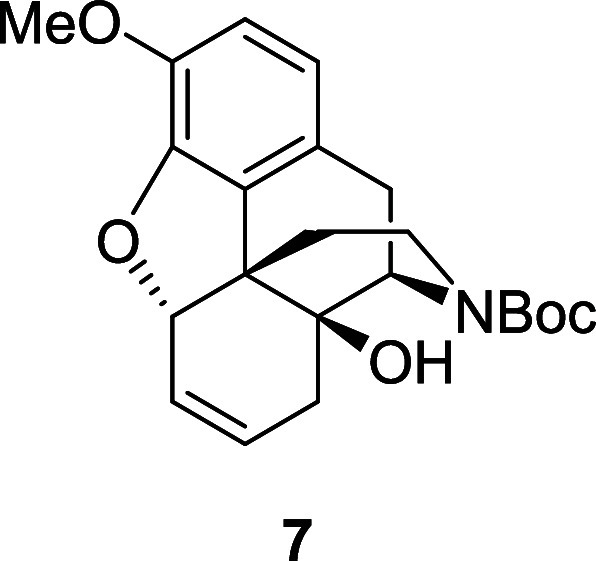


A solution of alkene **2** (0.49 g, 1.504 mmol) in anhydrous DCM (7 mL) was added to a solution of Pd(PPh_3_)_4_ (87 mg, 0.075 mmol) and *N,N*-dimethylbarbituric acid (0.35 g, 2.256 mmol) in anhydrous DCM (8 mL) under a nitrogen atmosphere. The solution was heated at 40 °C and stirred for 16 h, after which the solvent was removed and the crude mixture was dissolved in 1,4-dioxane (15 mL). Di-*tert*-butyl dicarbonate (0.66 g, 3 mmol) and *N,N*-diisopropylethylamine (0.026 mL, 0.15 mmol) were added and the solution was stirred at 60 °C for 5 h. After cooling to room temperature, the solution was poured into water and extracted three times with ethyl acetate. The combined organic layers were washed with brine, dried over magnesium sulfate and the solvent was evaporated. The crude material was purified by flash chromatography on silica gel, eluting with 50% ethyl acetate in petroleum ether to afford the *N*-Boc alkene **7** (0.57 g, 98%) as a light-yellow foam, and as a 64:36 mixture of rotamers. ^1^H NMR (400 MHz, CDCl_3_) δ: 6.72 (d, *J* = 8.0 Hz, 1H), 6.60 (d, *J* = 8.0 Hz, 1H), 5.86–5.75 (m, 2H), 5.00–4.94 (m, 1H), 4.50 (br, 0.6 H, major), 4.32 (br, 0.4 H minor), 3.91 (br, 1H), 3.85 (s, 3H), 3.16 (dd, *J* = 18.5, 6.5 Hz, 1H), 2.91 (d, *J* = 18.5 Hz, 1H), 2.85 (br, 1H), 2.51 (br, 1H), 2.27 (br, 1H), 2.11–1.97 (m, 2H), 1.57 (br, 1H), 1.48 (s, 9H); ^13^C{^1^H} NMR (101 MHz, CDCl_3_) δ: 156.5, 144.8, 143.7, 130.8, 129.3, 125.0, 124.3, 118.7, 113.8, 86.8, 80.4, 71.4, 56.4, 55.4, 45.2, 38.1, 32.7, 32.3, 29.2, 28.5; HRMS (ESI) *m*/*z*: [M + Na]^+^ Calcd for C_22_H_27_NO_5_Na 408.1787; Found 408.1788; FTIR (neat, cm^–1^) 3443, 2974, 1687, 1421, 1267, 1161, 904.
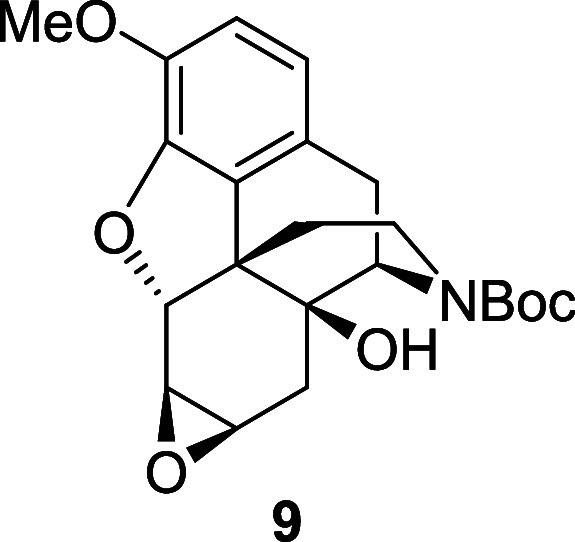


A solution of [(C_8_H_17_)_3_NCH_3_]^+3^[PO_4_[W(O)(O_2_)_2_]_4_^3–^**8** (0.19 g, 0.084 mmol) in DCE (4.2 mL) and water (0.2 mL) was added to a solution of alkene **7** (0.16 g, 0.42 mmol) and hydrogen peroxide (30% in water, 0.14 mL, 1.26 mmol). The reaction mixture was heated at 80 °C for 7 h. After cooling down to room temperature, a saturated solution of sodium thiosulfate was added, and the mixture was stirred for 15 min. The layers were separated, and the crude product was extracted three times with DCM. The combined organic extracts were washed with brine, dried over magnesium sulfate and the solvent was removed under reduced pressure. The crude material was purified by flash chromatography on silica gel eluting with 40% to 50% ethyl acetate in petroleum ether to afford the epoxide **9** (0.14 g, 81%) as a white foam, and as a 58:42 mixture of rotamers. ^1^H NMR (400 MHz, CDCl_3_) δ: 6.76 (d, *J* = 8.0 Hz, 1H), 6.65 (d, *J* = 8.0 Hz, 1H), 4.82 (t, *J* = 1.0 Hz, 1H), 4.54 (d, *J* = 6.0 Hz, 0.6H major), 4.32 (d, *J* = 6.0 Hz, 0.4H minor), 4.04–3.97 (m, 0.4H minor), 3.87 (s, 3H), 3.92–3.82 (m, 0.6H major), 3.32 (dd, *J* = 3.0, 1.0 Hz, 1H), 3.24 (t, *J* = 3.0 Hz, 1H), 3.07 (dd, *J* = 18.5, 6.0 Hz, 1H), 2.87 (d, *J* = 18.5 Hz, 1H), 2.79 (td, *J* = 13.0, 3.5 Hz, 0.6H major), 2.69 (td, *J* = 13.0, 3.5 Hz, 0.4H minor), 2.34 (td, *J* = 13.0, 5.5 Hz, 1H), 2.16 (dd, *J* = 15.5, 1.5 Hz, 0.6H major), 2.09 (dd, *J* = 15.5, 2.0 Hz, 0.4H minor), 1.69 (d, *J* = 15.5 Hz, 1H), 1.46 (s, 9H), 1.35 (dd, *J* = 13.0, 3.5 Hz, 1H); ^13^C{^1^H} NMR (101 MHz, CDCl_3_) δ: 155.6, 144.8, 142.9, 129.1, 125.5, 125.3, 119.7, 114.1, 114.0, 85.2, 79.8, 69.3, 56.5, 55.7, 54.2, 53.5, 52.7, 46.0, 37.7, 36.5, 32.2, 32.1, 30.2, 30.0, 29.8, 28.5; HRMS (ESI) *m*/*z*: [M + Na]^+^ Calcd for C_22_H_27_NO_6_Na 424.1736; Found 424.1712; FTIR (neat, cm^–1^) 3504, 2929, 1684, 1419, 1284, 1164, 1013.
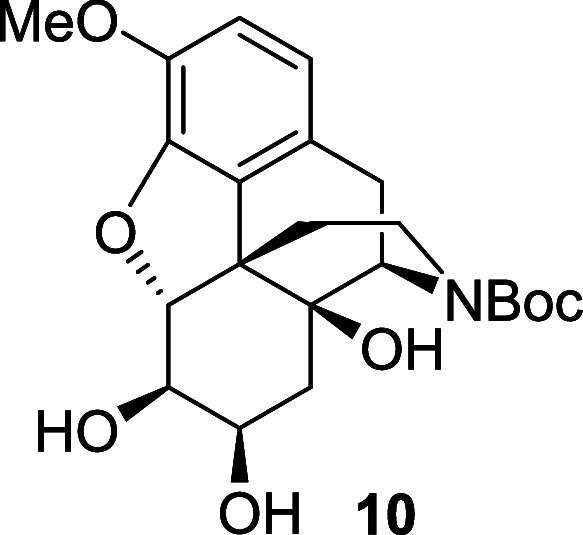


Alkene **7** (0.22 g, 0.575 mmol) was dissolved in ethyl acetate (3.6 mL), acetonitrile (3.6 mL) and water (1.2 mL) and the solution was cooled to 0 °C. RuCl_3_.H_2_O (21 mg, 0.04 mmol) and sodium periodate (0.18 g, 0.86 mmol) were successively added and the reaction mixture was stirred for 45 min. The reaction mixture was allowed to warm to room temperature, a saturated solution of sodium thiosulfate was added and the mixture was stirred for 15 min. The crude product was extracted three times with DCM. The combined organic extracts were washed with brine, dried over magnesium sulfate and the solvent was removed under reduced pressure. The crude material was purified by chromatography on silica gel eluting with 100% ethyl acetate to afford the diol **10** (0.19 g, 72%) as a white foam, and as a 60:40 mixture of rotamers. ^1^H NMR (400 MHz, CDCl_3_) δ: 6.74 (d, *J* = 8.0 Hz, 1H), 6.63 (d, *J* = 8.0 Hz, 1H), 4.61 (d, *J* = 6.0 Hz, 1H), 4.51 (br, 0.4H, minor), 4.30 (br, 0.6H, major), 4.02 (br, 1H), 3.87 (br, 1H), 3.86 (s, 3H), 3.45 (br, 1H), 3.03 (dd, *J* = 18.5, 5.5 Hz, 1H), 2.87 (d, *J* = 18.5 Hz, 1H), 2.67 (br, 1H), 2.36 (td, *J* = 12.5, 5.5 Hz, 1H), 2.07 (dd, *J* = 14.5, 4.0 Hz, 1H), 1.60 (dd, *J* = 14.5, 3.0 Hz, 1H), 1.47 (s, 9H), 1.47 (br, 1H); ^13^C{^1^H} NMR (101 MHz, CDCl_3_) δ: 156.1, 144.2, 143.9, 131.3, 124.5, 119.6, 114.6, 93.9) 80.4, 73.8, 72.1, 70.0, 56.7, 55.4, 47.7, 38.1, 34.4, 31.6, 28.7, 28.6; (ESI) *m*/*z*: [M + Na]^+^ Calcd for C_22_H_29_NO_7_Na 442.1842; Found 442.1851; FTIR (neat, cm^–1^) 3383, 2926, 1664, 1417, 1163, 1058.
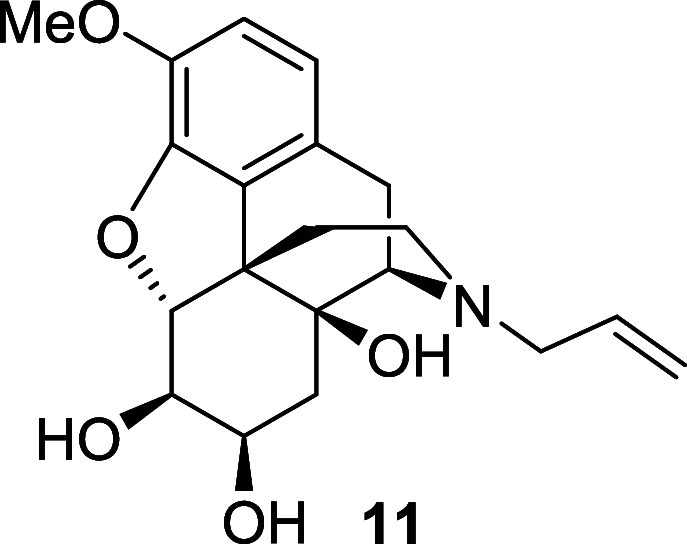


*N*-Boc diol **10** (0.17 g, 0.4 mmol) was dissolved in DCM (4 mL) and TFA (4 mL) was added. The solution was stirred at room temperature for 0.5 h. The solvent was evaporated and the residue dissolved in acetone (4 mL). Allyl bromide (0.07 mL, 0.8 mmol) and triethylamine (0.17 mL, 1.2 mmol) were added and the reaction mixture was heated at 50 °C for 36 h. After cooling to room temperature, an aqueous solution of ammonia was added, and the reaction mixture was stirred for 1 h. The mixture was poured into water and the crude product was extracted with ethyl acetate. The combined organic extracts were washed with brine, dried over magnesium sulfate and the solvent was removed under reduced pressure. The crude material was purified by flash chromatography on silica gel eluting with 5% MeOH in ethyl acetate to afford the *N*-allyl diol **11** (0.12 g, 85%) as a colorless oil. ^1^H NMR (400 MHz, CDCl_3_) δ: 6.71 (d, *J* = 8.0 Hz, 1H), 6.61 (d, *J* = 8.0 Hz, 1H), 5.77 (ddt, *J* = 16.5, 10.0, 6.5 Hz, 1H), 5.22–5.14 (m, 2H), 4.58 (d, *J* = 6.0 Hz, 1H), 3.86 (s, 3H), 3.87–3.83 (m, 1H), 3.38 (dd, *J* = 6.0, 3.5 Hz, 1H), 3.13–3.09 (m, 2H), 3.05 (d, *J* = 18.5 Hz, 1H), 2.97 (d, *J* = 6.0 Hz, 1H), 2.60 (dd, *J* = 18.5, 6.0 Hz, 1H), 2.52 (ddd, *J* = 12.5, 4.0, 2.5 Hz, 1H), 2.22–2.07 (m, 2H), 1.92 (dd, *J* = 14.5, 4.5 Hz, 1H), 1.63 (dd, *J* = 14.5, 3.5 Hz, 1H), 1.54 (dd, *J* = 12.5, 2.5 Hz, 1H); ^13^C{^1^H} NMR (101 MHz, CDCl_3_) δ: 144.1, 143.9, 135.1, 131.5, 124.9, 118.9, 118.4, 114.9, 94.3, 74.3, 72.1, 69.4, 62.1, 57.7, 56.9, 47.2, 43.4, 34.7, 30.8, 22.7; (ESI) *m*/*z*: [M + H]^+^ Calcd for C_20_H_26_NO_5_ 360.1805; Found 360.1823; FTIR (neat, cm^–1^) 3371, 2923, 1502, 1439, 1276, 1135.
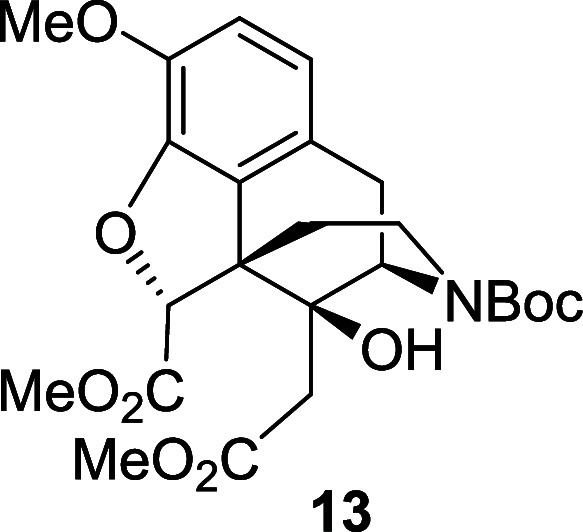


*N*-Boc diol **10** (0.44 g, 1.05 mmol) was dissolved in DCM (2.6 mL) and water (2.6 mL) under air at room temperature. TEMPO (16 mg, 0.104 mmol) and PhI(OAc)_2_ (1.67 g, 5.21 mmol) were added and the solution was stirred for 3 h. The solvent was evaporated and the resulting residue was dissolved in anhydrous toluene (166 mL) and anhydrous methanol (42 mL) under a nitrogen atmosphere. A solution of TMS-diazomethane (2 M in diethyl ether, 3.1 mL, 6.25 mmol) was added at room temperature and the solution was stirred for 3 h. The solvent was removed under reduced pressure. The crude material was purified by flash chromatography on silica gel, eluting with 40% ethyl acetate in petroleum ether to afford the diester **13** (0.2 g, 60%) as a yellow oil, and as a 55:45 mixture of rotamers. ^1^H NMR (400 MHz, CDCl_3_) δ: 6.81 (d, *J* = 8.0 Hz, 1H), 6.68 (d, *J* = 8.0 Hz, 1H), 4.92 (s, 1H), 4.76 (d, *J* = 5.0 Hz, 0.45H minor), 4.62 (d, *J* = 5.5 Hz, 0.55H major), 4.04 (br, 0.55H| major), 3.90 (s, 3H), 3.88 (br, 0.45H minor), 3.77 (br, 3H), 3.71 (br, 3H), 3.11 (br, 1H), 2.87 (br, 1H), 2.82–2.76 (m, 0.45H minor), 2.75–2.70 (m, 0.55H major), 2.70–2.64 (m, 1H), 2.36 (d, *J* = 15.0 Hz, 0.45H minor), 2.35 (d, *J* = 15.0 Hz, 0.55H major), 2.22 (d, *J* = 15.0 Hz, 0.55H major), 2.19 (d, *J* = 15.0 Hz, 0.45H minor), 1.47 (br, 10H); ^13^C{^1^H} NMR (101 MHz, CDCl_3_) δ: 172.2, 170.7, 145.7, 142.8, 127.6, 125.1, 119.9, 114.8, 89.5, 80.2, 73.8, 56.8, 55.1, 54.1, 52.4, 52.2, 38.1, 37.7, 36.4, 33.8, 31.9, 28.5; HRMS (ESI) *m*/*z*: [M + Na]^+^ Calcd for C_24_H_31_NO_9_Na 500.1897; Found 500.1905; FTIR (neat, cm^–1^) 3507, 2959, 1692, 1437, 1284, 1163, 749.
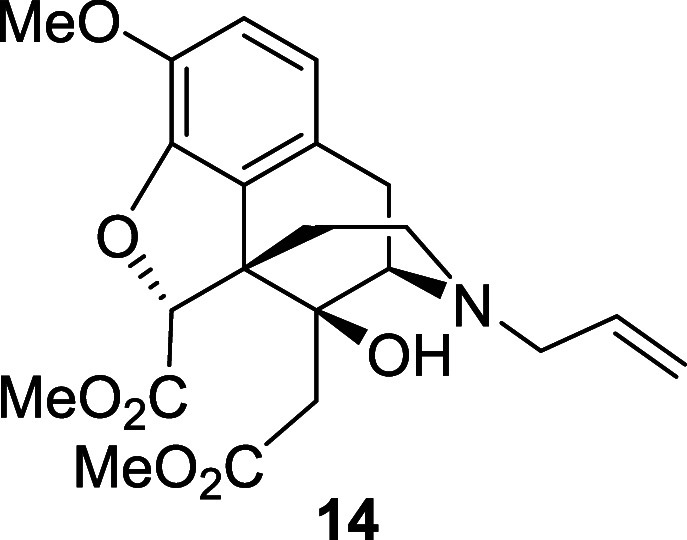


*N*-Boc diester **13** (103 mg, 0.22 mmol) was dissolved in DCM (2 mL) and TFA (2 mL) was added. The reaction mixture was stirred at room temperature for 0.5 h, and the solvent was evaporated. The residue was dissolved in acetone (2 mL). Triethylamine (0.09 mL, 0.65 mmol) and allyl bromide (0.04 mL, 0.43 mol) were added and the reaction mixture was stirred at 50 °C for 20 h. The reaction mixture was allowed to cool to room temperature, an aqueous solution of ammonia was added, and the reaction mixture was stirred for 1 h. The mixture was poured into water and the crude product was extracted with ethyl acetate. The combined organic extracts were washed with brine, dried over magnesium sulfate and the solvent was removed under reduced pressure. The crude material was purified by flash chromatography on silica gel eluting with 100% ethyl acetate to afford the *N*-allyl diester **14** (36 mg, 40%) as a brown oil. ^1^H NMR (400 MHz, CDCl_3_) δ: 6.77 (d, *J* = 8.0 Hz, 1H), 6.67 (d, *J* = 8.0 Hz, 1H), 5.77 (ddt, *J* = 16.5, 10.0, 6.5 Hz, 1H), 5.21–5.13 (m, 2H), 4.90 (s, 1H), 3.89 (s, 3H), 3.82 (s, 3H), 3.63 (s, 3H), 3.45 (d, *J* = 6.5 Hz, 1H), 3.16–3.03 (m, 2H), 3.03 (d, *J* = 18.5 Hz, 1H), 2.84 (dd, *J* = 18.5, 6.5 Hz, 1H), 2.58–2.45 (m, 2H), 2.49 (d, *J* = 14.0 Hz, 1H), 2.18–2.10 (m, 1H), 2.07 (d, *J* = 14.0 Hz, 1H), 1.67–1.60 (m, 1H); ^13^C{^1^H} NMR (101 MHz, CDCl_3_) δ: 171.0, 170.5, 145.5, 142.5, 135.1, 128.5, 126.1, 119.5, 118.1, 114.4, 89.3, 72.8, 61.5, 57.8, 56.7, 54.6, 52.6, 51.8, 42.9, 37.7, 36.1, 22.8; HRMS (ESI) *m*/*z*: [M + H]^+^ Calcd for C_22_H_28_NO_7_ 418.1866; Found 418.1861; FTIR (neat, cm^–1^) 3395, 2951, 2837, 1737, 1506, 1438, 1280, 1196, 1053.
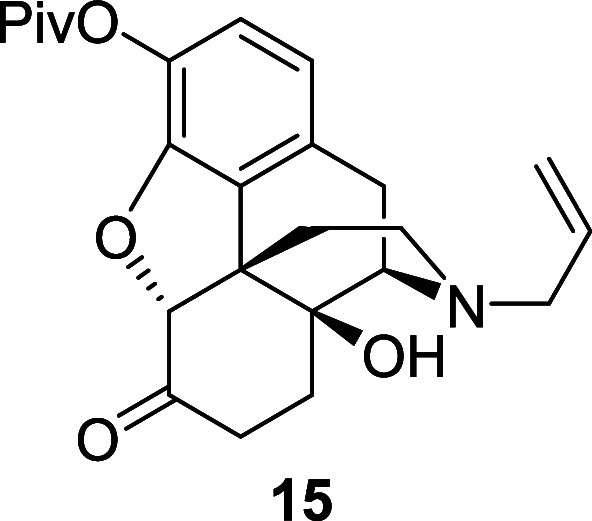


Naloxone (7.37 g, 22.5 mmol) was dissolved in THF (28 mL). Et_3_N (6.3 mL, 45 mmol) and PivCl (4.2 mL, 33.8 mmol) were added and the reaction mixture was stirred at reflux for 45 min. The reaction mixture was allowed to cool to room temperature and the solvent was evaporated. The residue was dissolved in ethyl acetate and filtered through a pad of silica. The solvent was evaporated. The solid was recrystallized from ethanol to afford the naloxone derivative **15** (6.47 g, 70%) as a colorless solid. mp 173–175 °C; ^1^H NMR (400 MHz, CDCl_3_) δ: 6.80 (d, *J* = 8.0 Hz, 1H), 6.67 (d, *J* = 8.0 Hz, 1H), 5.81 (ddt, *J* = 16.5, 10.0, 6.5 Hz, 1H), 5.26–5.14 (m, 2H), 4.65 (s, 1H), 3.17–3.14 (m, 2H), 3.12 (d, *J* = 19.0 Hz, 1H), 3.01 (d, *J* = 6.0 Hz, 1H), 2.98 (td, *J* = 14.5, 5.0 Hz, 1H), 2.60 (dd, *J* = 19.0, 6.0 Hz, 1H), 2.61–2.57 (m, 1H), 2.37 (td, *J* = 12.5, 5.0 Hz, 1H), 2.28 (dt, *J* = 14.5, 3.5 Hz, 1H), 2.14 (td, *J* = 12.5, 3.5 Hz, 1H), 1.85 (ddd, *J* = 14.5, 5.0, 3.5 Hz, 1H), 1.63–1.58 (m, 1H), 1.66–1.54 (m, 1H), 1.37); ^13^C{^1^H} NMR (101 MHz, CDCl_3_) δ: 207.5, 176.3, 148.0, 135.2, 133.1, 130.2, 130.0, 122.9, 119.3, 118.3, 90.5, 70.3, 62.2, 57.8, 50.6, 43.3, 39.2, 36.2, 31.2, 30.7, 27.3, 23.1; HRMS (ESI) *m*/*z*: [M + H]^+^ Calcd for C_24_H_30_NO_5_ 412.2124; Found 412.2124; FTIR (neat, cm^–1^) 3398, 2972, 2932, 1754, 1728, 1443, 1111.
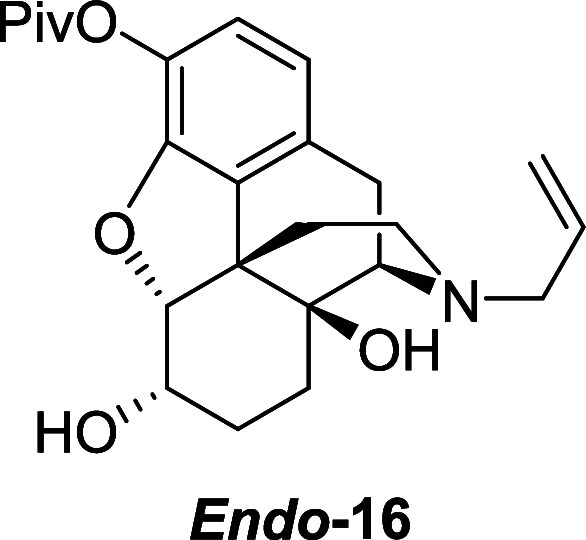


Ketone **15** (6.3 g, 15.3 mmol) was dissolved in acetic acid (51 mL). Sodium (triacetoxy)borohydride (9.0 g, 46 mmol) was added portionwise at room temperature and the reaction mixture was stirred under nitrogen atmosphere for 2 h. Acetone (3.4 mL, 46 mmol) was added and the mixture was stirred for 0.5 h. The pH of the solution was adjusted to 9–10 using an aqueous solution of potassium hydroxide, and the crude product was extracted with DCM. The combined organic extracts were washed with brine, dried over magnesium sulfate and the solvent was removed under reduced pressure to afford the alcohol *endo***–16** (6.05 g, 95%) as a colorless amorphous solid. ^1^H NMR (400 MHz, CDCl_3_) δ: 6.75 (d, *J* = 8.0 Hz, 1H), 6.63 (d, *J* = 8.0 Hz, 1H), 5.79 (ddt, *J* = 16.5, 10.0, 6.5 Hz, 1H), 5.17 (m, 2H), 4.64 (d, *J* = 5.0 Hz, 1H), 4.20–4.13 (m, 1H), 3.14–3.09 (m, 2H), 3.08 (d, *J* = 18.5 Hz, 1H), 2.91 (d, *J* = 6.0 Hz, 1H), 2.64 (dd, *J* = 18.5, 6.0 Hz, 1H), 2.54 (dd, *J* = 11.5, 3.5 Hz, 1H), 2.27–2.12 (m, 2H), 1.95–1.85 (m, 1H), 1.67–1.58 (m, 1H), 1.53–1.42 (m, 3H), 1.35 (s, 9H); ^13^C{^1^H} NMR (101 MHz, CDCl_3_) δ: 176.6, 148.8, 135.4, 133.2, 131.9, 130.8, 121.7, 118.9, 117.9, 91.7, 70.3, 66.7, 62.7, 57.9, 46.3, 43.4, 39.2, 32.1, 27.3, 26.5, 24.1, 23.3; HRMS (ESI) *m*/*z*: [M + H]^+^ Calcd for C_24_H_32_NO_5_ 414.2280; Found 414.2281; FTIR (neat, cm^–1^) 3511, 3408, 2962, 2927, 1741, 1447, 1115, 925, 732.
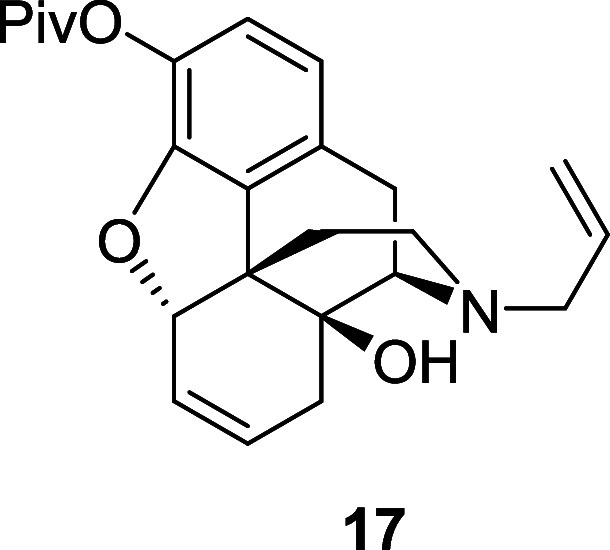


Alcohol *endo***–16** (1.0 g, 2.42 mmol) was dissolved in anhydrous chloroform (24 mL) under a nitrogen atmosphere. 4-Methylmorpholine (1.1 mL, 9.68 mmol) was added and the solution was cooled to −30 °C. Trifluoromethanesulfonic anhydride (0.8 mL, 4.84 mmol) was added dropwise and the reaction mixture was stirred from −30 to 0 °C for 4 h. The solution was allowed to warm to room temperature and washed with saturated aqueous sodium bicarbonate, water and brine. The organic layer was dried over magnesium sulfate and the solvent was removed under reduced pressure. The crude material was purified by flash chromatography on silica gel, eluting with a gradient 20% petroleum ether in DCM to 100% DCM to afford the corresponding triflate (0.77 g, 59%) as a colorless amorphous solid.

A solution of triflate (0.77 g, 1.4 mmol) in anhydrous acetonitrile (24 mL) under a nitrogen atmosphere was cooled to −10 °C. Tetraethylammonium iodide (0.73 g, 2.84 mmol) was added in one portion. The reaction mixture was stirred at −10 °C for 1 h, allowed to warm to room temperature and stirred for 24 h. The solvent was removed under reduced pressure. The residue was dissolved in DCM (10 mL), the organic layer was washed with water and brine, dried over magnesium sulfate and the solvent was removed under reduced pressure to afford the 6-iodo derivative (0.6 g, 82%) as a colorless amorphous solid.

The iodide (7.4 g, 14.22 mmol) was dissolved in anhydrous DMF (71 mL) under a nitrogen atmosphere. DBU (38 mL, 256 mmol) was added and the reaction mixture was stirred at 100 °C for 18 h. After cooling to room temperature, the reaction mixture was poured into a saturated solution of sodium bicarbonate and the crude product was extracted with diethyl ether. The combined organic extracts were washed with brine, dried over magnesium sulfate and the solvent was removed under reduced pressure. The crude material was purified by flash chromatography on silica gel eluting with 20% ethyl acetate in petroleum ether to afford the phenol **18** (1.4 g, 31%) as a yellow amorphous solid, and the desired alkene **17** (1.7 g, 31%) as a pale-yellow oil. Compound **17**: ^1^H NMR (400 MHz, CDCl_3_) δ: 6.76 (d, *J* = 8.0 Hz, 1H), 6.62 (d, *J* = 8.0 Hz, 1H), 5.87–5.83 (m, 1H), 5.81 (ddt, *J* = 16.5, 10.0, 6.5 Hz, 1H), 5.71 (ddd, *J* = 10.5, 6.0, 1.0 Hz, 1H), 5.24–5.13 (m, 2H), 5.03–5.00 (m, 1H), 3.12 (d, *J* = 19.0 Hz, 1H), 3.14–3.10 (m, 2H), 2.95 (d, *J* = 6.5 Hz, 1H), 2.67 (dd, *J* = 19.0, 6.5 Hz, 1H), 2.60–2.55 (m, 1H), 2.25–2.20 (m, 2H), 2.04–1.97 (m, 2H), 1.72–1.62 (m, 1H), 1.34 (s, 9H); ^13^C{^1^H} NMR (101 MHz, CDCl_3_) δ: 176.1, 147.9, 135.3, 133.5, 132.4, 130.8, 129.7, 124.1, 122.2, 118.3, 118.0, 87.7, 70.6, 61.8, 58.0, 45.1, 43.5, 39.1, 31.8, 30.9, 27.2, 23.3; HRMS (ESI) *m*/*z*: [M + H]^+^ Calcd for C_24_H_30_NO_4_ 396.2175; Found 396.2164; FTIR (neat, cm^–1^) 3416, 2969, 2919, 2822, 1753, 1447, 1156, 1110, 906. Compound **18**: ^1^H NMR (400 MHz, CDCl_3_) δ: 6.68 (d, *J* = 8.0 Hz, 1H), 6.56 (d, *J* = 8.0 Hz, 1H), 5.89–5.85 (m, 1H), 5.82 (ddt, *J* = 16.5, 10.0, 6.5 Hz, 1H), 5.72 (ddd, *J* = 10.5, 6.5, 1.5 Hz, 1H), 5.25–5.13 (m, 2H), 5.06–4.99 (m, 1H), 4.82 (br s), 3.15–3.11 (m, 2H), 3.10 (d, *J* = 18.0 Hz, 1H), 2.95 (d, *J* = 5.5 Hz, 1H), 2.64 (dd, *J* = 18.0, 5.5 Hz, 1H), 2.60–2.56 (m, 1H), 2.30–2.18 (m, 2H), 2.08–1.93 (m, 2H), 1.69–1.56 (m, 1H); ^13^C{^1^H} NMR (101 MHz, CDCl_3_) δ: 143.3, 139.2, 135.5, 131.5, 130.2, 125.5, 124.1, 118.8, 118.1, 116.7, 87.9, 70.9, 62.1, 58.1, 45.6, 43.7, 32.0, 31.1, 23.1; HRMS (ESI) *m*/*z*: [M + H]^+^ Calcd for C_19_H_22_NO_3_ 312.1594; Found 312.1607; FTIR (neat, cm^–1^) 3180, 2927, 1610, 1458, 1240, 905.

### Conversion of **18** to **17**

Phenol **18** (1.3 g, 4.18 mmol) was dissolved in THF (11 mL). Et_3_N (1.16 mL, 8.36 mmol) and PivCl (0.78 mL, 6.27 mmol) were added and the reaction mixture was stirred at reflux for 2 h. The reaction mixture was allowed to cool to room temperature and the solvent was evaporated. The residue was dissolved in ethyl acetate and filtered through a pad of silica. The solvent was evaporated and the crude material was purified by flash chromatography on silica gel eluting with 20% ethyl acetate in petroleum ether to afford the alkene **17** (1.18 g, 71%) as a pale-yellow oil.
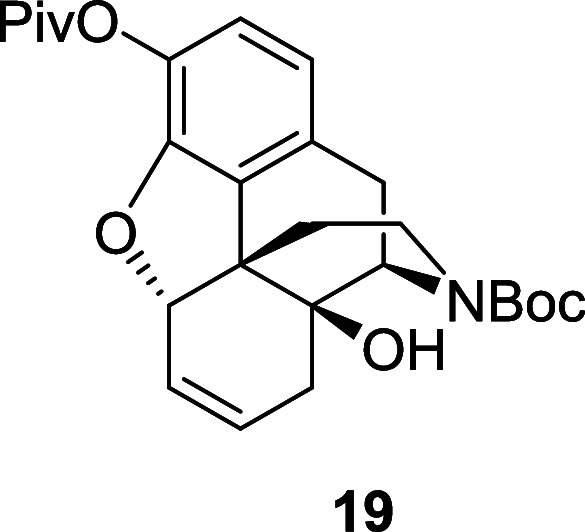


A solution *N*-allyl alkene **17** (1.5 g, 3.78 mmol) in anhydrous DCM (18 mL) was added to a solution of Pd(PPh_3_)_4_ (0.22 g, 0.19 mmol) and *N*,*N*-dimethylbarbituric acid (0.89 g, 5.67 mmol) in anhydrous DCM (20 mL) under a nitrogen atmosphere. The solution was stirred at 40 °C overnight. After completion, diisopropylethylamine (0.07 mL, 0.38 mmol) and Boc_2_O (1.74 mL, 7.56 mmol) were added, the temperature was increased to 60 °C and the reaction mixture was stirred for 7 h. The reaction mixture was allowed to cool to room temperature, poured into water and the crude product was extracted with DCM. The combined organic layers were washed with brine, dried over magnesium sulfate and the solvent was removed under reduced pressure. The crude material was purified by flash chromatography on silica gel eluting with 20% to 50% ethyl acetate in petroleum ether to afford the *N*-Boc alkene **19** (1.58 g, 92%) as a yellow oil, and as a 70:30 mixture of rotamers. ^1^H NMR (400 MHz, CDCl_3_) δ: 6.80 (d, *J* = 8.0 Hz, 1H), 6.64 (d, *J* = 8.0 Hz, 1H), 5.82 (ddd, *J* = 10.5, 4.5, 3.0 Hz, 1H), 5.76–5.70 (m, 1H), 5.02–4.98 (m, 1H), 4.51 (br, 0.7H major), 4.34 (br, 0.3H minor), 3.92 (br, 1H), 3.18 (dd, *J* = 18.5, 6.5 Hz, 1H), 2.93 (d, *J* = 18.5 Hz, 1H), 2.86 (br, 1H), 2.53 (br, 1H), 2.27 (br, 1H), 2.07–2.02 (m, 2H), 1.64 (br, 1H), 1.48 (s, 9H), 1.34 (s, 9H); ^13^C{^1^H} NMR (101 MHz, CDCl_3_) δ: 176.2, 148.0, 133.9, 131.7, 130.1, 129.3, 124.3, 122.8, 120.2, 118.7, 87.4, 80.6, 71.6, 55.3, 45.2, 39.2, 38.0, 32.6, 29.1, 28.5, 27.3; HRMS (ESI) *m*/*z*: [M + Na]^+^ Calcd for C_26_H_33_NO_6_Na 478.2206; Found 478.2223; FTIR (neat, cm^–1^) 3442, 2974, 1686, 1421, 1157, 1111.
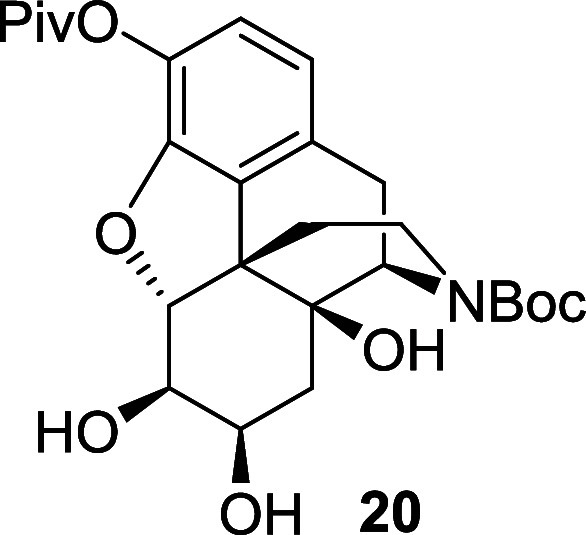


*N*-Boc alkene **19** (0.93 g, 2.01 mmol) was dissolved in ethyl acetate (13 mL), acetonitrile (13 mL) and water (4 mL) under air and the solution was cooled to 0 °C. RuCl_3_.H_2_O (72 mg, 0.286 mmol) and sodium periodate (0.66 g, 3.06 mmol) were successively added and the reaction mixture was stirred from 0 °C to room temperature for 3 h. An aqueous solution of sodium thiosulfate was added, the mixture was stirred for 15 min. The layers were separated and the crude product was extracted with DCM. The combined organic layers were washed with brine, dried over magnesium sulfate and the solvent was removed under reduced pressure. The crude material was purified by flash chromatography on silica gel eluting with 40% ethyl acetate in petroleum ether followed by 100% ethyl acetate, to afford the diol **20** (0.59 g, 59%) as a brown oil. The ^1^H NMR showed significant line broadening due to slowly interconverting rotamers. ^1^H NMR (400 MHz, CDCl_3_) δ: 6.78 (d, *J* = 8.0 Hz, 1H), 6.65 (d, *J* = 8.0 Hz, 1H), 4.59 (d, *J* = 6.0 Hz, 1H), 4.52 (br, 1H), 3.99 (br, 1H), 3.87 (br, 1H), 3.44 (br, 1H), 3.03 (dd, *J* = 18.5, 5.5 Hz, 1H), 2.87 (d, *J* = 18.5 Hz, 1H), 2.67 (br, 1H), 2.38 (td, *J* = 12.5, 5.5 Hz, 1H), 2.01 (dd, *J* = 14.5, 4.0 Hz, 1H), 1.54 (dd, *J* = 14.5, 3.0 Hz, 1H), 1.50–1.40 (m, 10H), 1.33 (s, 9H); ^13^C{^1^H} NMR (101 MHz, CDCl_3_) δ: 176.4, 156.0, 147.2, 134.2, 132.1, 129.7, 122.9, 119.4, 94.4, 80.2, 73.4, 71.7, 69.9, 55.0, 47.6, 39.1, 37.8, 34.4, 31.8, 28.5, 28.0, 27.2; HRMS (ESI) *m*/*z*: [M + Na]^+^ Calcd for C_26_H_35_NO_8_Na 512.2260; Found 512.2260; FTIR (neat, cm^–1^) 3403, 2974, 1664, 1418, 1111, 734.
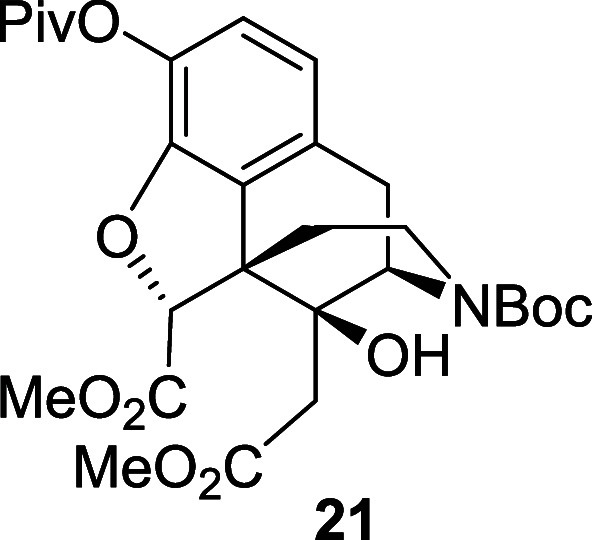


*N*-Boc diol **20** (0.76 g, 1.56 mmol) was dissolved in a 1:1 mixture of DCM and water (7.8 mL total) under air. TEMPO (24 mg, 0.156 mmol) and PhI(OAc)_2_ (2.5 g, 7.83 mmol) were added and the reaction mixture was stirred at room temperature for 3 h. The solvent was removed under vacuum. The residue was dissolved in anhydrous toluene (25 mL) and anhydrous methanol (6 mL, dried over 4 Å molecular sieves for 12 h) under a nitrogen atmosphere. A solution of TMS-diazomethane (2 M in Et_2_O, 4.7 mL, 9.33 mmol) was added and the reaction mixture was stirred at room temperature for 1.5 h. The solvent was removed under vacuum and the crude material was purified by flash chromatography on silica gel eluting with 20% diethyl ether in petroleum ether followed by 20% MeOH in DCM to afford the diester **21** (0.77 g, 90%) as a colorless oil, and as a 53:47 mixture of rotamers. ^1^H NMR (400 MHz, CDCl_3_) δ: 6.89 (d, *J* = 8.0 Hz, 1H), 6.71 (d, *J* = 8.0 Hz, 1H), 4.91 (s, 1H), 4.76 (d, *J* = 5.0 Hz, 0.45H minor), 4.61 (d, *J* = 5.0 Hz, 0.55H major), 4.03 (br, 0.45H minor), 3.86 (br, 0.55H major), 3.75 (s, 3H), 3.71 (s, 3H), 3.13 (dd, *J* = 18.0, 5.0 Hz, 1H), 2.89 (d, *J* = 18.0 Hz, 1H), 2.75 (br, 0.55H major), 2.64 (br, 1.45H major and minor), 2.34 (br, 1H), 2.18 (br, 1H), 1.57–1.50 (m, 1H), 1.46 (s, 9H), 1.35 (s, 9H); ^13^C{^1^H} NMR (101 MHz, CDCl_3_) δ: 176.2, 172.1, 170.3, 170.2, 155.9, 155.3, 148.5, 132.9, 130.4, 130.2, 128.3, 123.6, 119.7, 89.7, 80.2, 73.7, 55.0, 53.9, 53.8, 52.4, 52.3, 39.2, 38.0, 37.5, 36.2, 33.6, 32.2, 28.5, 27.3; HRMS (ESI) *m*/*z*: [M + Na]^+^ Calcd for C_28_H_37_NO_10_Na 570.2315; Found 570.2328; FTIR (neat, cm^–1^) 3496, 2974, 1755, 1693, 1452, 1161, 1111, 749.
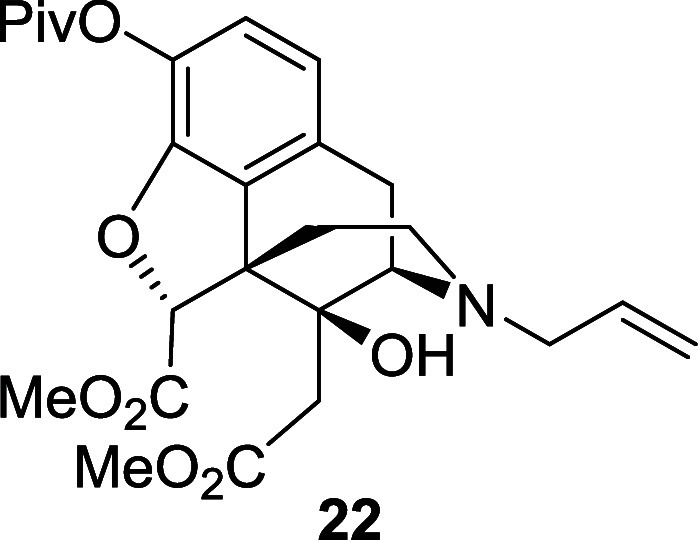


*N*-Boc diester **21** (0.77 g, 1.41 mmol) was dissolved in DCM (14 mL) under air. TFA (1.1 mL, 14.1 mmol) was added and the reaction mixture was stirred at room temperature for 2 h. The solvent was evaporated and the residue dissolved in acetone (14 mL) under air. Et_3_N (1.2 mL, 8.46 mmol) and allyl bromide (0.49 mL, 5.64 mmol) were added and the reaction mixture was heated at 50 °C for 2.5 h. An aqueous solution of ammonia was added, and the solution was stirred for 10 min. The reaction mixture was diluted with water and extracted with ethyl acetate. The combined organic extracts were washed with brine and the solvent was removed under reduced pressure. The crude material was purified by flash chromatography on silica gel eluting with 20% to 50% diethyl ether in petroleum ether to afford the *N*-allyl diester **22** (0.42 g, 61%) as a yellow oil. ^1^H NMR (400 MHz, CDCl_3_) δ: 6.84 (d, *J* = 8.0 Hz, 1H), 6.69 (d, *J* = 8.0 Hz, 1H), 5.75 (ddt, *J* = 16.5, 10.0, 6.5 Hz, 1H), 5.20–5.12 (m, 2H), 4.88 (s, 1H), 4.72 (br, 1H), 3.79 (s, 3H), 3.61 (s, 3H), 3.44 (d, *J* = 6.0 Hz, 1H), 3.10–3.05 (m, 2H), 3.04 (d, *J* = 19.0 Hz, 1H), 2.85 (dd, *J* = 19.0, 6.0 Hz, 1H), 2.58–2.46 (m, 2H), 2.46 (d, *J* = 14.0 Hz, 1H), 2.12 (td, *J* = 11.2, 2.5 Hz, 1H), 2.03 (d, *J* = 14.0 Hz, 1H), 1.65 (dd, *J* = 12.6, 2.5 Hz, 1H), 1.33 (s, 9H); ^13^C{^1^H} NMR (101 MHz, CDCl_3_) δ: 176.3, 170.8, 170.1, 148.4, 134.9, 132.4, 131.2, 129.1, 123.1, 119.3, 118.1, 89.5, 72.7, 61.2, 57.7, 54.4, 52.5, 51.8, 42.7, 39.1, 37.6, 36.0, 27.2, 23.0; HRMS (ESI) *m*/*z*: [M + H]^+^ Calcd for C_26_H_34_NO_8_ 488.2284; Found 488.2287; FTIR (neat, cm^–1^) 3406, 2955, 1751, 1452, 1108.
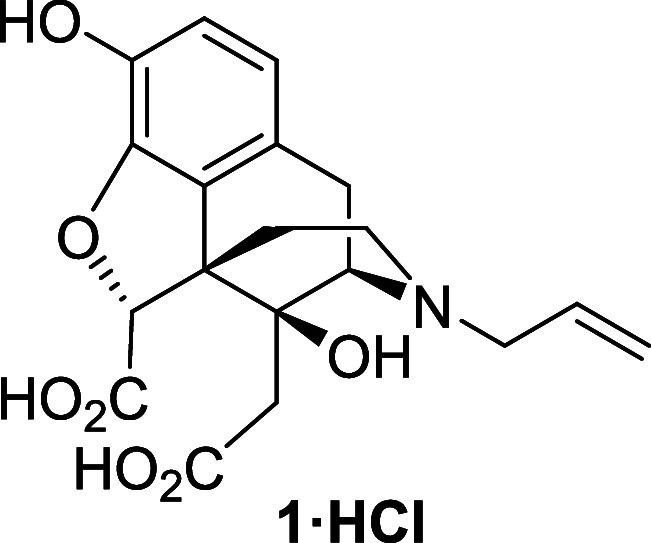


Diester **22** (25.7 mg, 0.053 mmol) was dissolved in concentrated hydrochloric acid (0.53 mL) under air and heated at reflux for 45 min. The solvent was removed under reduced pressure to afford the diacid **1**.HCl (22.3 mg, 100%) as a brown foam. ^1^H NMR (400 MHz, D_2_O) δ: 6.88 (d, *J* = 8.0 Hz, 1H), 6.79 (d, *J* = 8.0 Hz, 1H), 5.90 (ddt, *J* = 16.5, 11.5, 7.0 Hz, 1H), 5.67–5.58 (m, 2H), 5.07 (s, 1H), 4.22 (d, *J* = 7.0 Hz, 1H), 3.90–3.84 (m, 2H), 3.42 (d, *J* = 20.0 Hz, 1H), 3.31–3.25 (m, 1H), 3.15 (dd, *J* = 20.0, 7.0 Hz, 1H), 2.97–2.79 (m, 2H), 2.75 (d, *J* = 15.5 Hz, 1H), 2.19 (d, *J* = 15.5 Hz, 1H), 1.92 (dd, *J* = 14.5, 2.5 Hz, 1H); ^13^C{^1^H} NMR (101 MHz, D_2_O) δ: 174.2, 173.4, 144.1, 138.2, 126.6, 126.4, 125.5, 122.4, 120.6, 118.7, 88.5, 72.9, 60.0, 55.9, 51.7, 45.0, 37.6, 32.8, 22.7; HRMS (ESI) *m*/*z*: [M + H]^+^ Calcd for C_19_H_22_NO_7_ 376.1396; Found 376.1398; FTIR (neat, cm^–1^) 3350, 2949, 2838, 2505, 1648, 1450, 1014.

## Data Availability

The data underlying this study are available in the published article and its Supporting Information.

## Supporting Information Available

Details of NMR spectral data and HPLC chromatograms of Degradant E and compound **1** (PDF)
