# Supplementary material for: The Synthesis of a Naloxone-Related Oxidative Drug Product Degradant
Source: J Org Chem. 2025 Apr 14;90(16):5632–41. doi: 10.1021/acs.joc.5c00313 (PMC12038842; doi:10.1021/acs.joc.5c00313)

Supporting Information

The Synthesis of a Naloxone-Related Oxidative Drug Product Degradant

Marie-Angélique F. S. Deschamps,[a] John S. Carey*,[b] and Joseph P. A. Harrity*,[a]

[a]*Division of Chemistry, School of Mathematical and Physical Sciences, University of Sheffield, Brook Hill, Sheffield, U. K. S3 7HF*

j.harrity@sheffield.ac.uk

[b]*Indivior UK Ltd, Henry Boot Way, Hull, U. K. HU4 7DY*

john.carey@indivior.com

Table of Contents

$^{1}H/^{13}C$ NMR spectra of ***Endo*-4** **S3**

$^{1}H/^{13}C$ NMR spectra of **5** **S4**

$^{1}H/^{13}C$ NMR spectra of **2** **S5**

$^{1}H/^{13}C$ NMR spectra of **7** **S6**

$^{1}H/^{13}C$ NMR spectra of **9** **S7**

$^{1}H/^{13}C$ NMR spectra of **10** **S8**

$^{1}H/^{13}C$ NMR spectra of **11** **S9**

$^{1}H/^{13}C$ NMR spectra of **13** **S10**

$^{1}H/^{13}C$ NMR spectra of **14** **S11**

$^{1}H/^{13}C$ NMR spectra of **15** **S12**

$^{1}H/^{13}C$ NMR spectra of ***Endo*-16** **S13**

$^{1}H/^{13}C$ NMR spectra of **17** **S14**

$^{1}H/^{13}C$ NMR spectra of **19** **S15**

$^{1}H/^{13}C$ NMR spectra of **20** **S16**

$^{1}H/^{13}C$ NMR spectra of **21** **S17**

$^{1}H/^{13}C$ NMR spectra of **22** **S18**

$^{1}H/^{13}C$ NMR spectra of **1·HCl** **S19**

^{1}H NMR spectrum of diacid **1** produced by forced degradation **S20**

HPLC Analysis of Degradant E and Compound **1** **S21**

Structural assignment of compound **6** **S23**

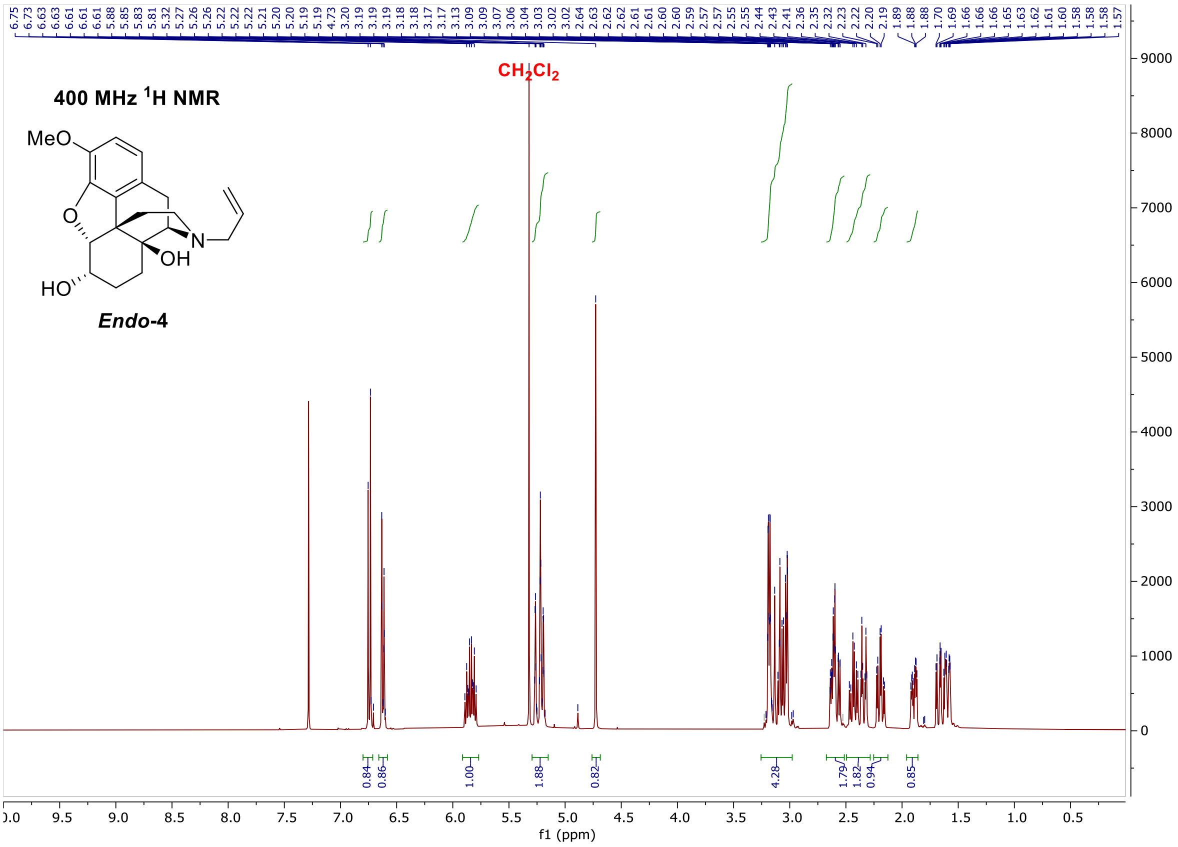
400 MHz ^{1}H NMR
MeO
O
HO
N
OH
Endo-4
CH$_2$Cl$_2$
f1 (ppm)

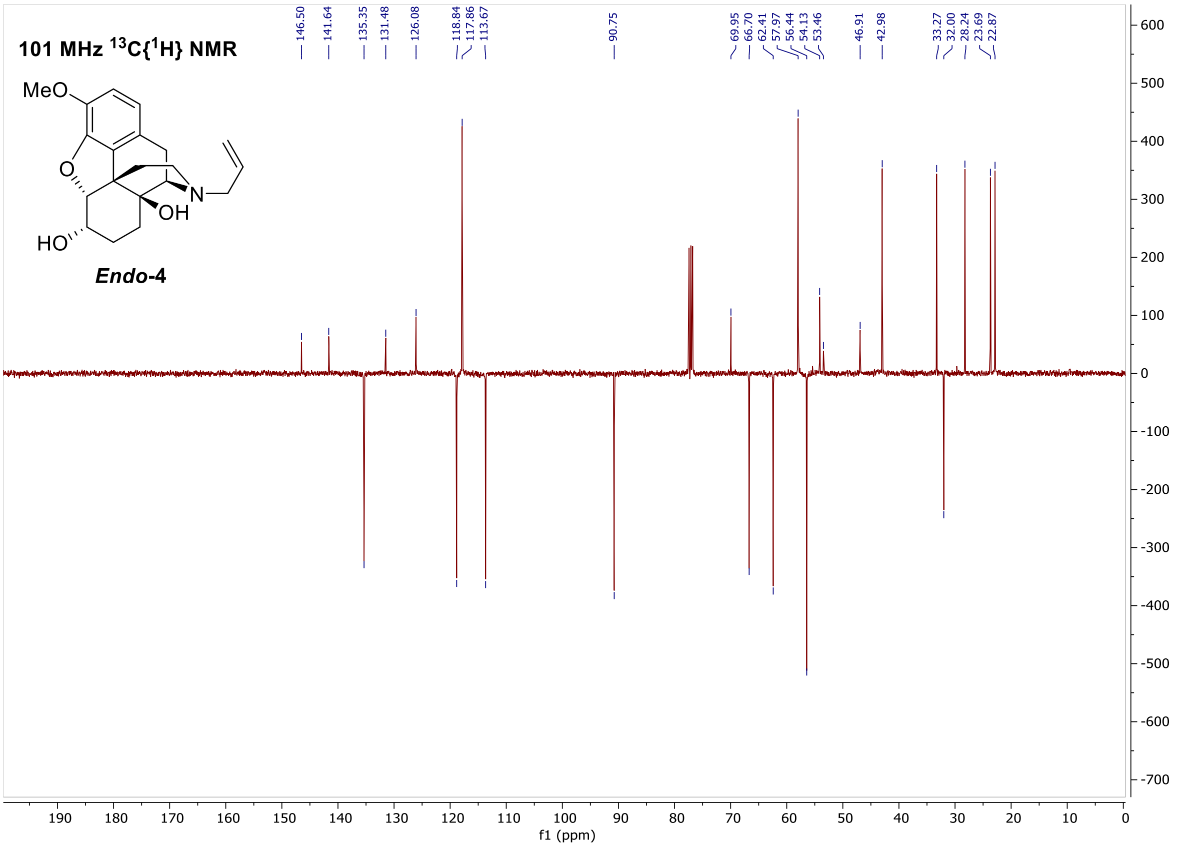
101 MHz ^{13}C{^{1}H} NMR
MeO
O
HO
N
OH
Endo-4
146.50
141.64
135.35
131.48
126.08
118.84
117.86
113.67
90.75
69.95
66.70
62.41
57.97
56.44
54.13
53.46
46.91
42.98
33.27
32.00
28.24
23.69
22.87
f1 (ppm)

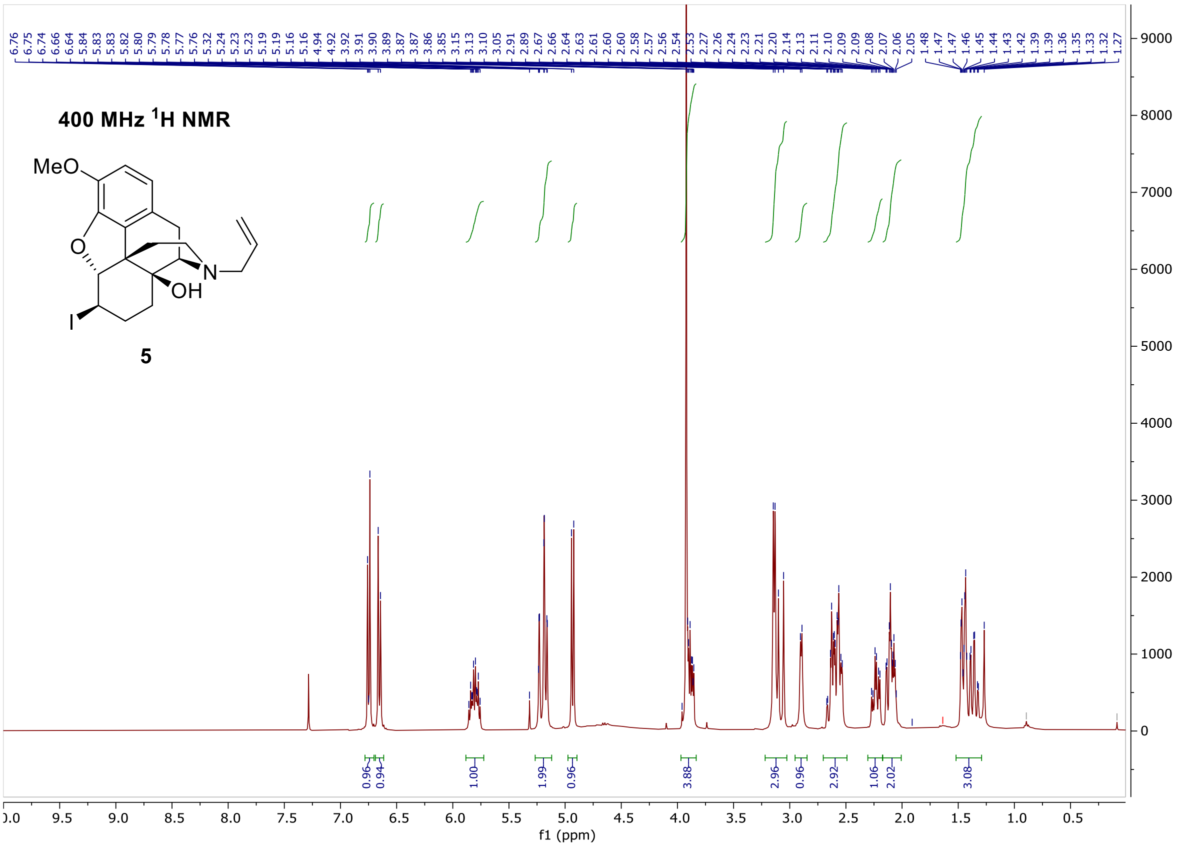
400 MHz ¹H NMR
MeO
O
N
OH
I
5
f1 (ppm)

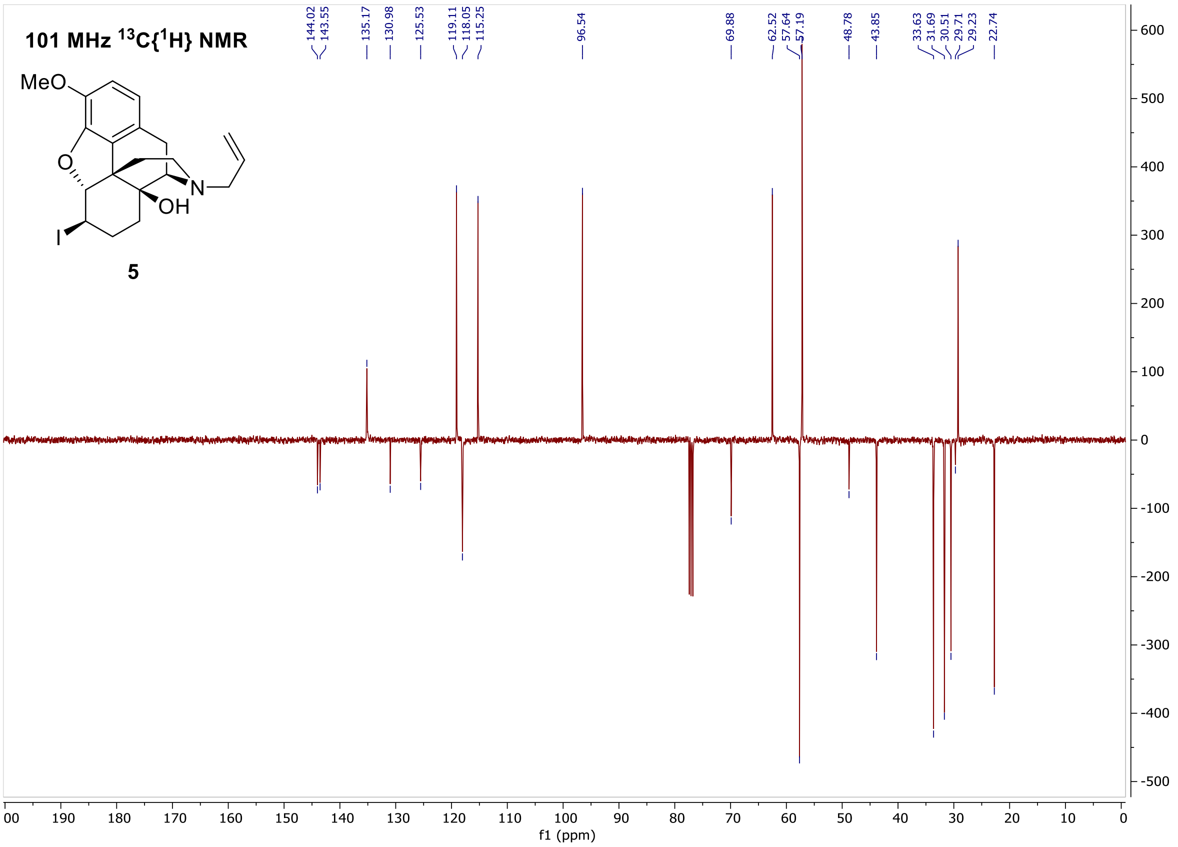
101 MHz ¹³C{¹H} NMR
MeO
O
N
OH
I
5
144.02
143.55
135.17
130.98
125.53
119.11
118.05
115.25
96.54
69.88
62.52
57.64
57.19
48.78
43.85
33.63
31.69
30.51
29.71
29.23
22.74
f1 (ppm)

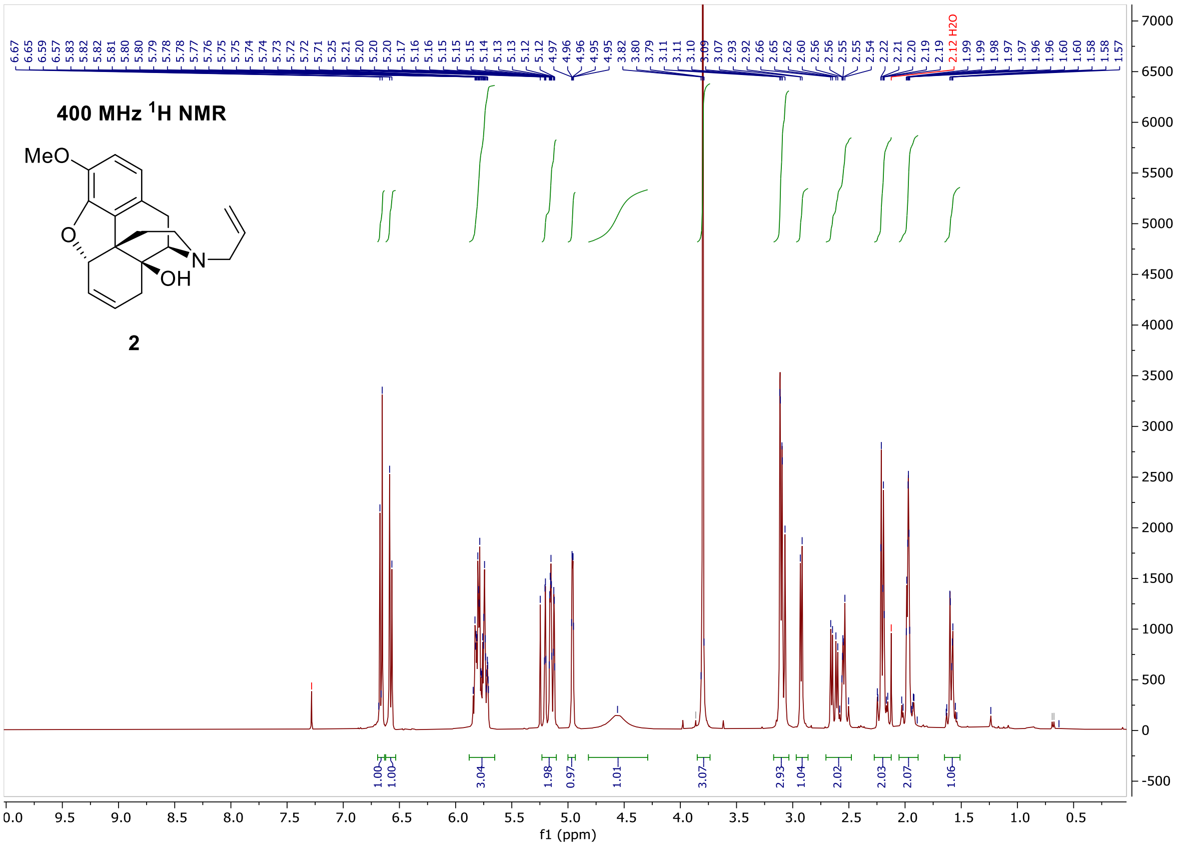
400 MHz 1H NMR
MeO
O
N
OH
2
6.67 6.65 6.59 6.57 5.83 5.82 5.82 5.81 5.80 5.80 5.79 5.78 5.78 5.77 5.76 5.75 5.75 5.74 5.74 5.73 5.72 5.72 5.72 5.71 5.25 5.21 5.20 5.20 5.20 5.17 5.16 5.16 5.15 5.15 5.15 5.14 5.13 5.13 5.12 5.12 4.97 4.96 4.96 4.95 4.95 3.82 3.80 3.79 3.11 3.11 3.10 3.09 3.07 2.93 2.92 2.66 2.65 2.62 2.60 2.56 2.56 2.55 2.55 2.54 2.22 2.21 2.20 2.19 2.19 2.12 H2O 1.99 1.99 1.98 1.97 1.97 1.96 1.96 1.60 1.60 1.58 1.58 1.57
1.00 1.00 3.04 1.98 0.97 1.01 3.07 2.93 1.04 2.02 2.03 2.07 1.06
f1 (ppm)

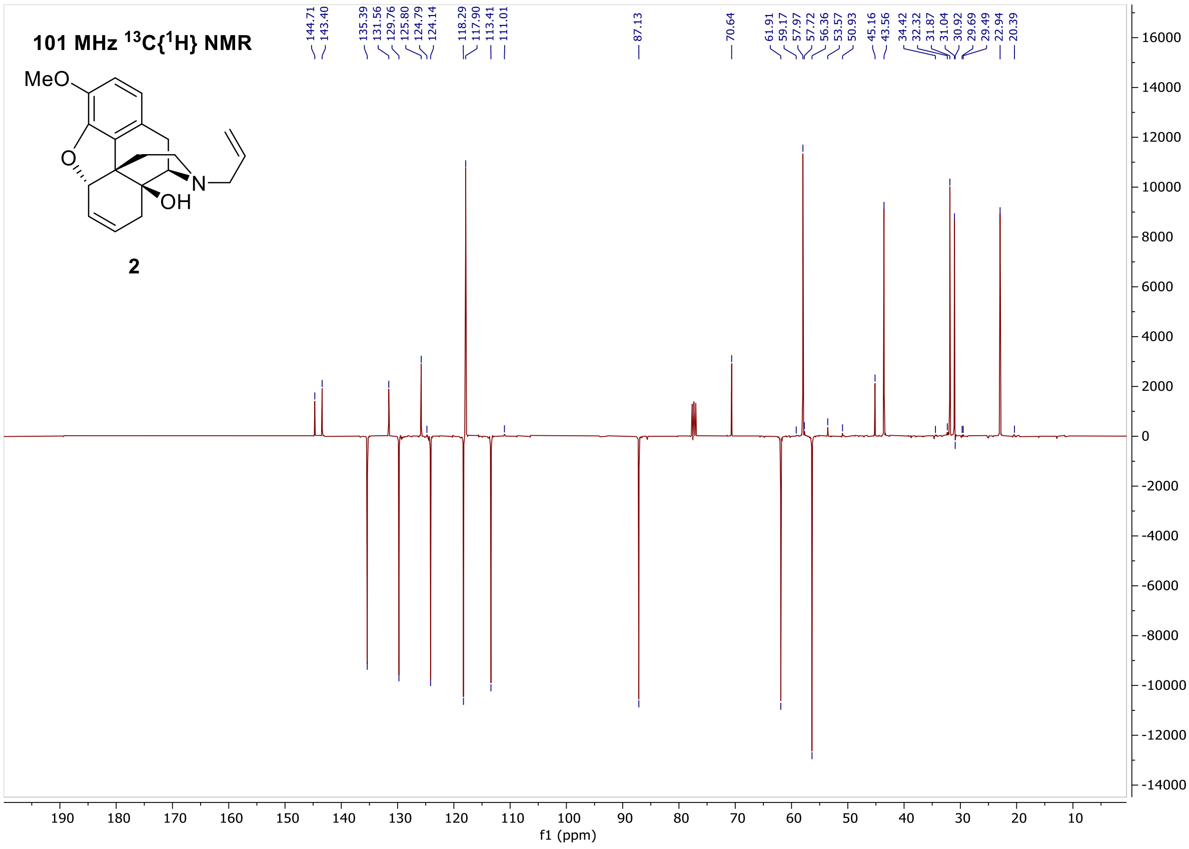
101 MHz 13C{1H} NMR
MeO
O
N
OH
2
144.71 143.40 135.39 131.56 129.76 125.80 124.79 124.14 118.29 117.90 113.41 111.01 87.13 70.64 61.91 59.17 57.97 57.72 56.36 53.57 50.93 45.16 43.56 34.42 32.32 31.87 31.04 30.92 29.69 29.49 22.94 20.39
f1 (ppm)

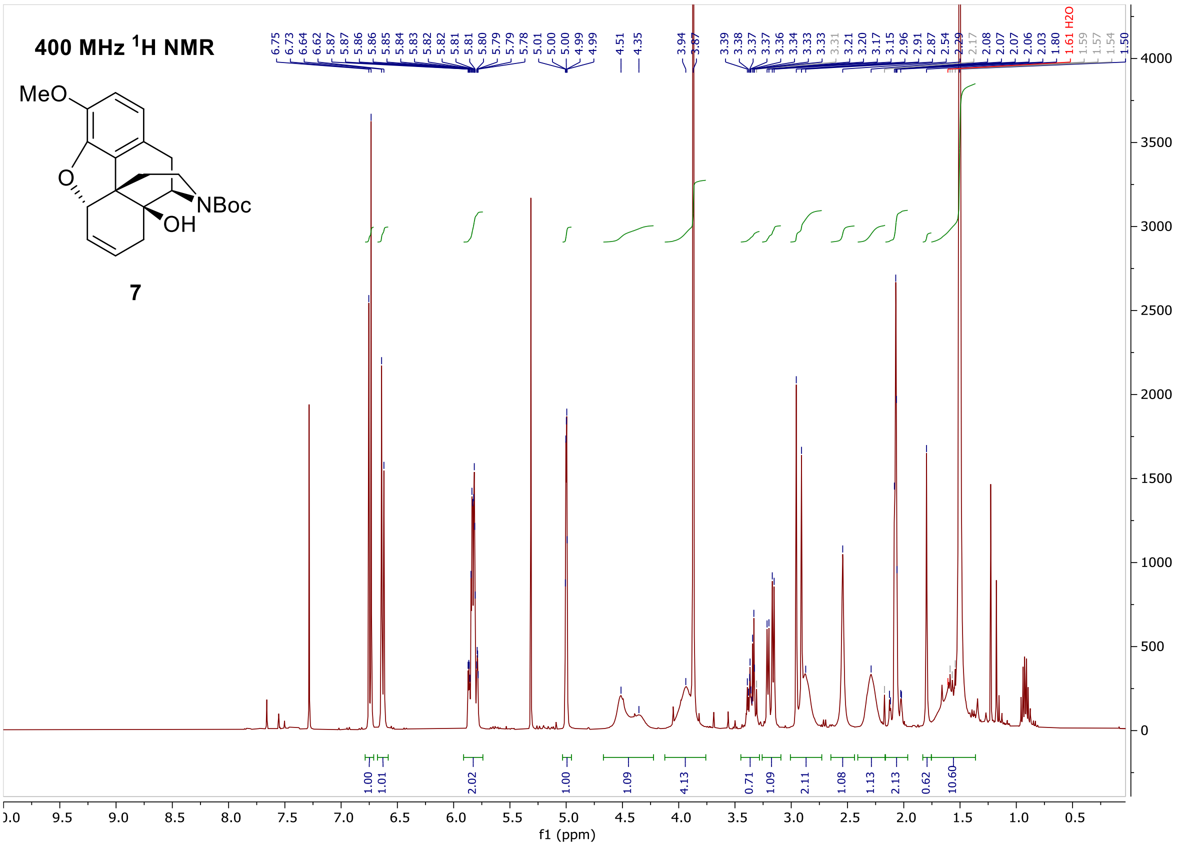
400 MHz ^{1}H NMR
MeO
O
NBoc
OH
7
6.75 6.73 6.64 6.62 5.87 5.87 5.86 5.85 5.84 5.83 5.82 5.82 5.81 5.81 5.80 5.79 5.79 5.78 5.01 5.00 5.00 4.99 4.99 4.51 4.35 3.94 3.87 3.39 3.38 3.37 3.37 3.36 3.34 3.33 3.33 3.31 3.31 3.21 3.20 3.17 3.15 2.96 2.91 2.87 2.54 2.29 2.17 2.08 2.07 2.07 2.06 2.03 1.80 1.61 H2O 1.59 1.57 1.54 1.50
1.00 1.01 2.02 1.00 1.09 4.13 0.71 1.09 2.11 1.08 1.13 2.13 0.62 10.60
f1 (ppm)

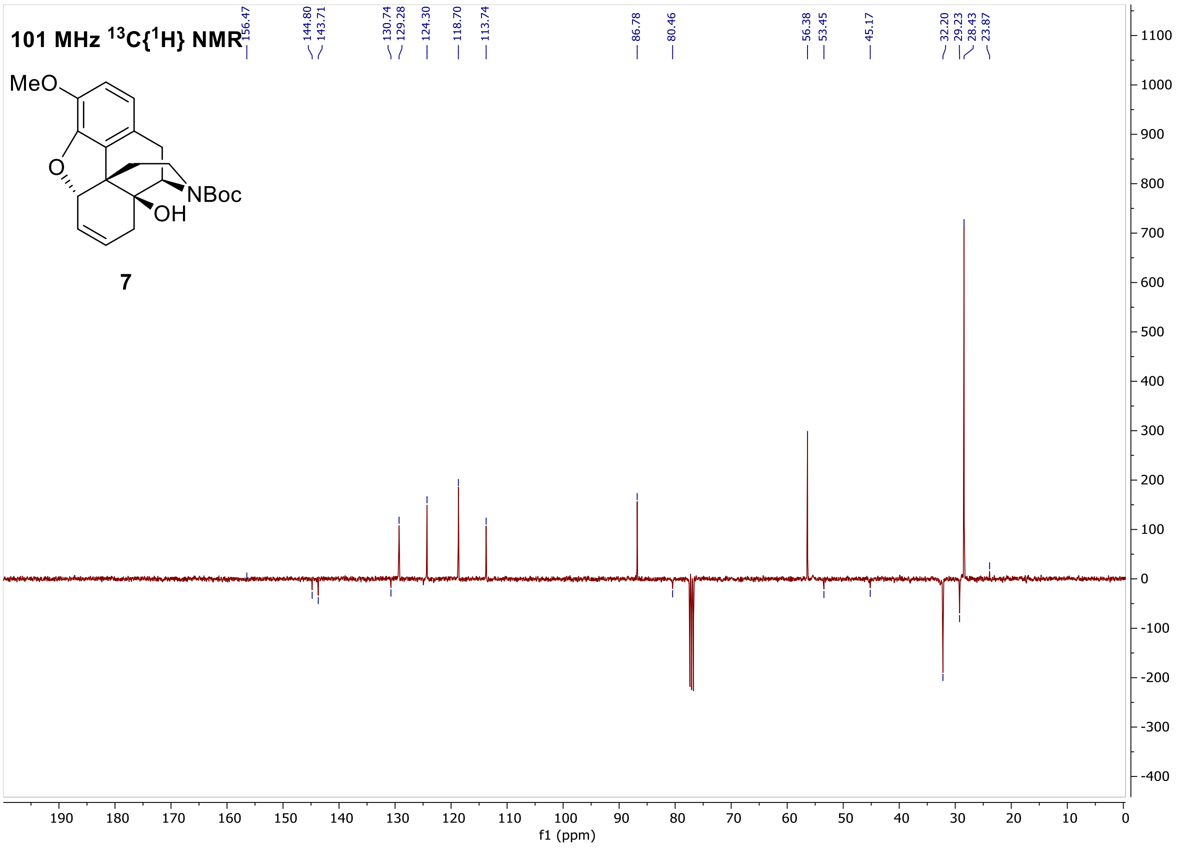
101 MHz ^{13}C{^{1}H} NMR
MeO
O
NBoc
OH
7
156.47 144.80 143.71 130.74 129.28 124.30 118.70 113.74 86.78 80.46 56.38 53.45 45.17 32.20 29.23 28.43 23.87
f1 (ppm)

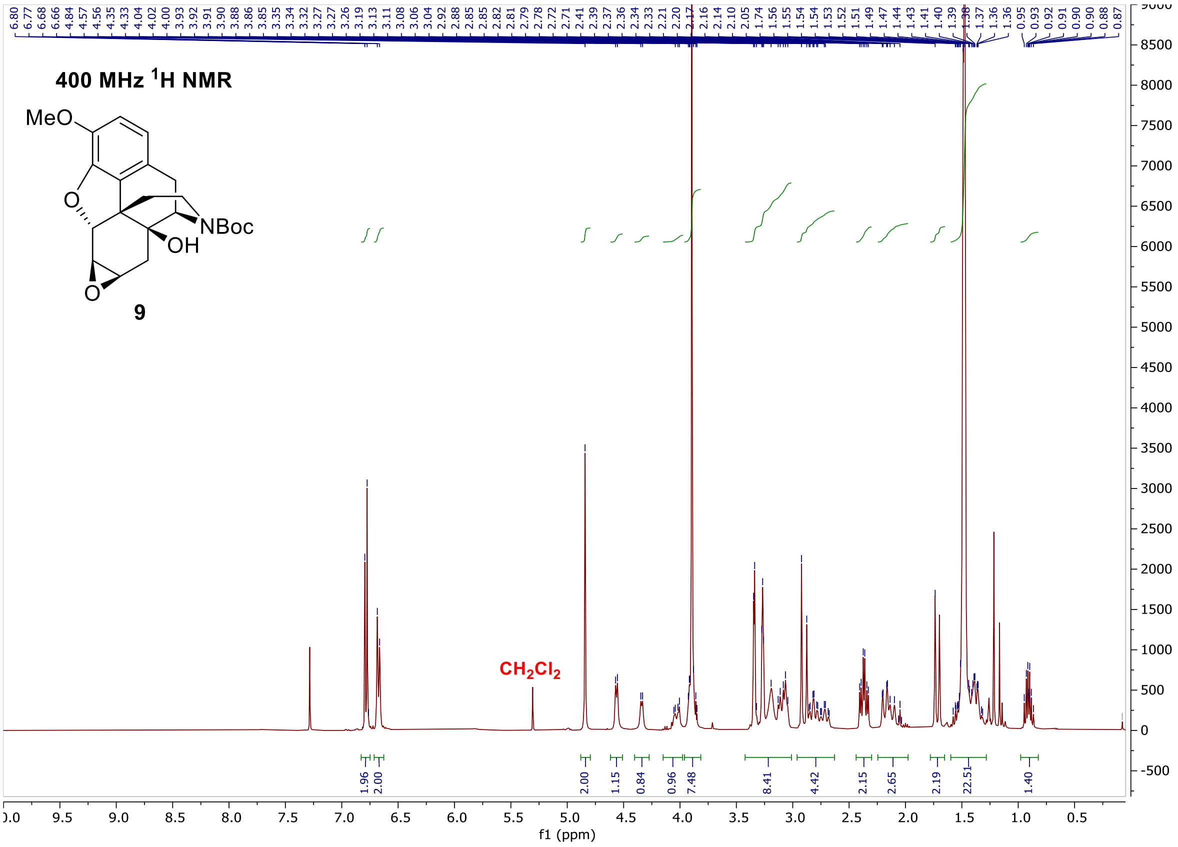
400 MHz ¹H NMR
MeO
O
NBoc
OH
O
9
CH2Cl2
f1 (ppm)

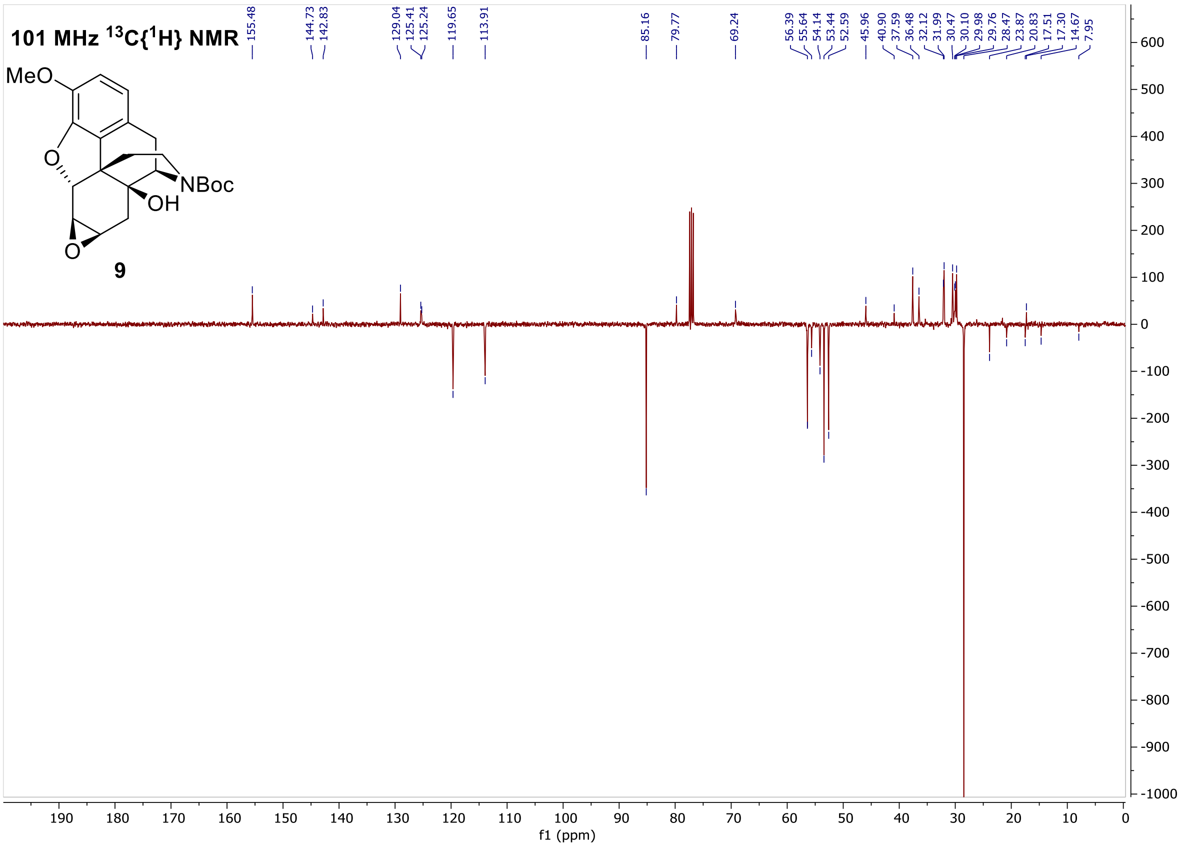
101 MHz ¹³C{¹H} NMR
155.48
144.73
142.83
129.04
125.41
125.24
119.65
113.91
85.16
79.77
69.24
56.39
55.64
54.14
53.44
52.59
45.96
40.90
37.59
36.48
32.12
31.99
30.47
30.10
29.98
29.76
28.47
23.87
20.83
17.51
17.30
14.67
7.95
MeO
O
NBoc
OH
O
9
f1 (ppm)

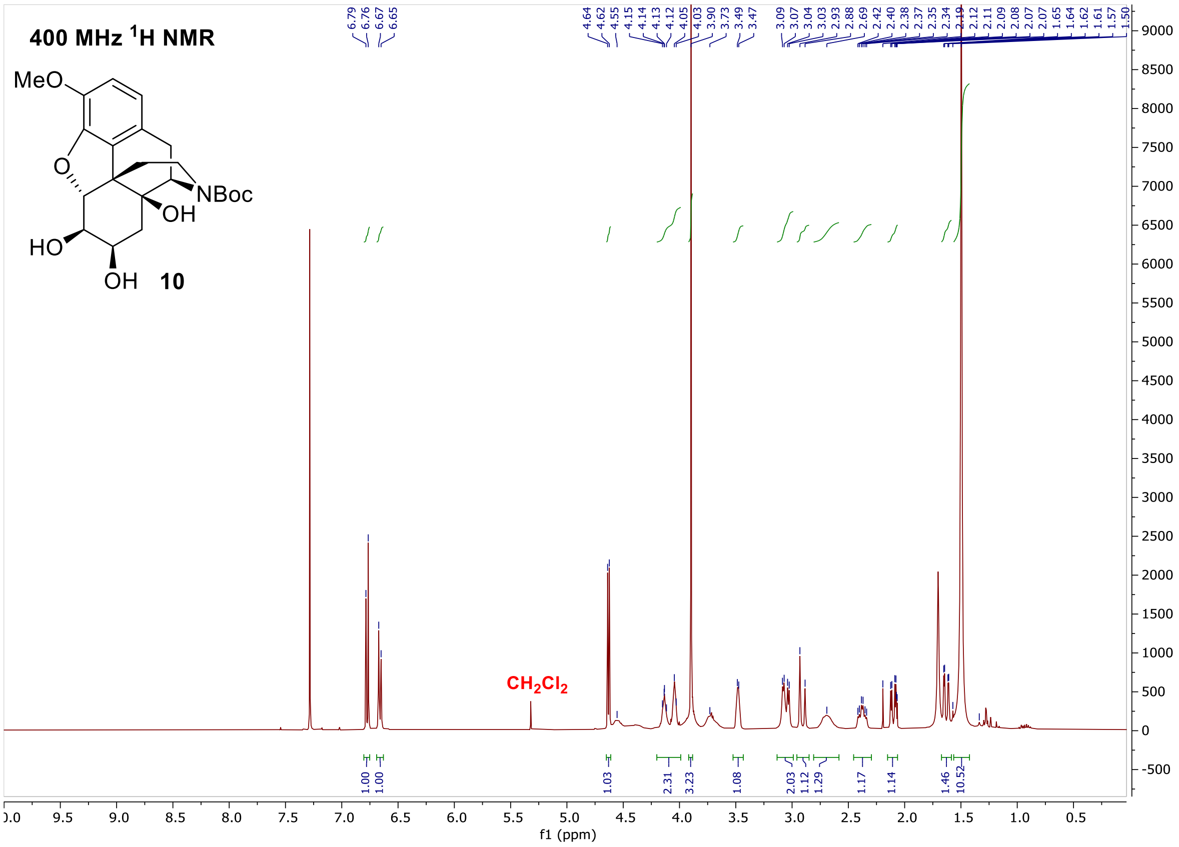
400 MHz ^{1}H NMR
MeO
O
NBoc
OH
HO
OH
10
CH$_2$Cl$_2$
f1 (ppm)

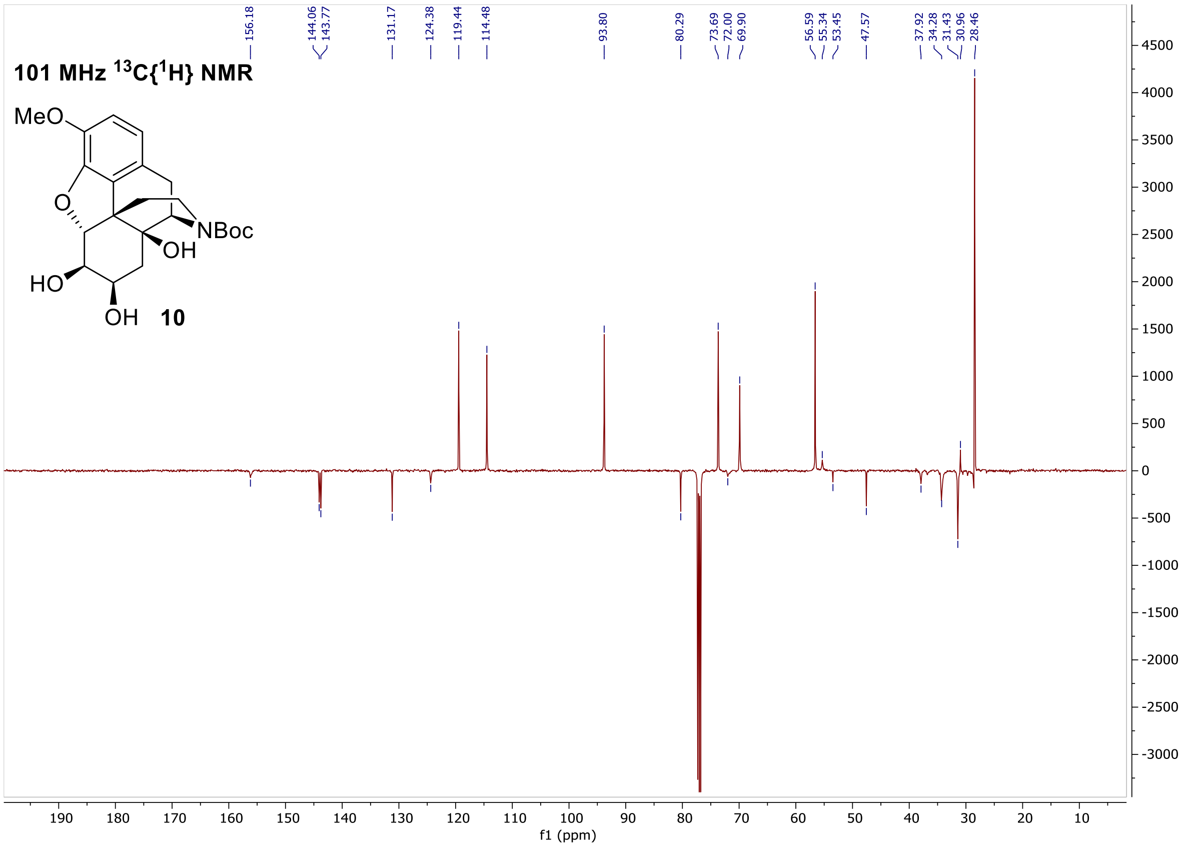
101 MHz ^{13}C{^{1}H} NMR
MeO
O
NBoc
OH
HO
OH
10
f1 (ppm)

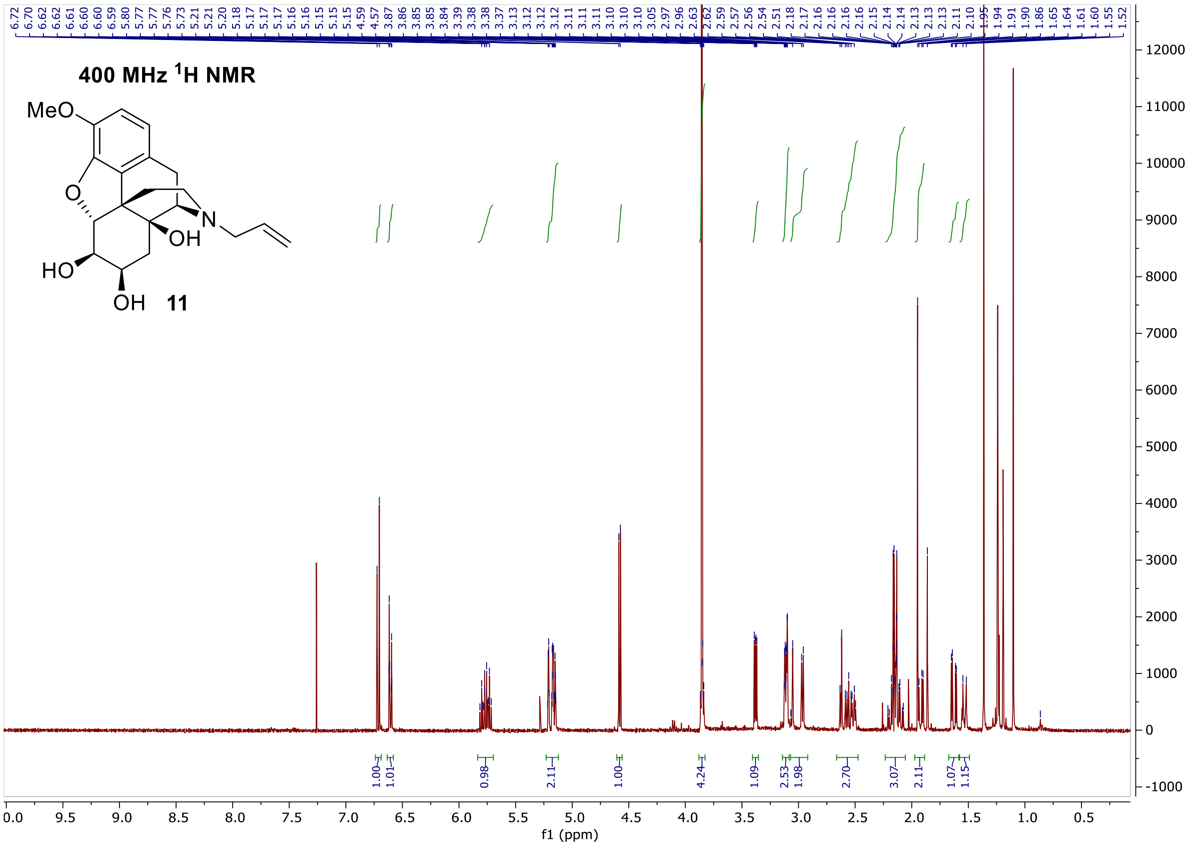
400 MHz ¹H NMR
MeO
O
N
OH
HO
OH
11
f1 (ppm)

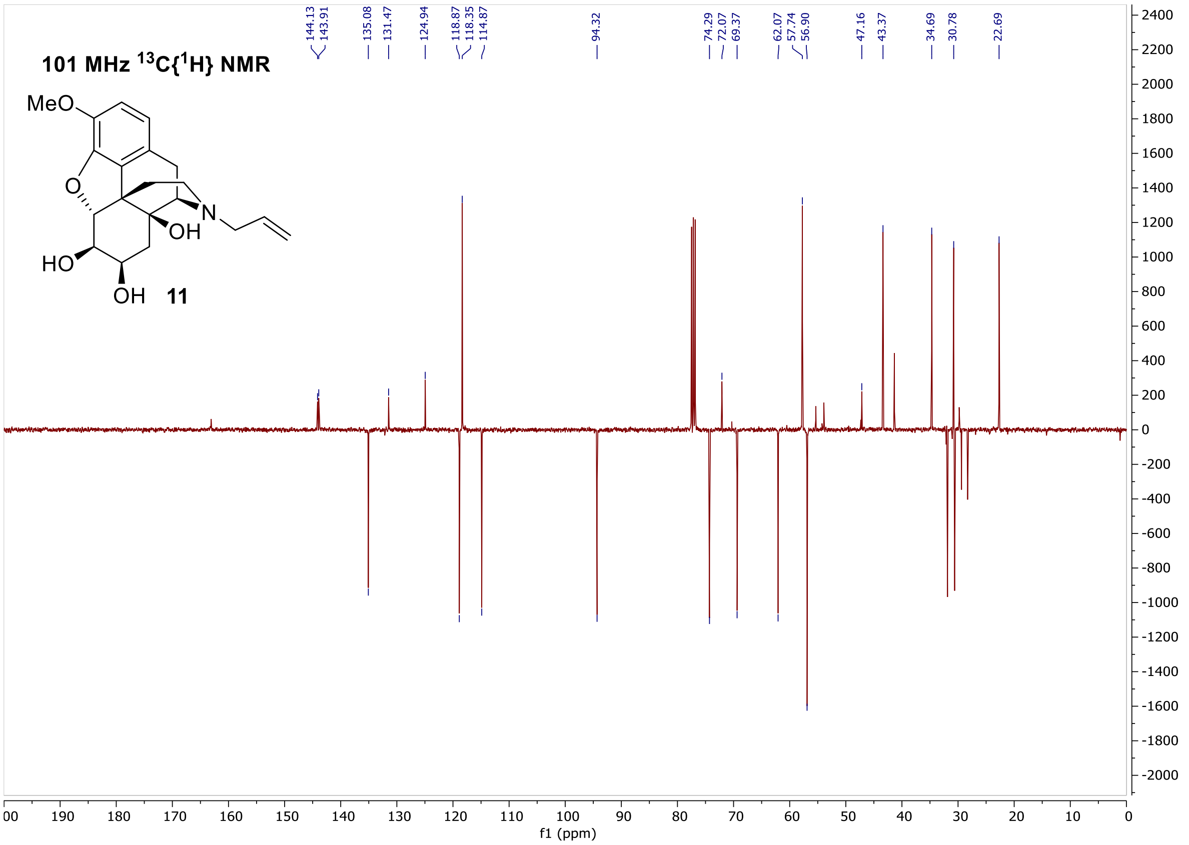
101 MHz ¹³C{¹H} NMR
MeO
O
N
OH
HO
OH
11
144.13
143.91
135.08
131.47
124.94
118.87
118.35
114.87
94.32
74.29
72.07
69.37
62.07
57.74
56.90
47.16
43.37
34.69
30.78
22.69
f1 (ppm)

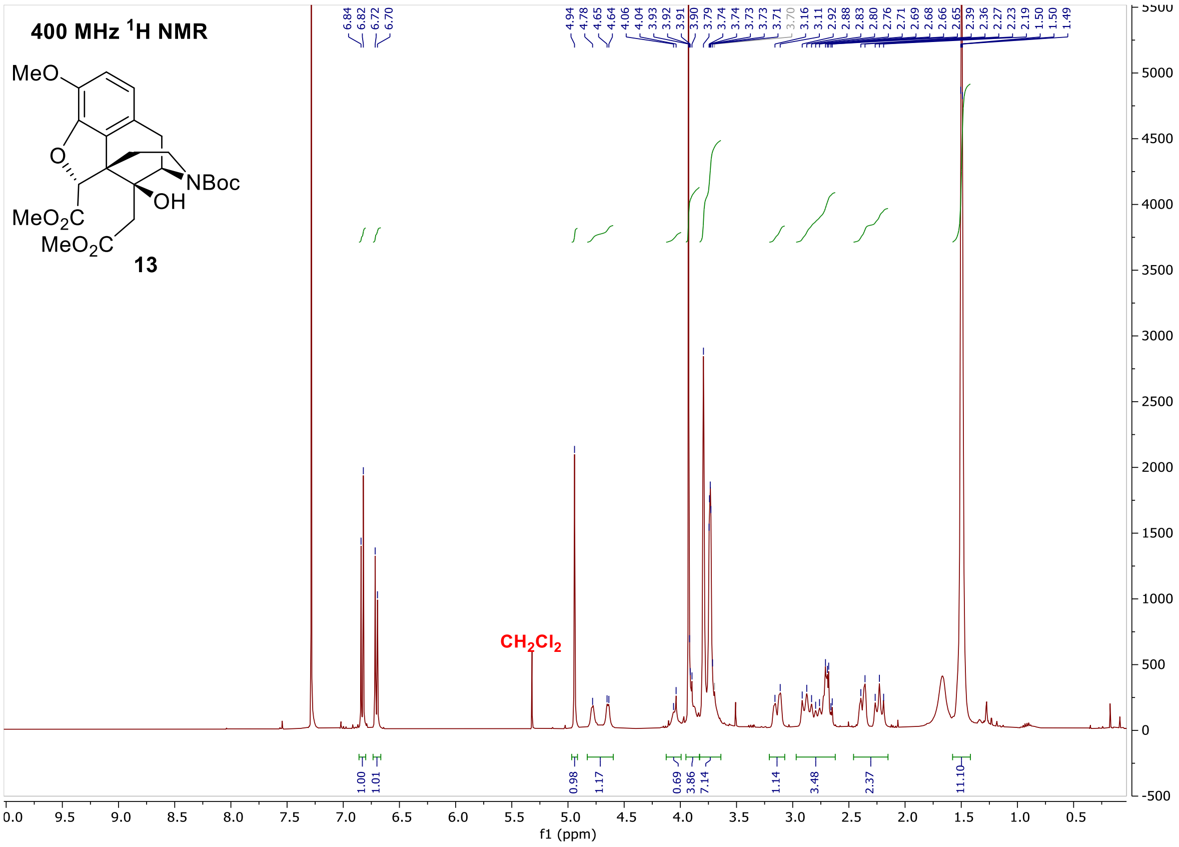
400 MHz ¹H NMR
MeO
O
NBoc
OH
MeO2C
MeO2C
13
CH2Cl2
f1 (ppm)

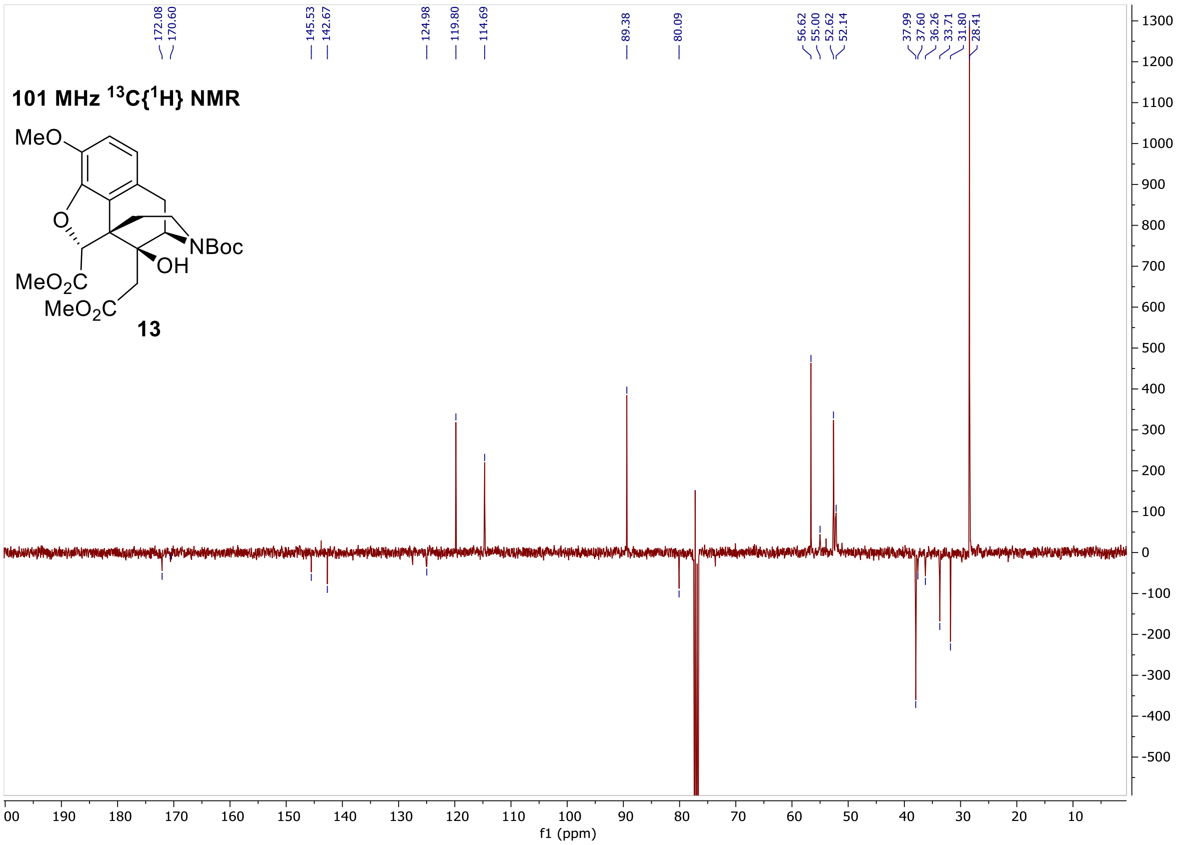
101 MHz ¹³C{¹H} NMR
MeO
O
NBoc
OH
MeO2C
MeO2C
13
f1 (ppm)

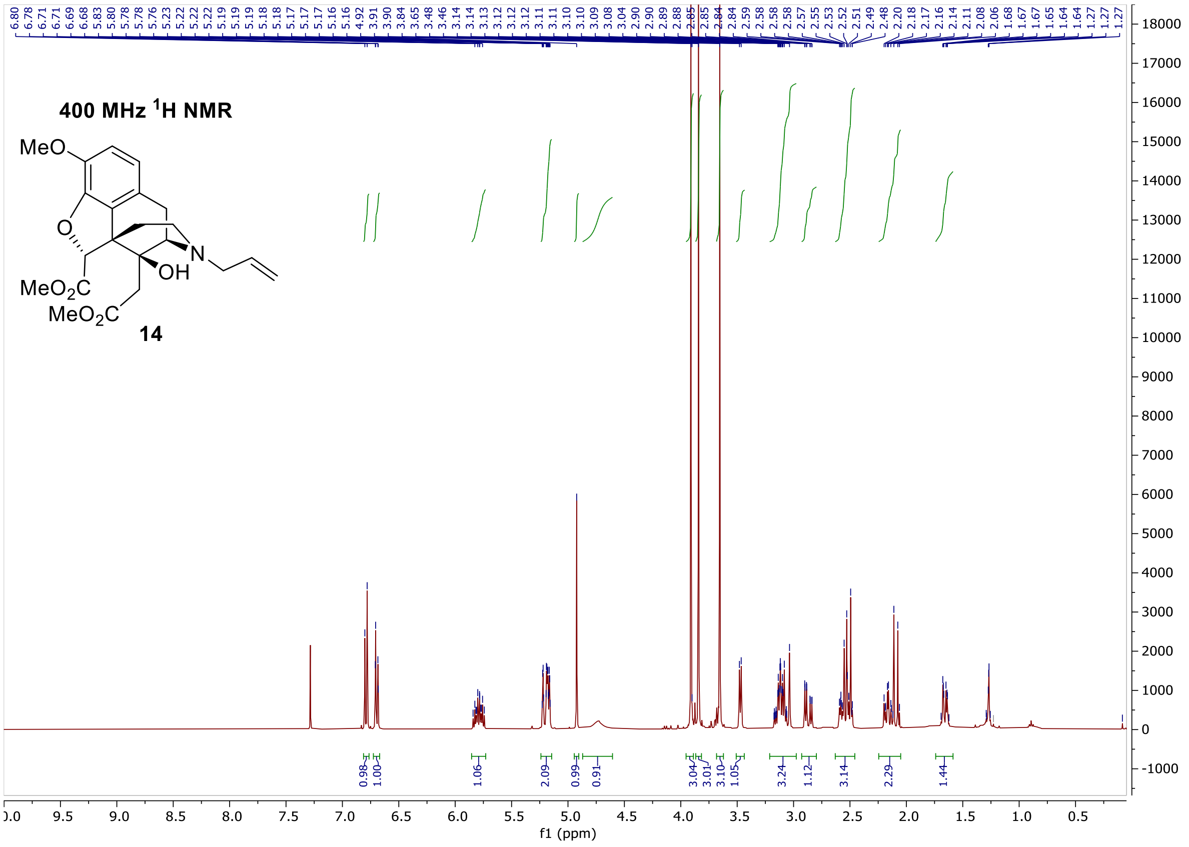
400 MHz ^{1}H NMR
MeO
O
N
OH
MeO$_2$C
MeO$_2$C
14
f1 (ppm)

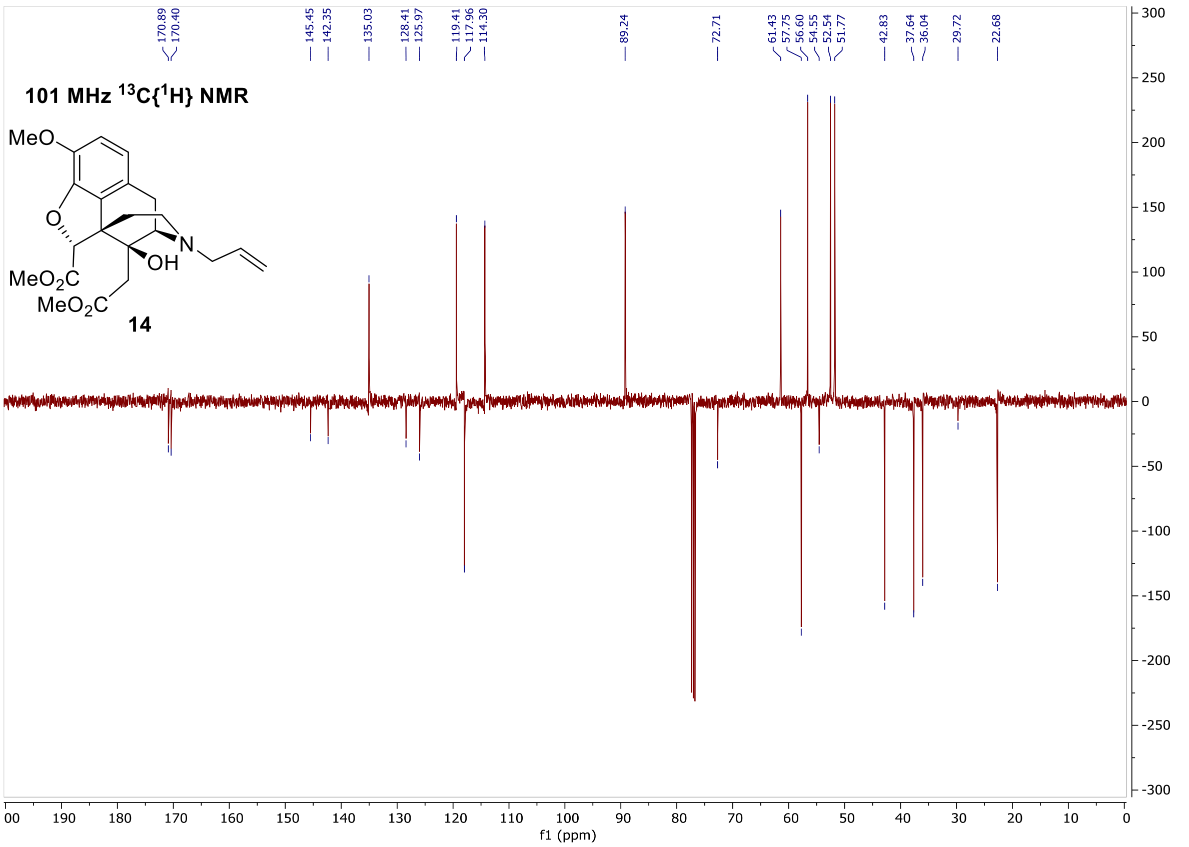
101 MHz ^{13}C{^{1}H} NMR
MeO
O
N
OH
MeO$_2$C
MeO$_2$C
14
170.89
170.40
145.45
142.35
135.03
128.41
125.97
119.41
117.96
114.30
89.24
72.71
61.43
57.75
56.60
54.55
52.54
51.77
42.83
37.64
36.04
29.72
22.68
f1 (ppm)

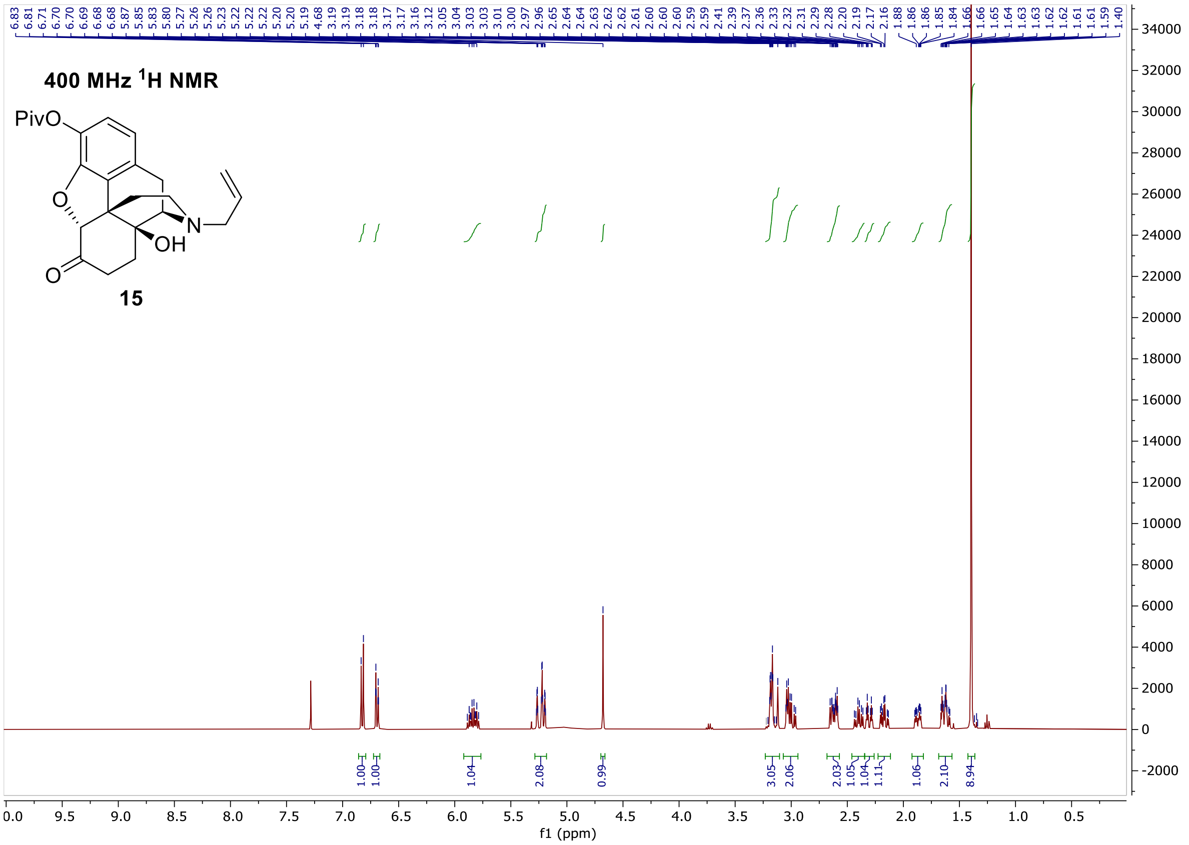
400 MHz ^{1}H NMR
PivO
O
OH
N
O
15
f1 (ppm)

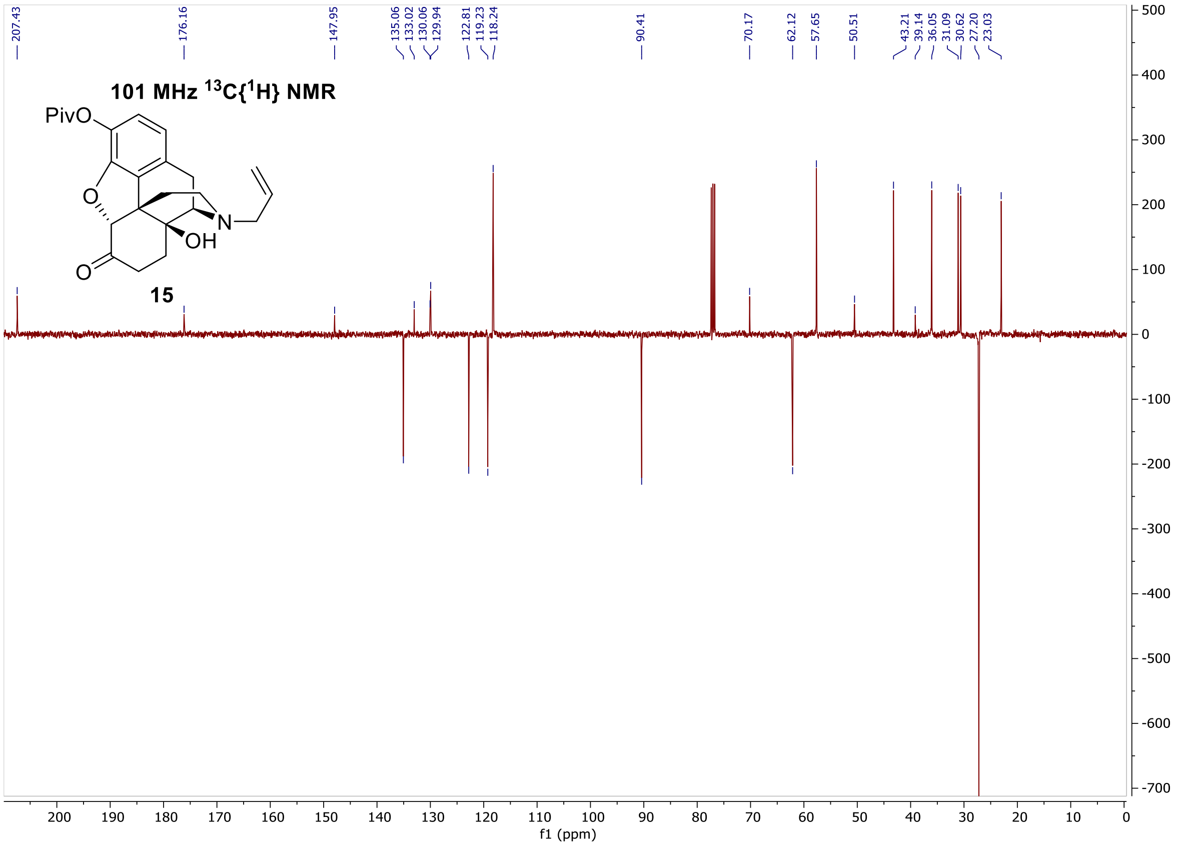
207.43
176.16
147.95
135.06
133.02
130.06
129.94
122.81
119.23
118.24
90.41
70.17
62.12
57.65
50.51
43.21
39.14
36.05
31.09
30.62
27.20
23.03
101 MHz ^{13}C{^{1}H} NMR
PivO
O
OH
N
O
15
f1 (ppm)

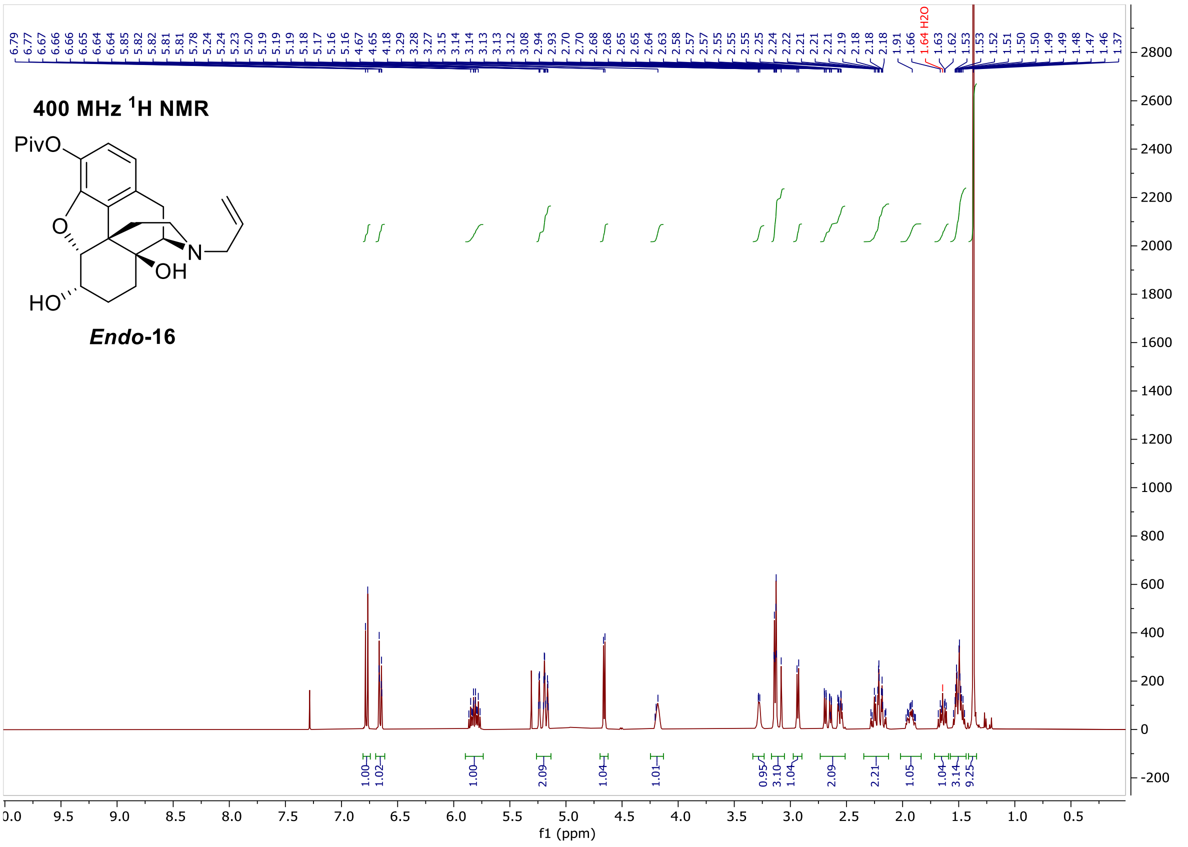

400 MHz 1H NMR
PivO
O
N
OH
HO
Endo-16
6.79 6.77 6.67 6.66 6.66 6.65 6.64 6.64 5.85 5.82 5.82 5.81 5.81 5.78 5.24 5.24 5.23 5.20 5.19 5.19 5.19 5.18 5.17 5.16 5.16 4.67 4.65 4.18 3.29 3.28 3.27 3.15 3.14 3.14 3.13 3.13 3.12 3.08 2.94 2.93 2.70 2.70 2.68 2.68 2.65 2.65 2.64 2.63 2.58 2.57 2.57 2.55 2.55 2.55 2.25 2.24 2.22 2.21 2.21 2.21 2.19 2.18 2.18 2.18 1.91 1.66 1.64 H2O 1.63 1.62 1.53 1.53 1.52 1.51 1.50 1.50 1.49 1.49 1.48 1.47 1.46 1.37
1.00 1.02 1.00 2.09 1.04 1.01 0.95 3.10 1.04 2.09 2.21 1.05 1.04 3.14 9.25
f1 (ppm)

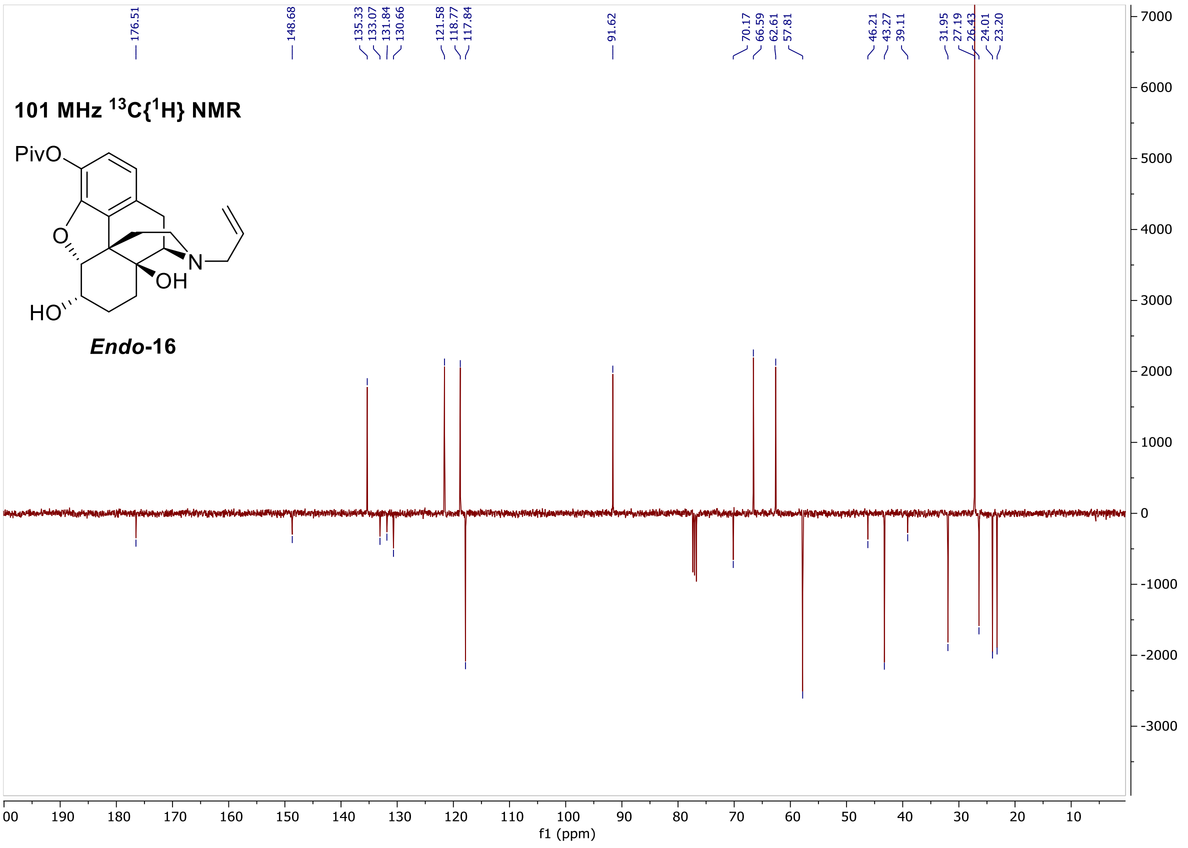

101 MHz 13C{1H} NMR
PivO
O
N
OH
HO
Endo-16
176.51 148.68 135.33 133.07 131.84 130.66 121.58 118.77 117.84 91.62 70.17 66.59 62.61 57.81 46.21 43.27 39.11 31.95 27.19 26.43 24.01 23.20
f1 (ppm)

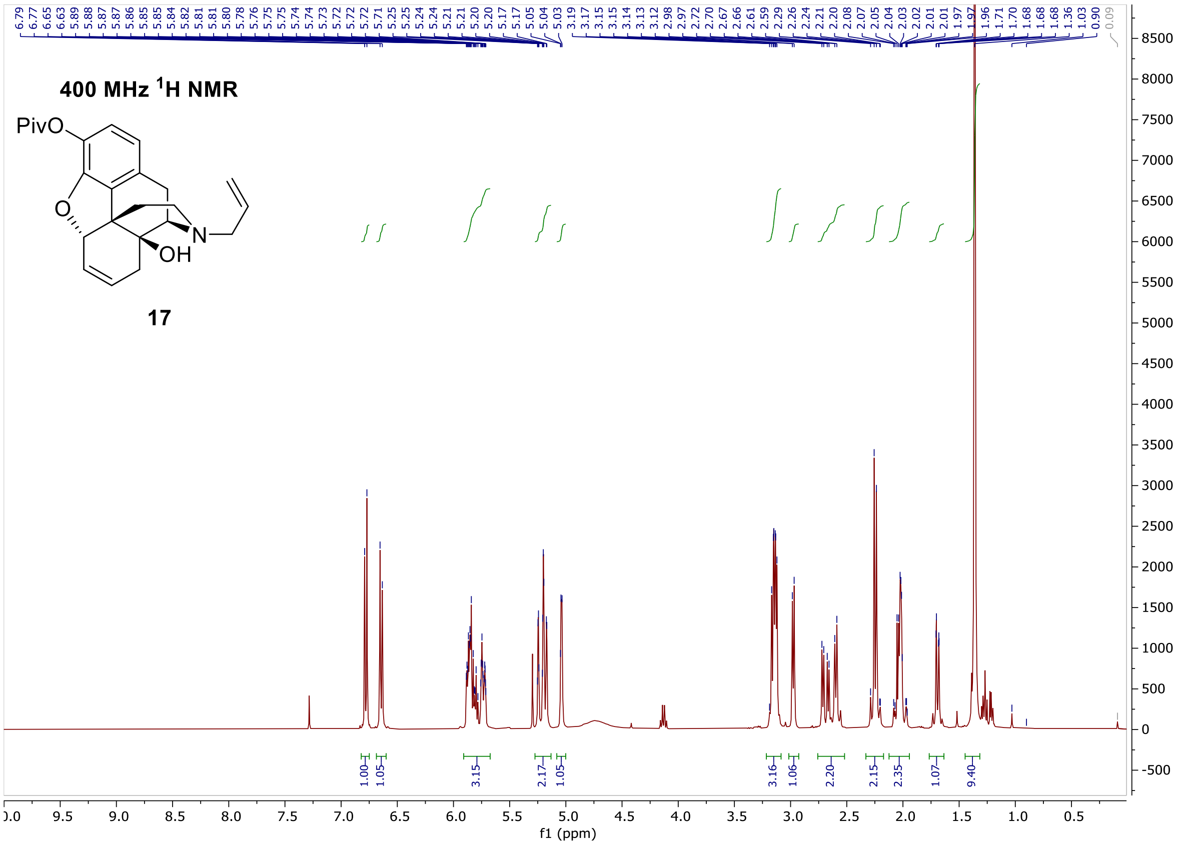

400 MHz ¹H NMR
PivO
O
N
OH
17
f1 (ppm)

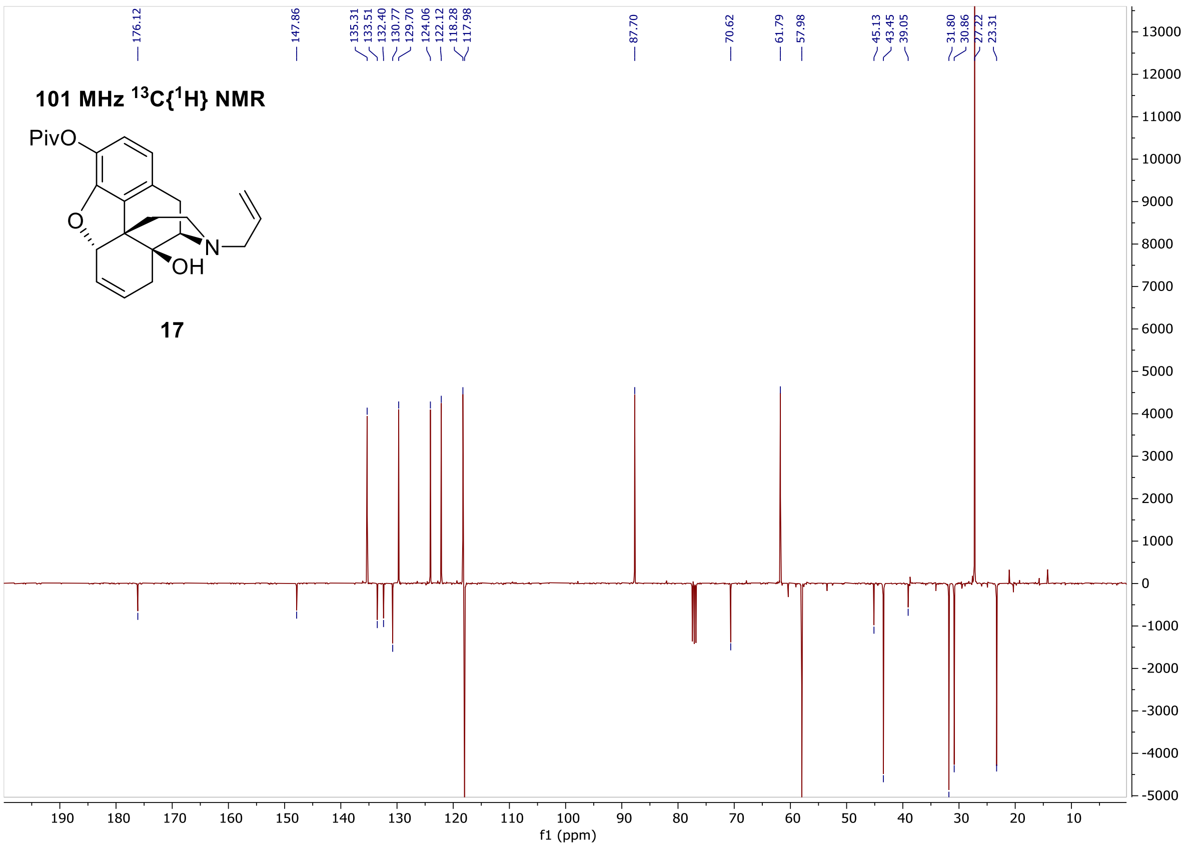

101 MHz ¹³C{¹H} NMR
PivO
O
N
OH
17
176.12
147.86
135.31
133.51
132.40
130.77
129.70
124.06
122.12
118.28
117.98
87.70
70.62
61.79
57.98
45.13
43.45
39.05
31.80
30.86
27.22
23.31
f1 (ppm)

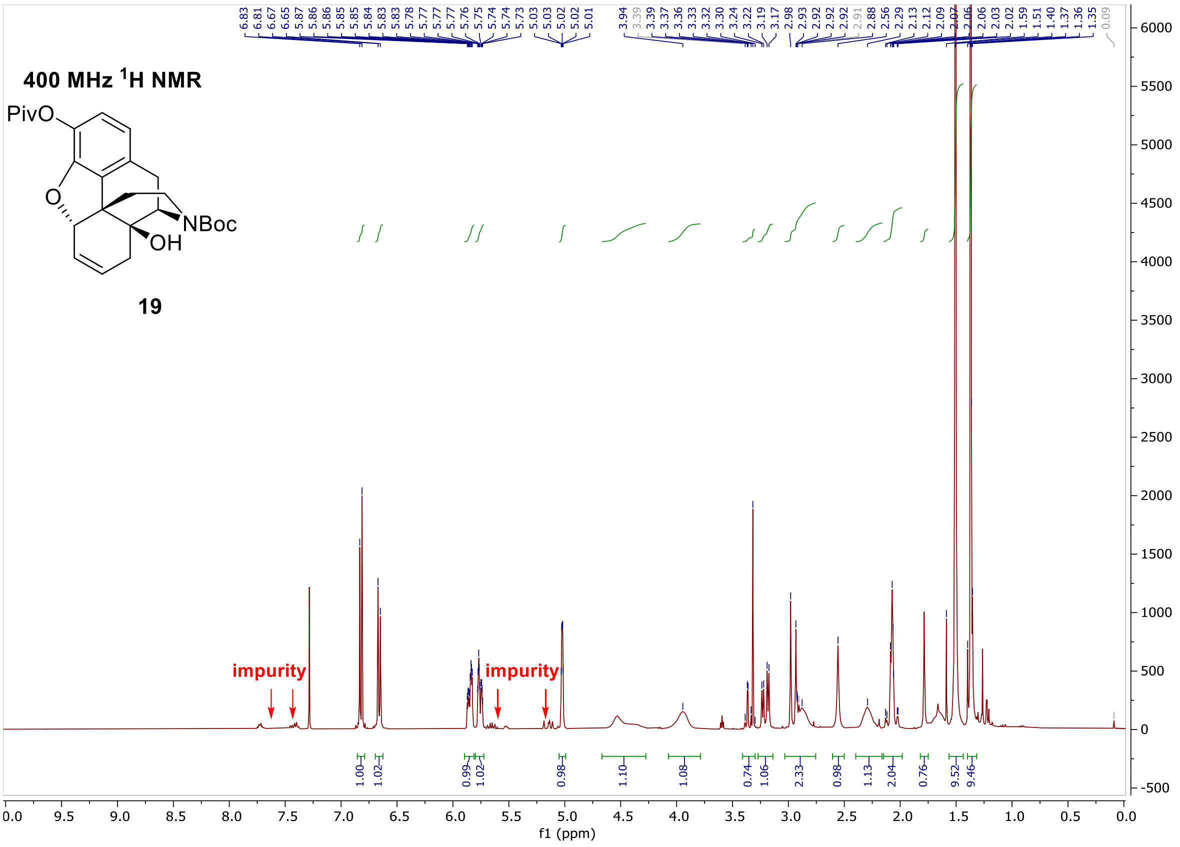
400 MHz 1H NMR
PivO
O
NBoc
OH
19
impurity
impurity
f1 (ppm)

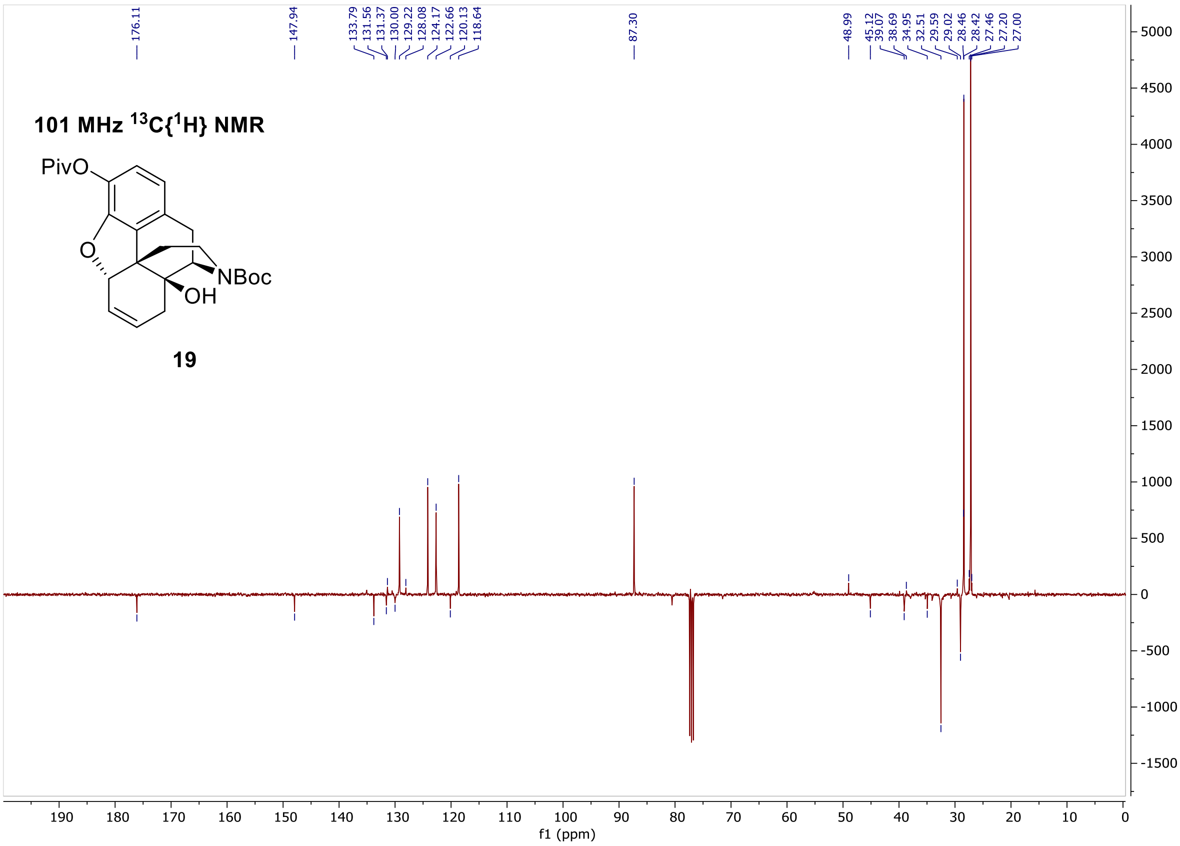
101 MHz 13C{1H} NMR
PivO
O
NBoc
OH
19
176.11
147.94
133.79
131.56
131.37
130.00
129.22
128.08
124.17
122.66
120.13
118.64
87.30
48.99
45.12
39.07
38.69
34.95
32.51
29.59
29.02
28.46
28.42
27.46
27.20
27.00
f1 (ppm)

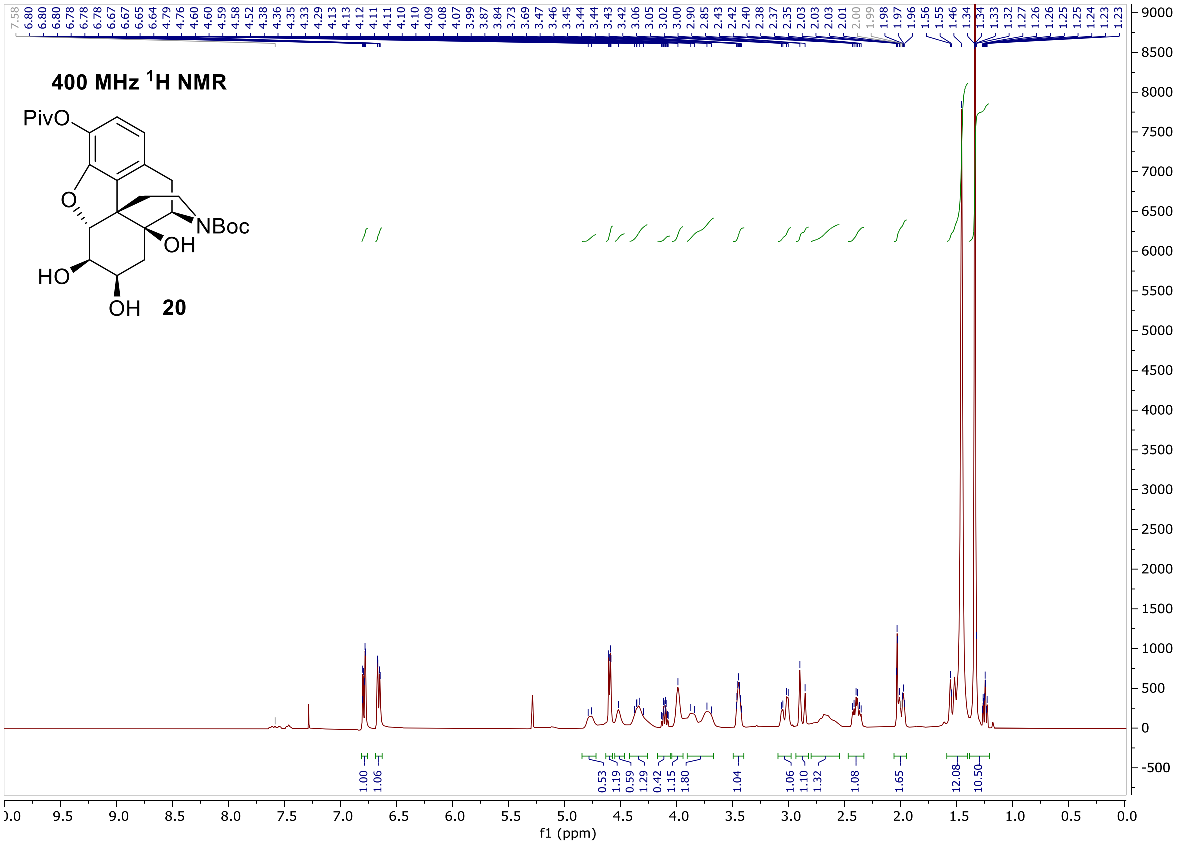
400 MHz ^{1}H NMR
PivO
O
NBoc
OH
HO
OH
20
f1 (ppm)

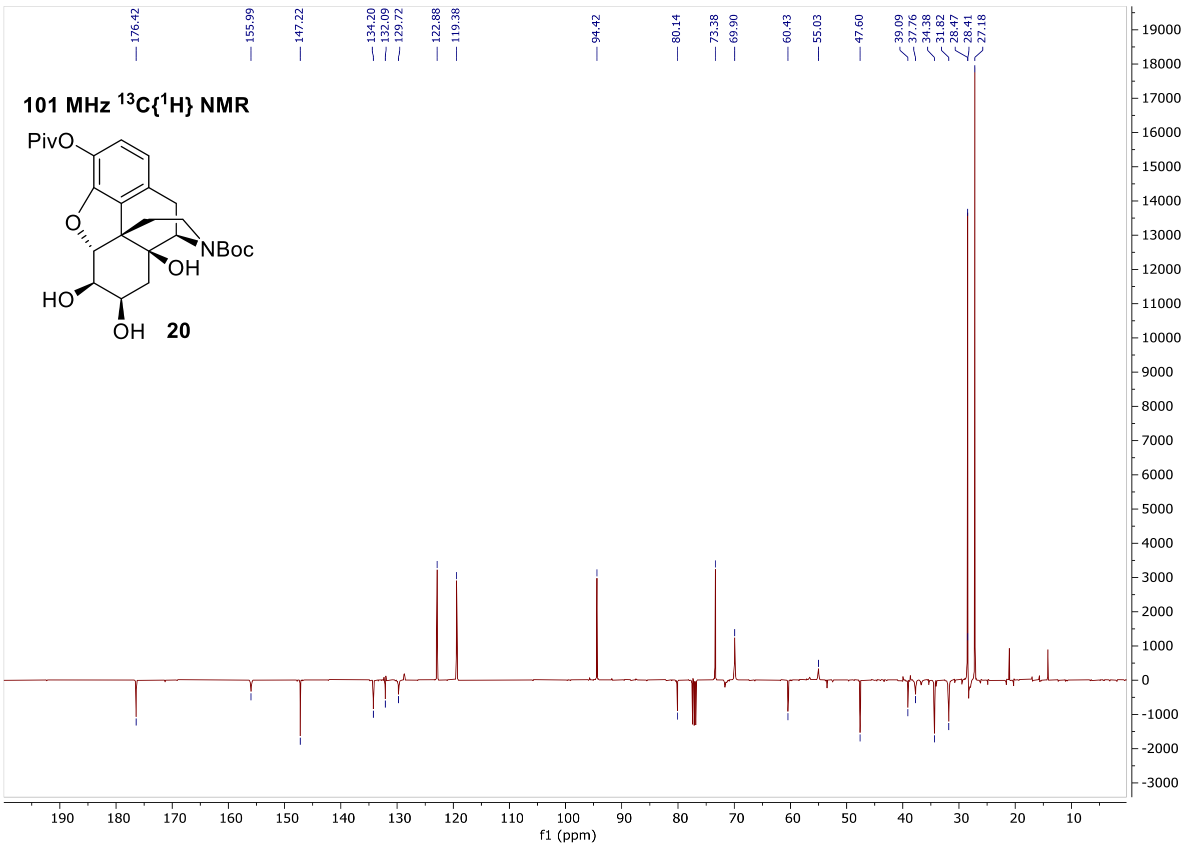
101 MHz ^{13}C{^{1}H} NMR
176.42
155.99
147.22
134.20
132.09
129.72
122.88
119.38
94.42
80.14
73.38
69.90
60.43
55.03
47.60
39.09
37.76
34.38
31.82
28.47
28.41
27.18
PivO
O
NBoc
OH
HO
OH
20
f1 (ppm)

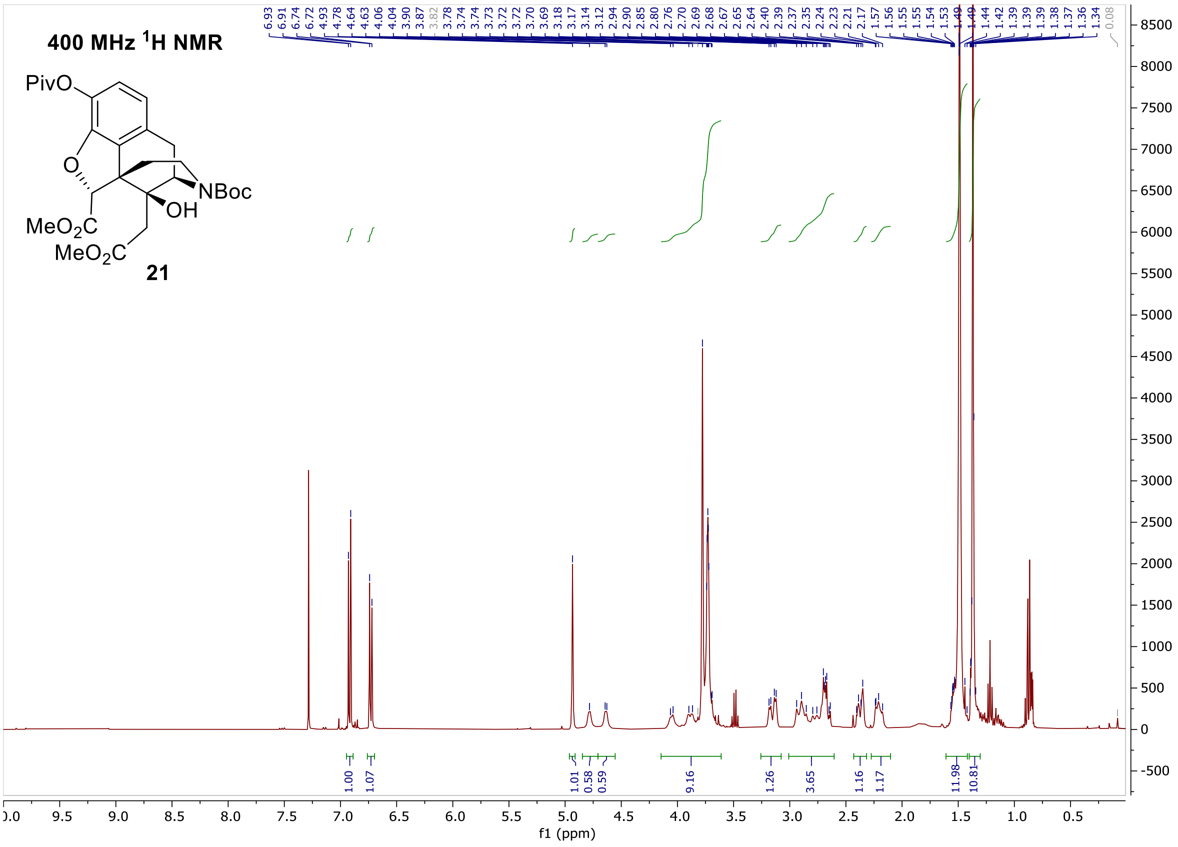
400 MHz 1H NMR
PivO
O
NBoc
OH
MeO2C
MeO2C
21
f1 (ppm)

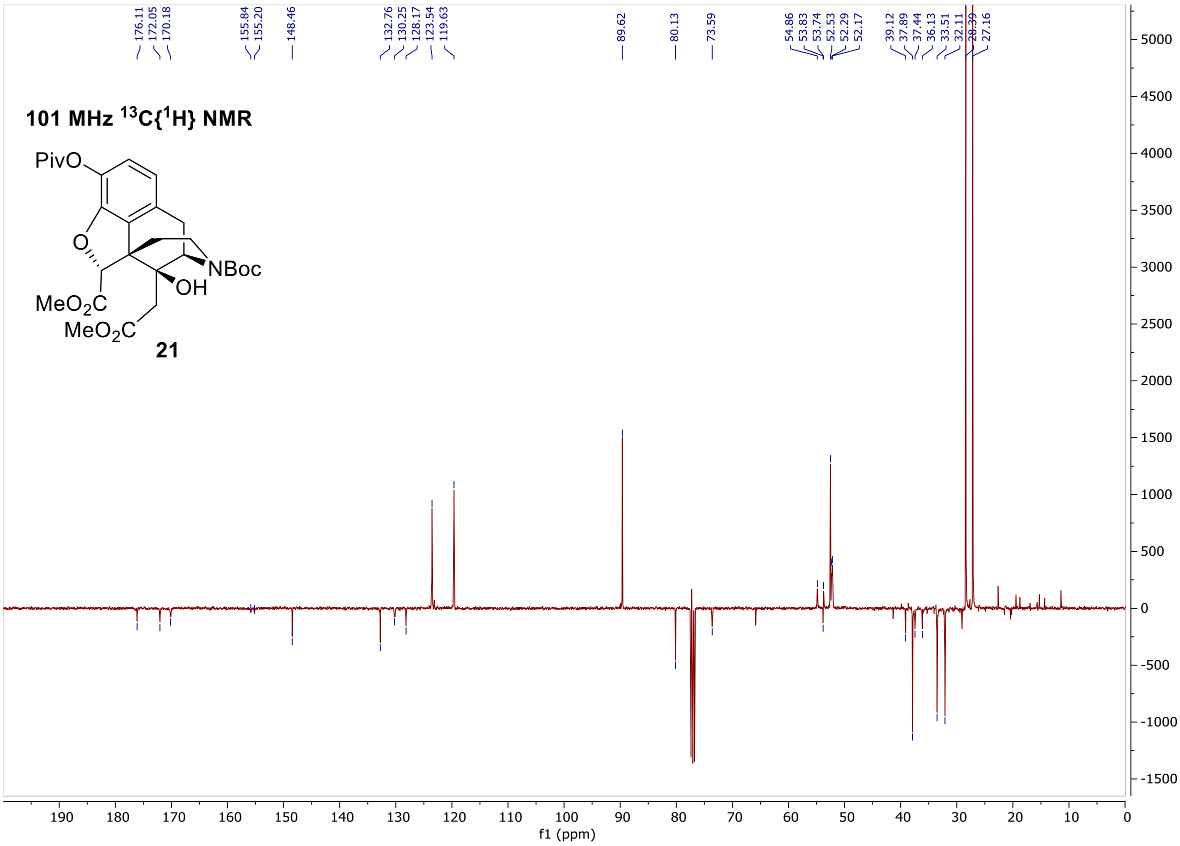
101 MHz 13C{1H} NMR
PivO
O
NBoc
OH
MeO2C
MeO2C
21
f1 (ppm)

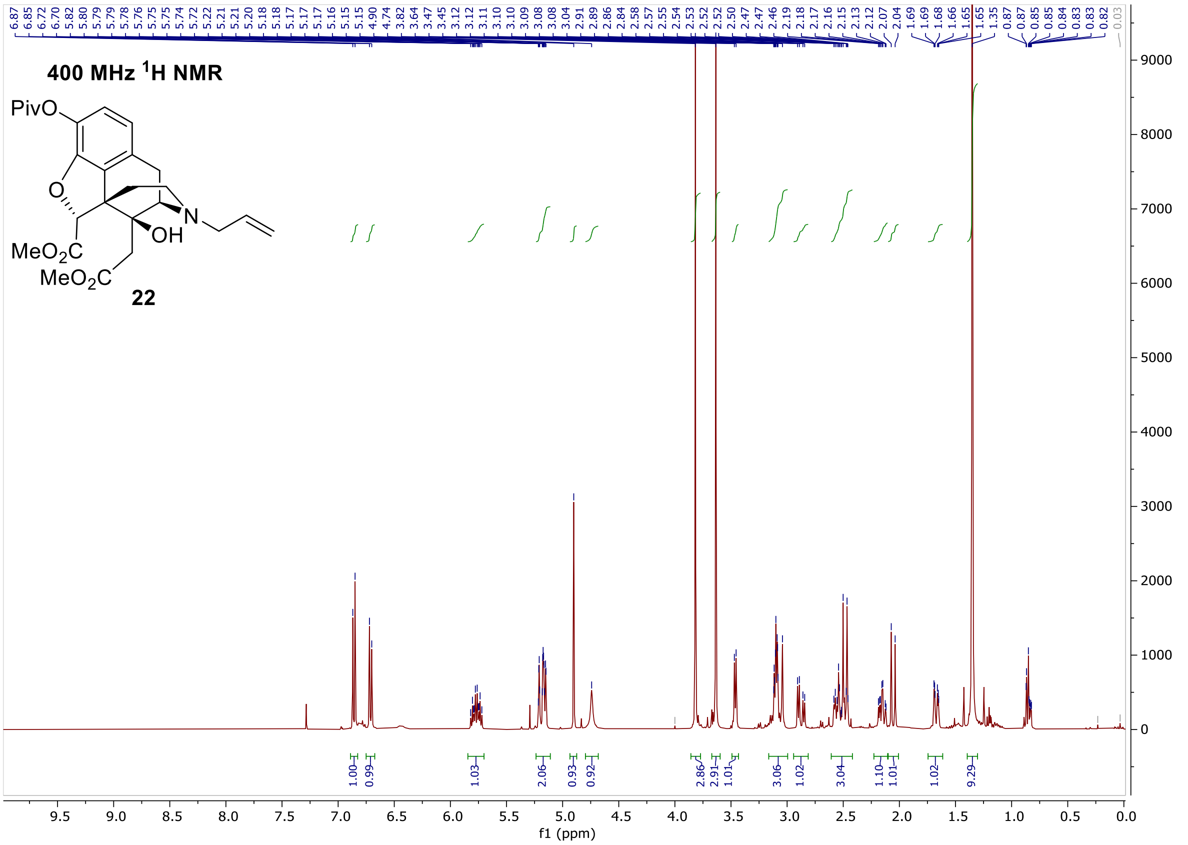
400 MHz ¹H NMR
PivO
O
N
OH
MeO2C
MeO2C
22
f1 (ppm)

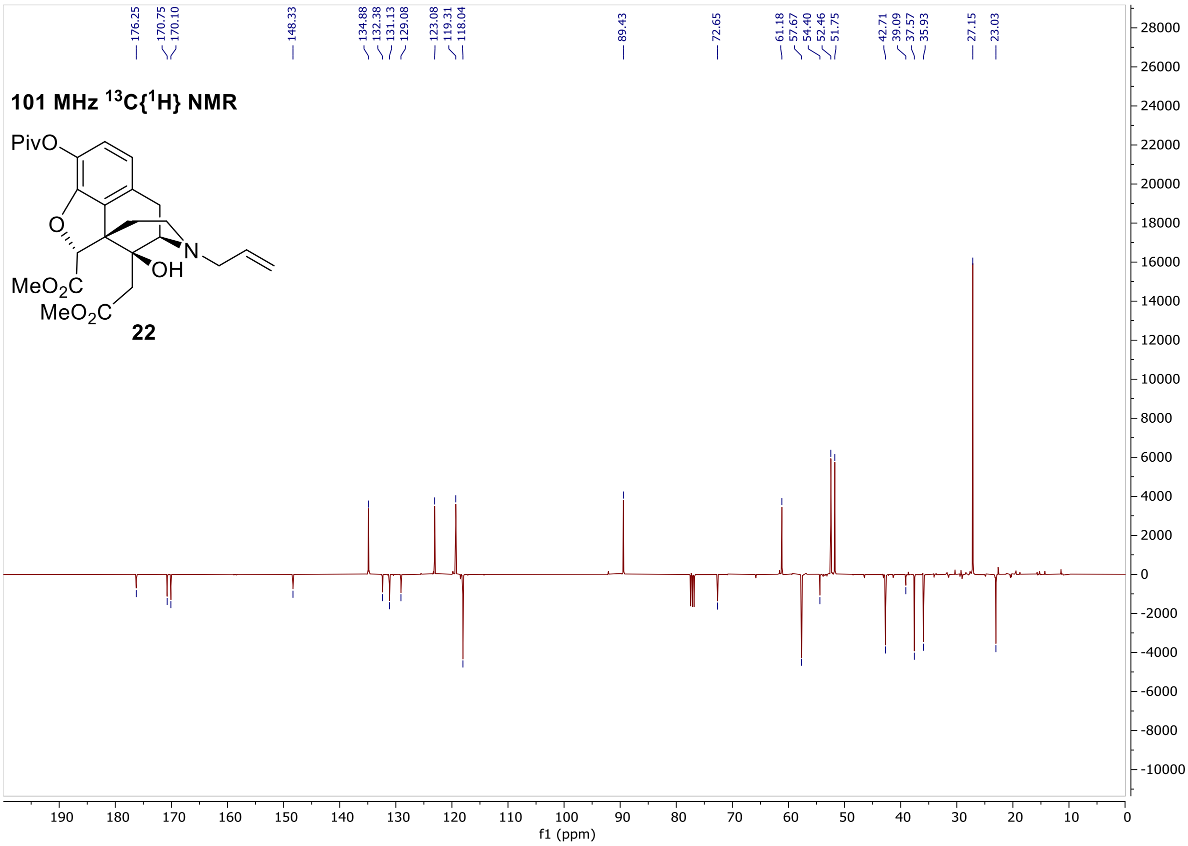
101 MHz ¹³C{¹H} NMR
PivO
O
N
OH
MeO2C
MeO2C
22
f1 (ppm)

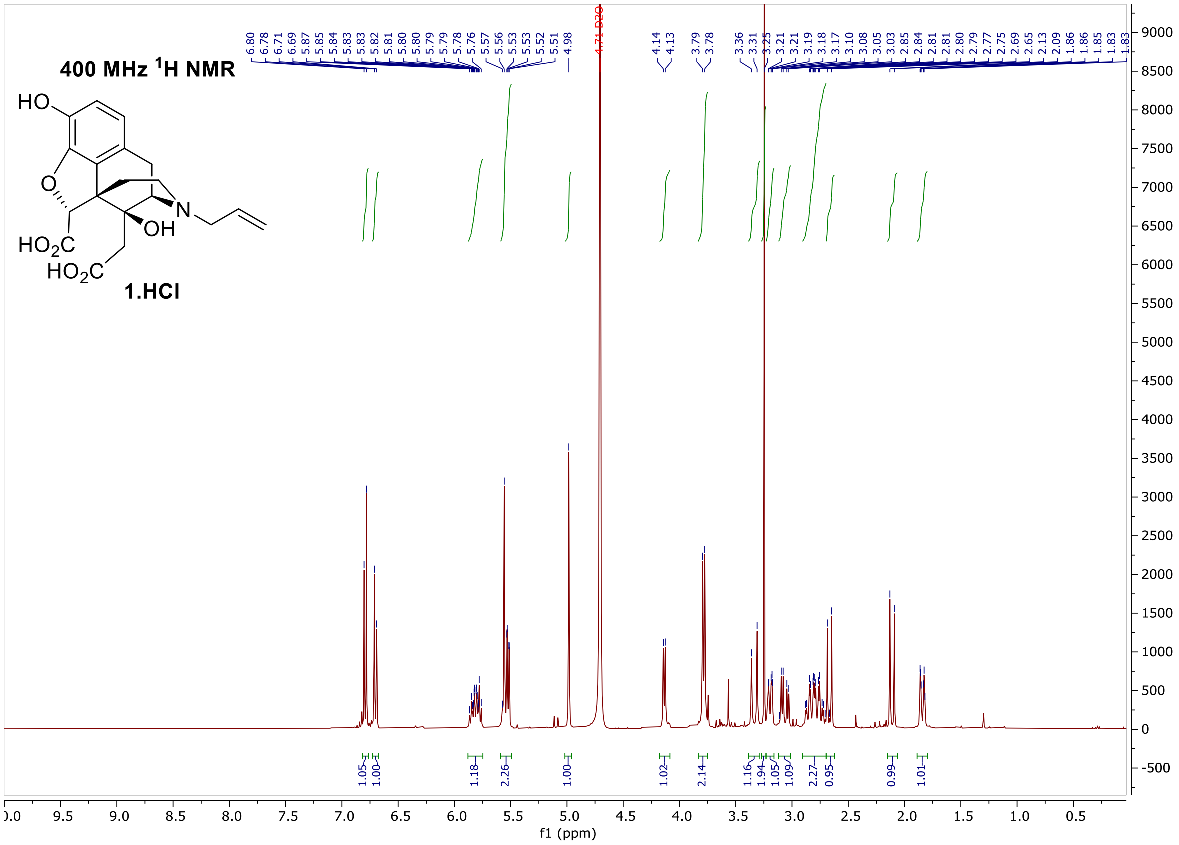
400 MHz ¹H NMR
HO
O
N
OH
HO₂C
HO₂C
1.HCl
f1 (ppm)

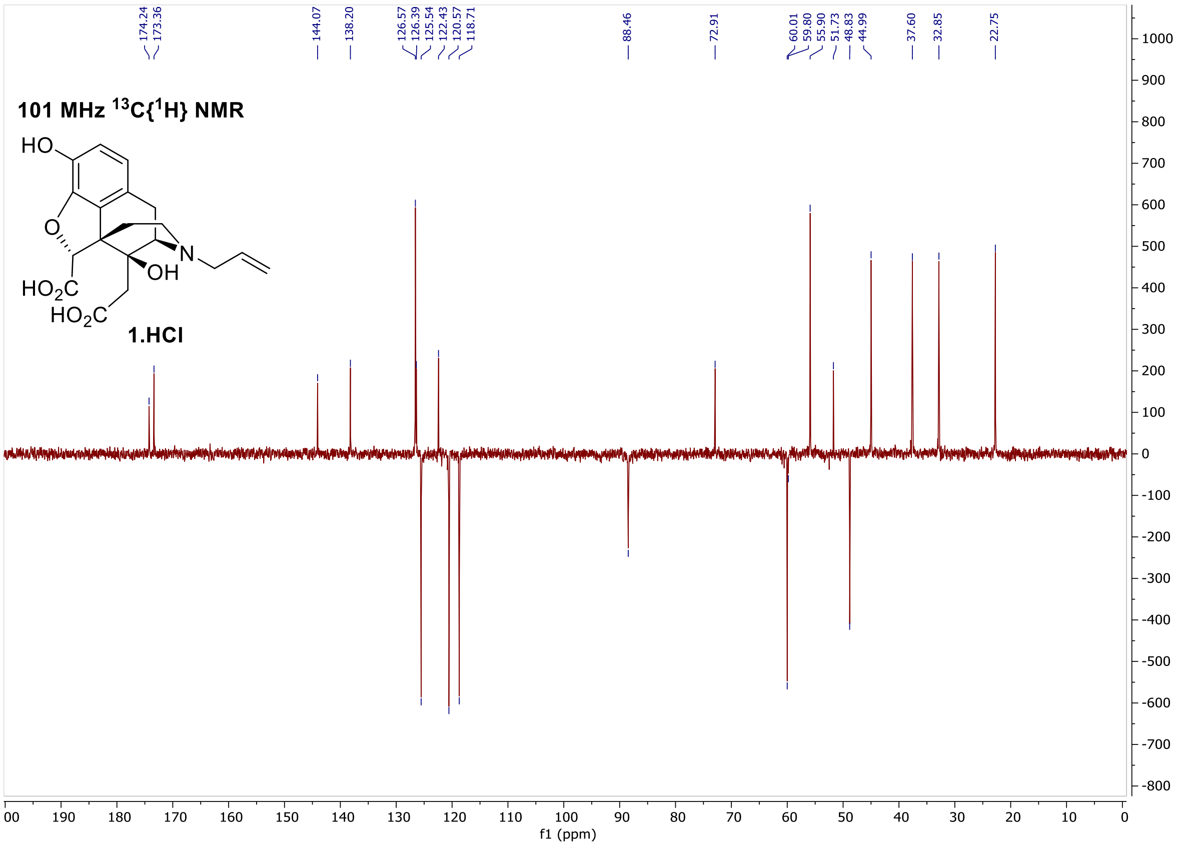
101 MHz ¹³C{¹H} NMR
HO
O
N
OH
HO₂C
HO₂C
1.HCl
f1 (ppm)

^{1}H NMR spectrum of diacid **1** produced by forced degradation

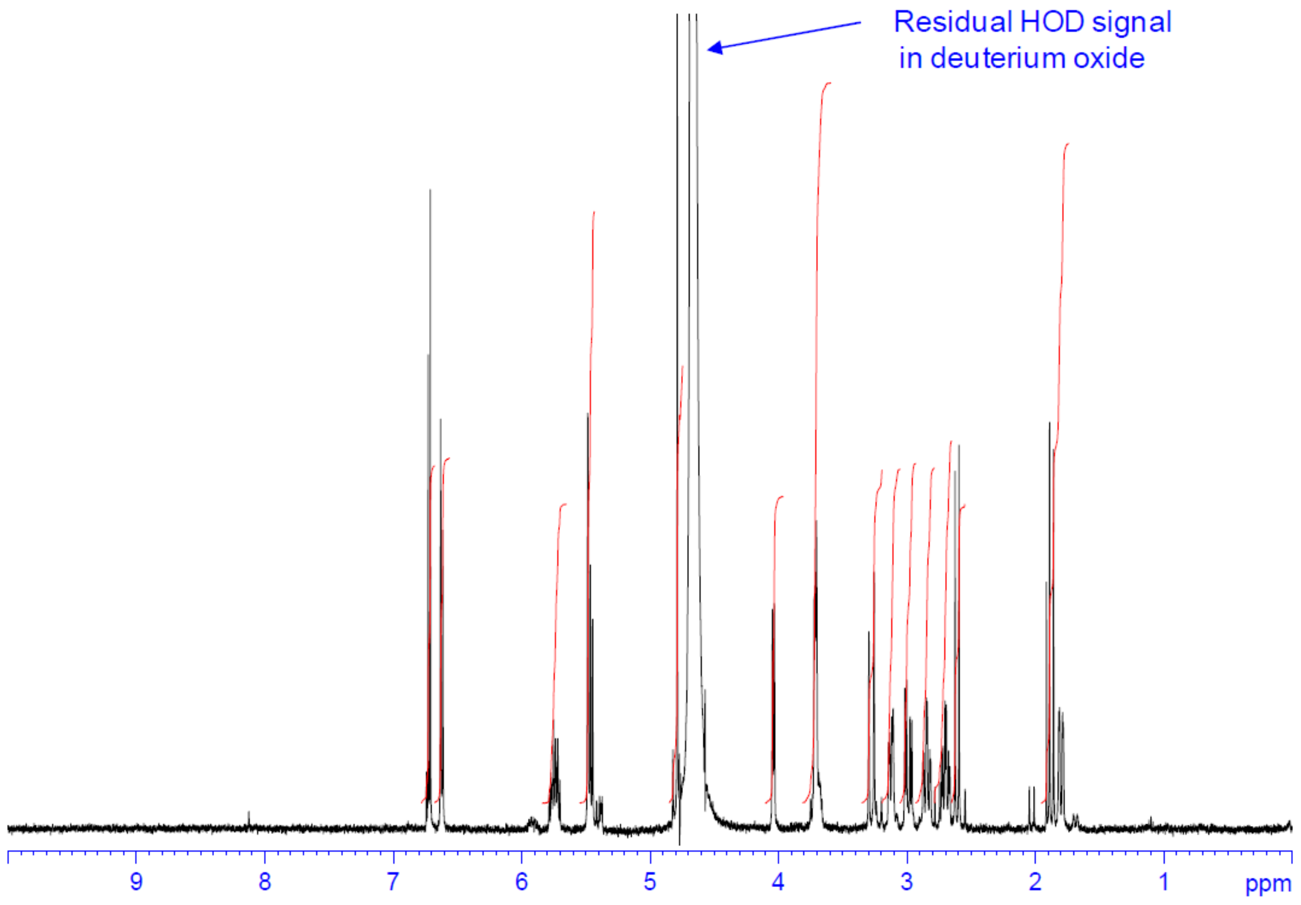

HPLC Analysis of Degradant E and Compound 1

HPLC (Gemini C18, 1% TFA in water/acetonitrile = 100/0 to 20/80, flow rate = 0.5 mL/min, l = 288 nm) tR = 8.54 min. Details of the drug product HPLC conditions and a typical HPLC chromatogram have been reported.[1]

Figure S1 shows the HPLC trace of compound **1** and *Figure S2* shows the HPLC trace of the degraded sample alone (unspiked sample) and the HPLC trace of compound **1** added to the degraded sample (spiked sample). The increased peak at 8.54 minutes shows an increase in the concentration of *Degradant E* in the mixture, which confirms the presence of this impurity in the degraded sample. Table S1 compiles the fragmentation patterns from mass spectrometric analysis of compound **1** as compared to the degraded sample.

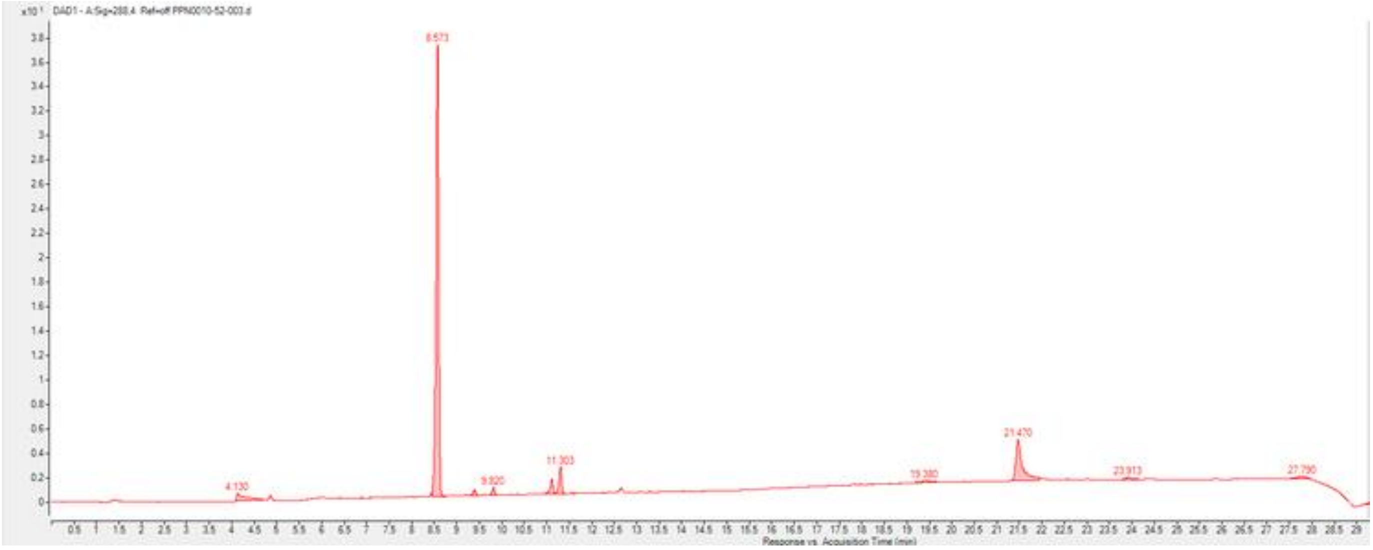

Figure S1: HPLC trace of synthesised compound **1**

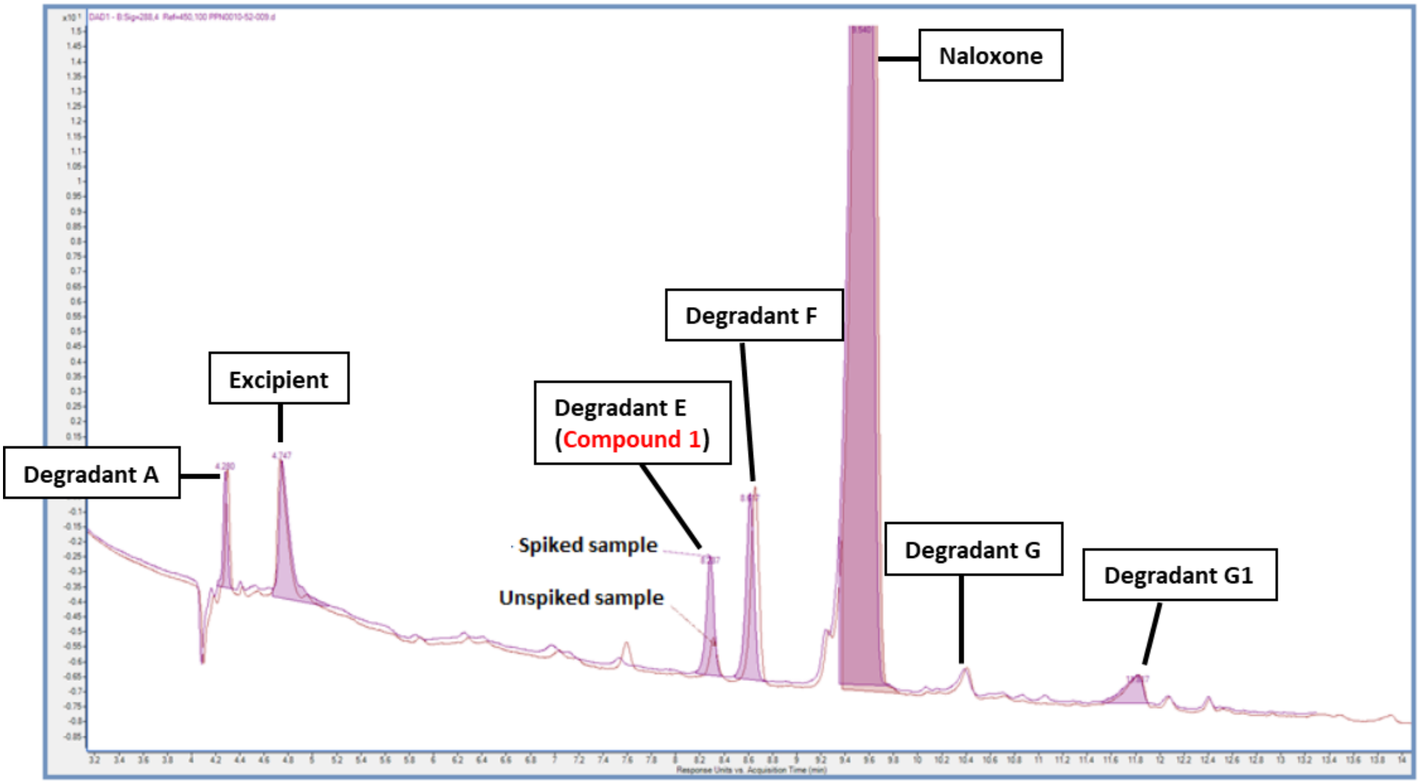

Figure S2: HPLC trace of the degraded sample and degraded sample + **1**

[1] Byard, S. J.; Carey, J. S. *Org. Process Res. Dev.* **2024**, *28*, 3645-3660.

Table S1. Comparison of the Mass Spectrometry Fragmentation Patterns of authentic sample of diacid 1 with Degradant E.

Authentic Sample of Diacid 1	Degradant E	Proposed Formula
376.1384	376.13859	$C_{19}H_{22}NO_7$
340.1186	340.11729	$C_{19}H_{18}NO_5$
330.1339	330.13298	$C_{18}H_{20}NO_5$
314.1389	314.13805	$C_{18}H_{20}NO_4$
284.0921	284.09112	$C_{16}H_{14}NO_4$
272.0915	272.09116	$C_{15}H_{14}NO_4$
214.0864	214.08563	$C_{13}H_{12}NO_2$
185.0595	185.05891	$C_{12}H_9O_2$

Structural assignment of compound 6

Structural assignment of compound **6** was inferred based on $^{1}H/^{13}C$ shift values against the known transformation of naloxone methyl ether:

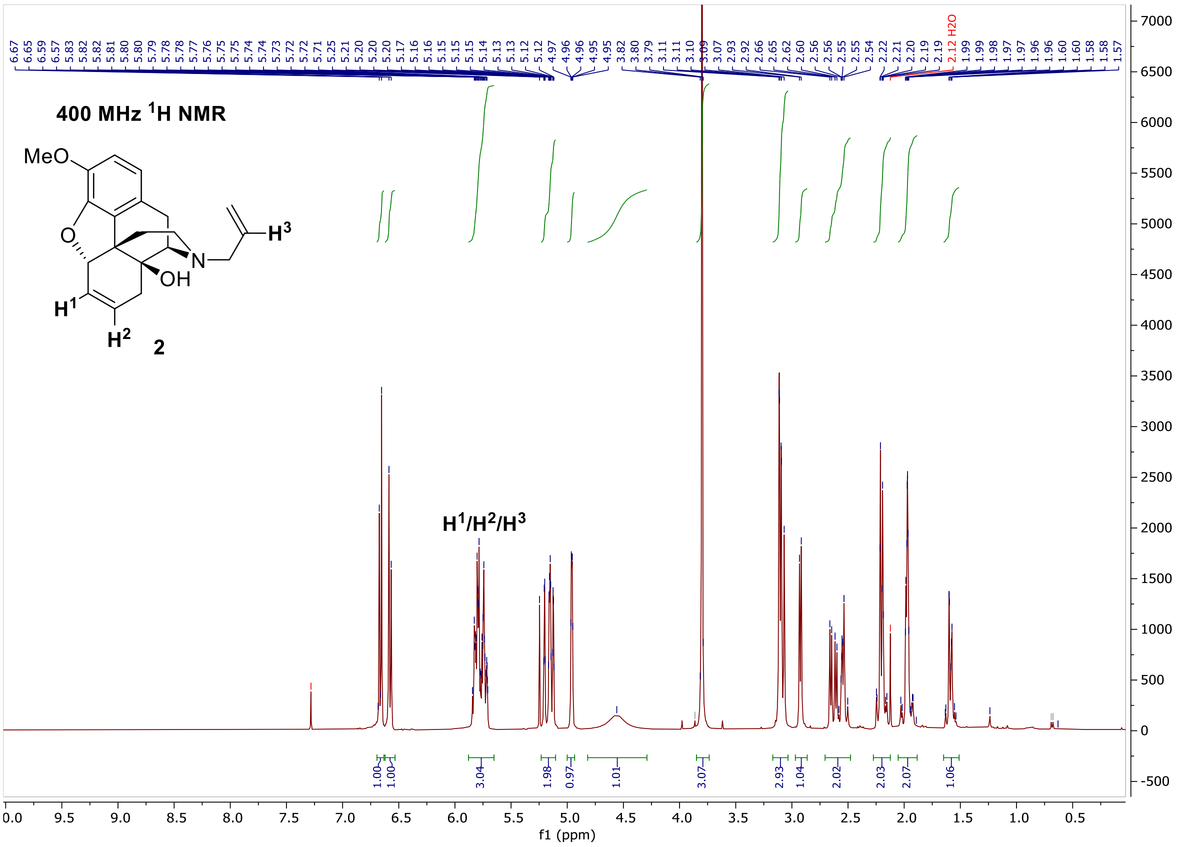

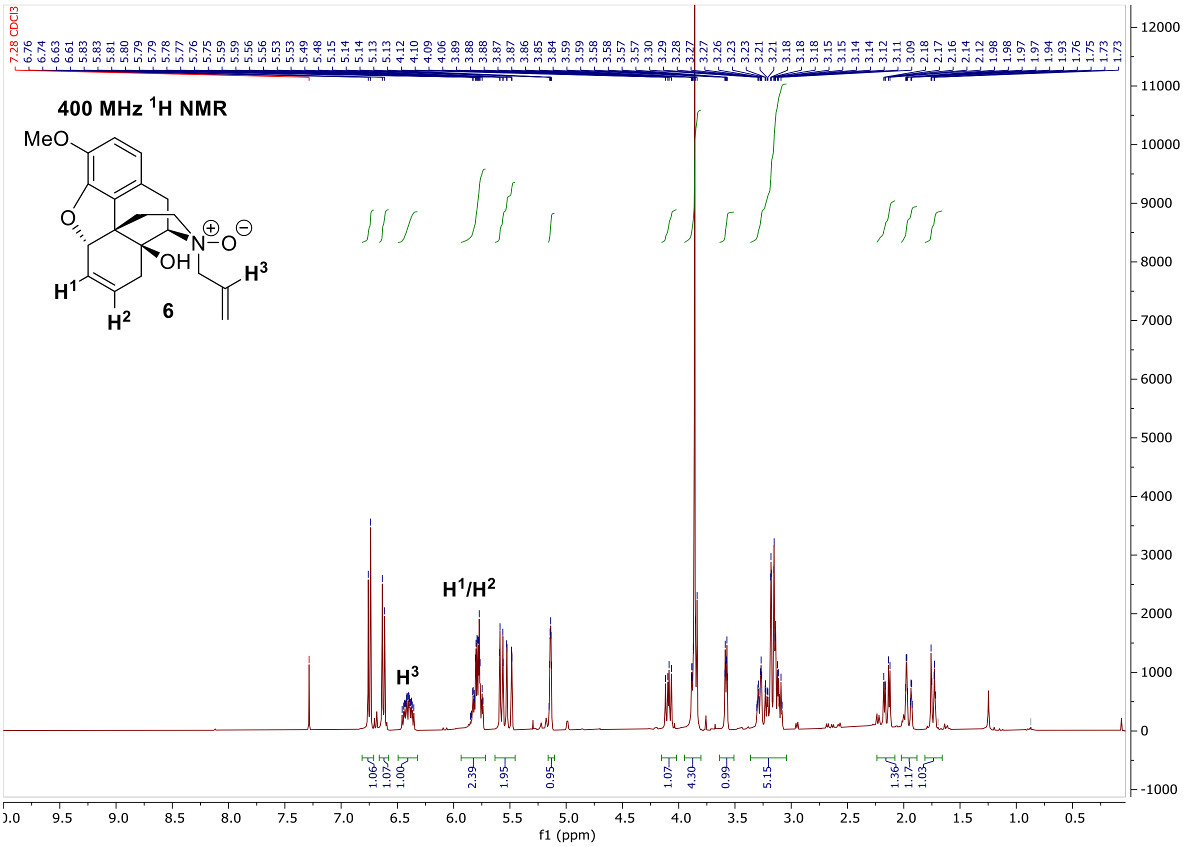

Naloxone methyl ether

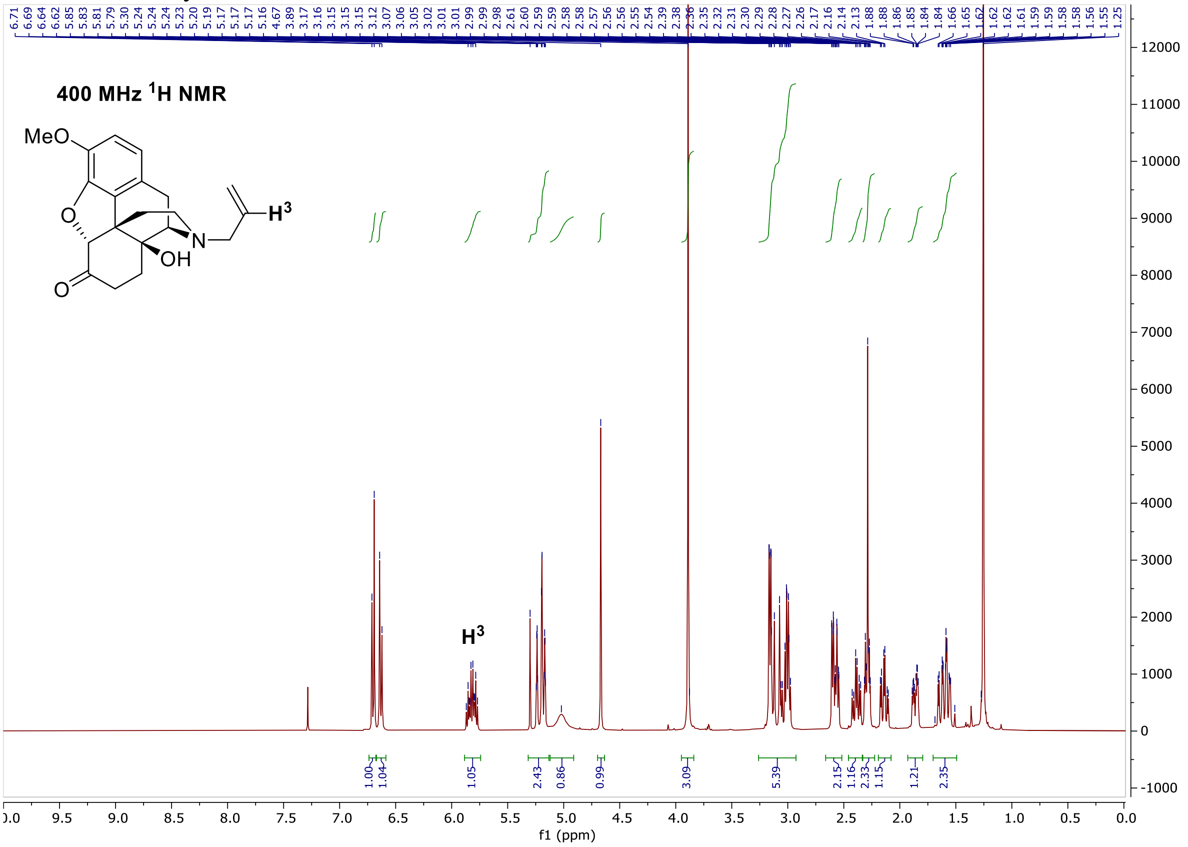

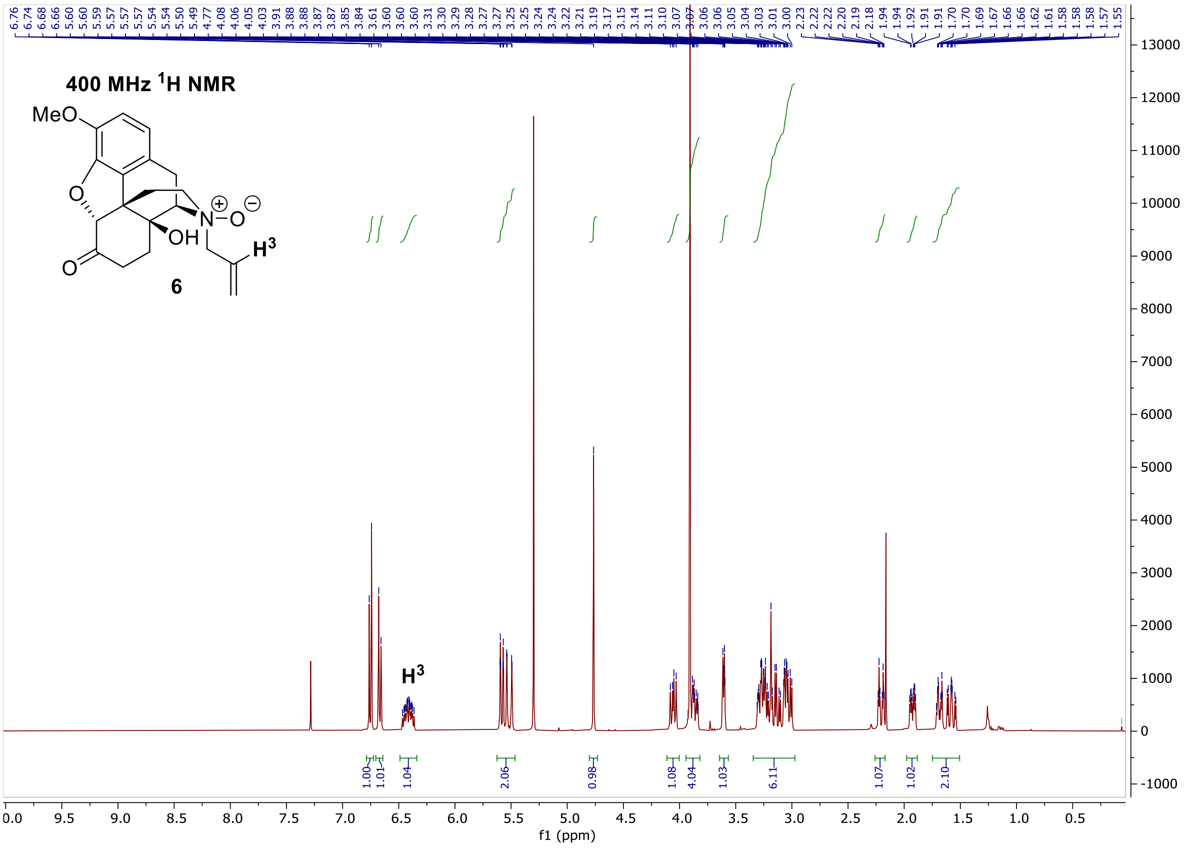

^{13}C spectra of Compounds **2** and **6**

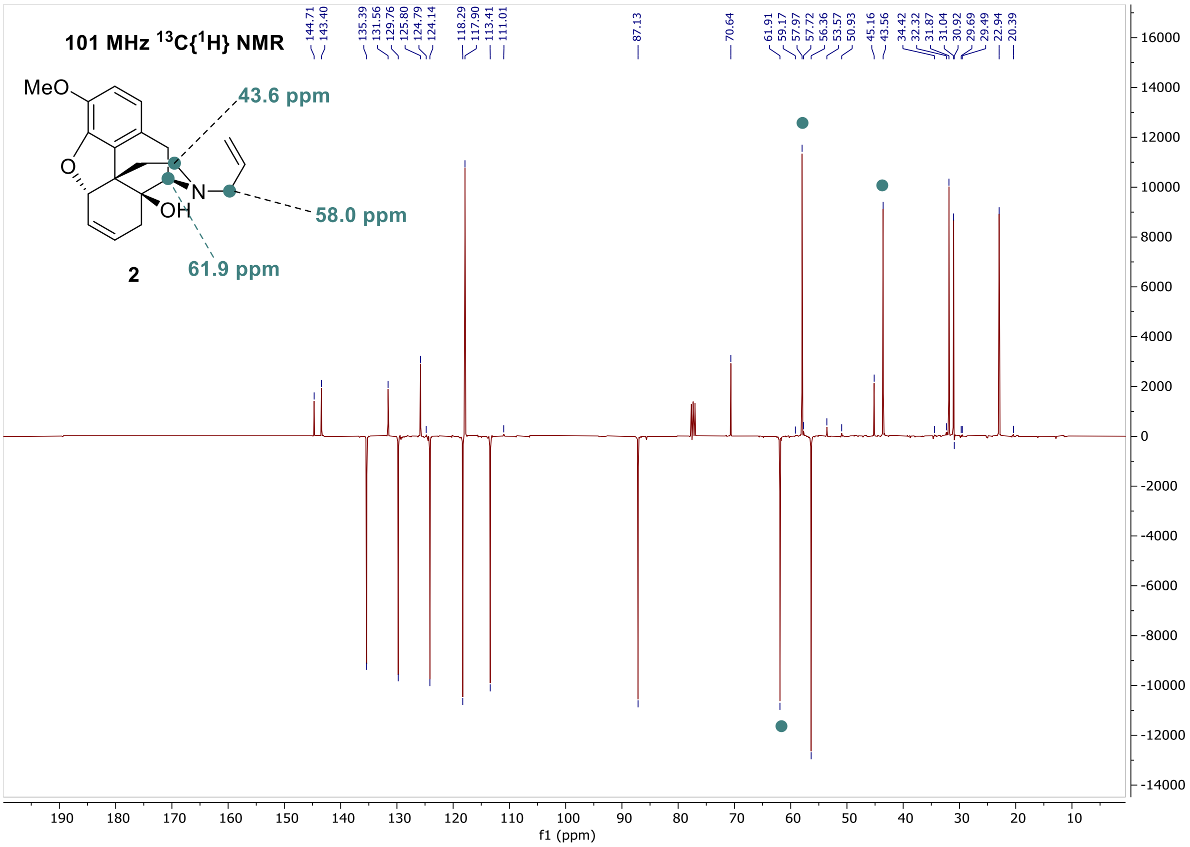

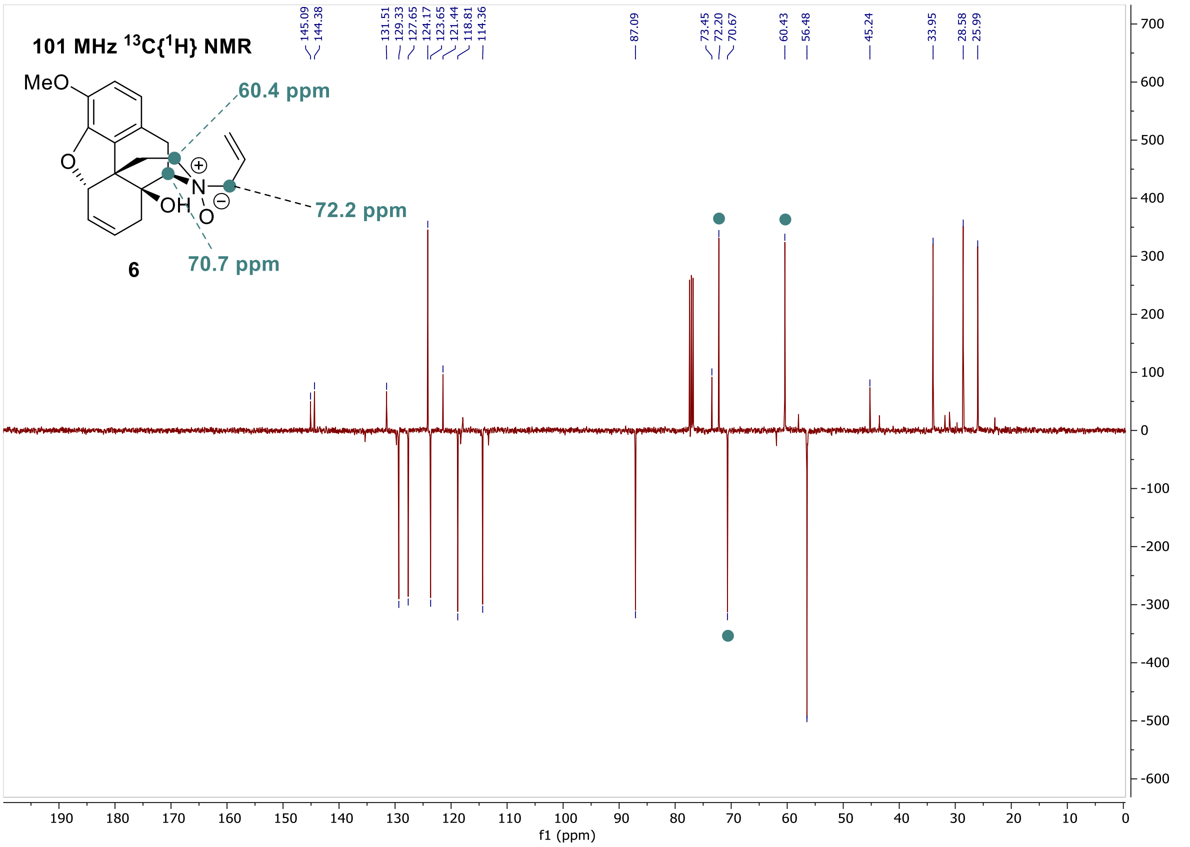

^{13}C spectra of naloxone methyl ether and the corresponding *N*-oxide

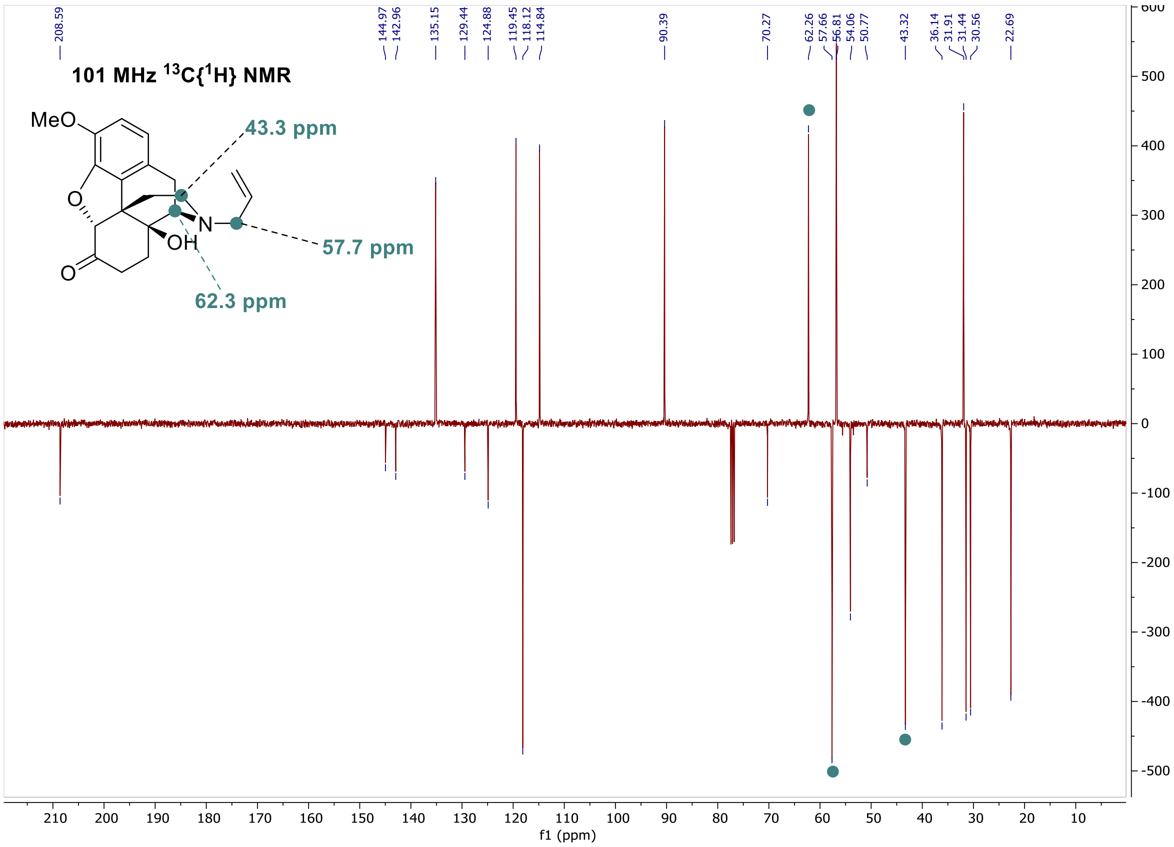

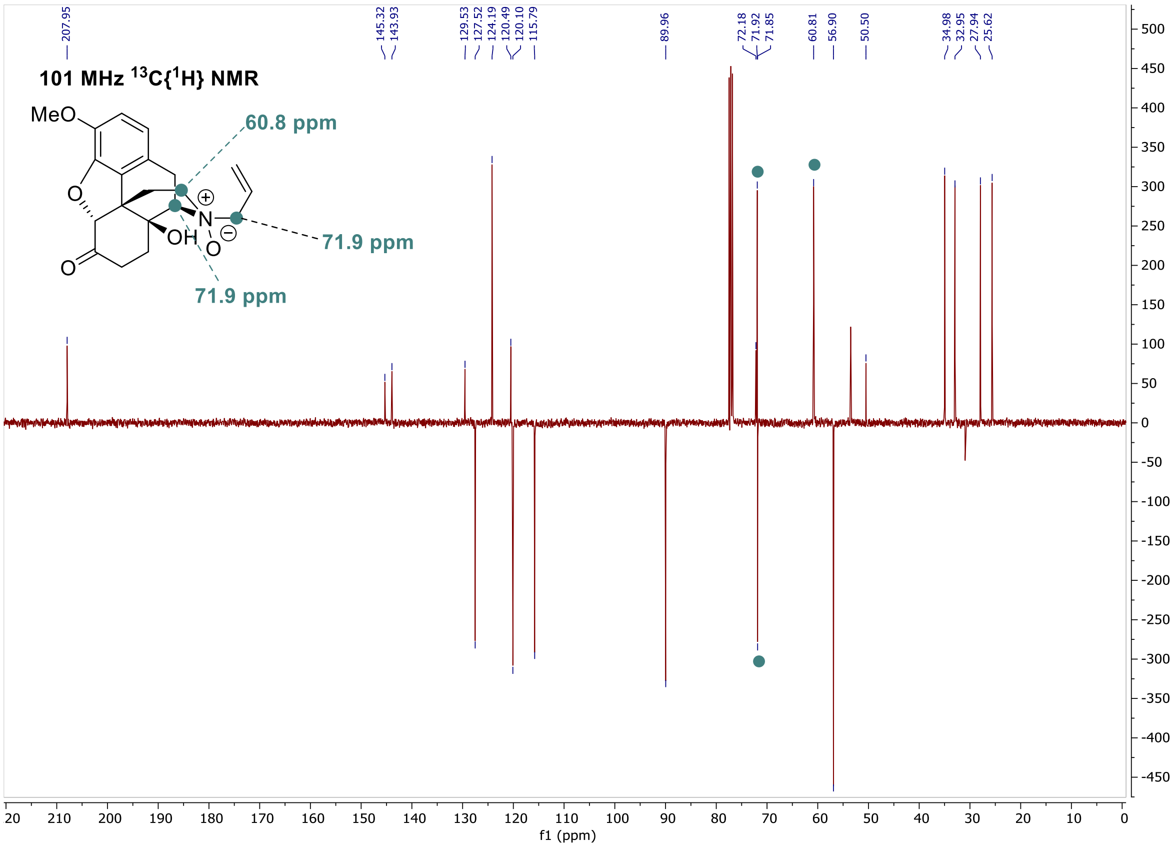

Supplement: Supplementary file 1 — jo5c00313_si_001.pdf [file jo5c00313_si_001.pdf]
